# On qualitizing revisited

**DOI:** 10.3389/fpsyg.2026.1818520

**Published:** 2026-07-16

**Authors:** Anthony J. Onwuegbuzie

**Affiliations:** Department of Educational Leadership and Management, University of Johannesburg, Johannesburg, South Africa

**Keywords:** 1 + 1 = 1 integration approach, data transformation, critical dialectical pluralism, methodological integration, mixed methods research, qualitative analysis, qualitative data, qualitizing

## Abstract

The original definition of *qualitizing*—the conversion of quantitative data into qualitative form—has made an important contribution to mixed methods research but presented a reductionist view that overlooked its interpretive and integrative dimensions. Building on Onwuegbuzie and Leech’s (2019) meta-framework, the present article reconceptualizes qualitizing as a recursive, meaning-centered, and meta-integrative process grounded in Critical Dialectical Pluralism (CDP) 2.0. Through this metaphilosophical lens, qualitizing is reframed not as a mechanical act of data conversion but as a meta-transformative process of interpretive synthesis that unites quantitative precision with qualitative depth across research traditions. To operationalize this expanded understanding, the article introduces a 43-profile *Narrative Profiling Taxonomy*—a theoretically grounded yet empirically generative framework for transforming quantitative, qualitative, or mixed data into interpretive narratives that embody full integration (in a representational sense). These profiles illustrate how numerical structures can be humanized through narrative representation, allowing meta-inferences that transcend analytic boundaries. Although extensive, the taxonomy is not presented as exhaustive but as an evolving representation of the current state of qualitizing’s methodological and epistemological potential. It introduces arts-based extensions highlighting the analytic, affective, and creative dimensions of integration. In re-envisioning qualitizing as a multidimensional system of meaning-making, *On Qualitizing Revisited* advances the integrative ideal of Onwuegbuzie and Hitchcock’s (2019) *1 + 1 = 1* integration approach, wherein data transformation becomes an act of synthesis rather than conversion. This redefinition positions qualitizing as both a methodological and philosophical foundation for integration, transforming data into meaning-centered understanding through integrative, interpretive processes that recontextualize analytic outputs within humanly interpretable forms.

## Crossover mixed analysis, data transformation, and the evolution of qualitizing in mixed methods research

One of the most powerful and unifying methodological developments within the field of mixed methods research is *crossover mixed analysis*, a term coined by Onwuegbuzie and Combs (2010) to describe analytic approaches that integrate quantitative and qualitative data within the same phase of analysis. Indeed, this led Teddlie and Tashakkori (2009) to declare that “we believe that this is one of the more fruitful areas for the further development of MM [mixed methods] analytical strategies” (p. 281). By explicitly linking data transformation, integration, and interpretation within a single analytic process, crossover mixed analysis has laid the groundwork for a new generation of mixed methods research approaches emphasizing meaning-making and full analytic integration.

Crossover mixed analysis enables researchers to cross traditional methodological boundaries—transforming, merging, and/or interpreting datasets in ways that yield more holistic insights than either tradition alone (Benge et al., 2018; Christensen et al., 2016; Grand-Guillaume-Perrenoud et al., 2023; Hitchcock and Onwuegbuzie, 2020; Johnson et al., 2025; Onwuegbuzie and Combs, 2010; Onwuegbuzie and Hitchcock, 2022; Onwuegbuzie et al., 2018; Onwuegbuzie and Johnson, 2021a, 2021b). Specifically, crossover mixed analysis enables research to address the nine purposes of analysis described by Onwuegbuzie and Combs (2010): to reduce, to display, to transform, to correlate/associate, to consolidate, to compare, to integrate, to assert, and/or to import data. It serves as a bridge between statistical inference and narrative understanding, making it central to mixed methods research in general and mixed methods data analysis in particular. Central to crossover mixed analyses are the twin processes of *quantitizing* and *qualitizing*, which allow researchers to transform data across representational forms—textual to numerical or numerical to textual—to achieve analytic integration. However, it is important to clarify that in its most foundational sense, qualitizing refers to the transformation of non-qualitative data—most commonly quantitative data—into qualitative, meaning-centered representations. This transformation involves not merely converting numerical values into textual form but recontextualizing such data within narrative, thematic, or interpretive frameworks that render them meaningful. At the same time, as articulated in the expanded definition of qualitizing (Onwuegbuzie and Leech, 2019, 2021), this process is not restricted to quantitative data alone but also can emerge from qualitative data through iterative and integrative analytic pathways. Thus, qualitizing is best understood as a meaning-centered process of transformation that is anchored in, but not limited to, the reinterpretation of non-qualitative data.

Narrative profiling as an analytic approach is not limited to this foundational form of qualitizing. When applied to qualitative data, narrative profiling does not constitute qualitizing in the strict sense of transforming non-qualitative data into qualitative meaning. Rather, it represents an extension of the same meaning-centered, integrative logic, further deepening, synthesizing, and re-representing qualitative insights. In such cases, narrative profiling functions as an advanced form of qualitative integration and meaning-making, enabling researchers to construct more coherent, contextually rich, and analytically unified representations of qualitative data. Thus, although qualitizing remains centered on the transformation of non-qualitative data, narrative profiling serves as a broader methodological framework that encompasses both qualitizing and the further integrative elaboration of qualitative findings. Taken together, these transformations of data across representational forms—through quantitizing and qualitizing—not only make data compatible across traditions, but also serve as the methodological foundation for full(er) integration, wherein quantitative precision and qualitative meaning converge within a unified analytic act.

The concept of *quantitizing* was first introduced in the social sciences by Tashakkori and Teddlie (1998), who described it as “the conversion of qualitative data to numbers using the ‘quantitizing’ technique described by Miles and Huberman (1994)” (p. 19). Their work built on earlier discussions of data transformation by Caracelli and Greene (1993). The term subsequently was operationalized by Onwuegbuzie and Teddlie (2003), who provided the first systematic framework for analyzing data in mixed methods research that explicitly included and labeled *quantitizing* as a core analytic transformation process. They operationalized quantitizing as the systematic conversion of qualitative data—such as codes, themes, or categories—into numerical form for the purposes of statistical analysis, pattern identification, and cross-case comparison. In their framework, quantitizing represented one of the key crossover strategies that enabled researchers to integrate data within or across analytic phases. This operationalization marked a critical shift from viewing quantitizing as an isolated technical step to conceptualizing it as a methodological bridge that facilitates inference generation and meta-integration across traditions.

The complementary concept, *qualitizing*, was also first introduced within the social sciences by Tashakkori and Teddlie (1998), who used the term to describe the conversion of quantitative data into qualitative form to facilitate integrated interpretation. Building on this initial conceptualization, Onwuegbuzie and Teddlie (2003) further elaborated *qualitizing* as a core process within the mixed methods data analysis process, positioning it as the reverse analytic pathway of *quantitizing*. *Qualitizing* involves transforming numerical or structured data into textual, narrative, or thematic representations, thereby allowing quantitative results to be interpreted through qualitative lenses. This process deepens understanding by emphasizing meaning, context, and the human dimensions underlying numerical patterns, making it essential for producing fully integrated (in a representational sense), interpretively rich meta-inferences in mixed methods research.

### Conceptual consolidation of quantitizing and qualitizing

The centrality of quantitizing was highlighted by Sandelowski et al. (2009), whose landmark discussion of quantitizing underscored its methodological importance in enabling the integration of qualitative data into statistical frameworks. These authors defined quantitizing as “the numerical translation, transformation, or conversion of qualitative data” (p. 208). They argued that quantitizing not only facilitates methodological triangulation, but also deepens interpretive insight by allowing the coexistence of numbers and narratives within a single analytic logic. Their contribution established quantitizing as a legitimate and essential technique for integrating meaning across traditions.

A decade later, Onwuegbuzie and Leech (2019, 2021) advanced the discussion of qualitizing in a similarly rigorous and expansive manner, addressing the historical imbalance in the methodological literature. Their efforts paralleled Sandelowski et al.’s (2009) work on quantitizing, but focused instead on the transformation of quantitative data into qualitative meaning—a process that had received far less conceptual or procedural attention.

In this regard, it is important to acknowledge related efforts that have emphasized the significance of achieving symmetry between quantitative and qualitative research approaches within mixed methods research. Notably, the late Emeritus Professor *M. Teresa* Anguera and her colleagues advanced this perspective within the context of observational methodology in the physical activity and sports sciences, advocating for greater integration and balance between the quantitative and qualitative dimensions of analysis. In their collaborative work with the present author (Anguera et al., 2017), these scholars highlighted the need to move beyond methodological asymmetries by developing approaches that facilitate the reciprocal transformation and integration of data forms. This contribution aligns closely with the conceptual foundations of qualitizing and further underscores the broader interdisciplinary relevance of efforts to achieve full(er) methodological integration.

Onwuegbuzie and Leech (2019) introduced the first formal meta-framework for qualitizing, defined as the process of converting quantitative data into forms suitable for qualitative analysis. Addressing a long-standing imbalance in the literature that had focused heavily on quantitizing, they conceptualized qualitizing as a cornerstone of full integration in mixed methods research, represented—as with quantitizing—by the methodological formula *1 + 1 = 1*, which was originally articulated by Onwuegbuzie (2017) and further developed by Onwuegbuzie and Hitchcock (2019) (see also Onwuegbuzie, 2022, 2023). Drawing on social constructionist assumptions that data are co-constructed rather than inherently quantitative or qualitative, they argued that qualitizing transforms numeric information into narrative meaning, thereby deepening interpretation, enhancing transparency, and democratizing evidence in the post-truth era. Their article advanced a typology for qualitizing data, delineating five major elements—its capacity (i) to yield multiple representational forms (e.g., codes, themes, metathemes, or narratives), (ii) to originate from either quantitative or qualitative data, (iii) to involve both qualitative and quantitative analyses, (iv) to occur as single or multiple iterative analyses, and (v) to produce fully integrated analyses. Through heuristic exemplars and detailed analytical mapping, the authors demonstrated how qualitizing not only complements quantitizing, but also redefines integration itself as an interpretive, reflexive, and creative act of mixed methods inquiry.

Notwithstanding these important contributions, several developments since the publication of Onwuegbuzie and Leech’s (2019) work warrant a revisitation and expansion of the concept of qualitizing. First, despite its conceptual promise, qualitizing remains markedly underutilized in the empirical literature, suggesting a need for further methodological elaboration and accessibility. Second, the original meta-framework, although foundational, invites extension into a more fully operationalized and integrative system that can guide researchers in practice. Third, emerging philosophical and methodological advancements—particularly the evolution of Critical Dialectical Pluralism (CDP) 2.0—provide an opportunity to reconceptualize qualitizing as a recursive, narrative, and meaning-centered process of meta-integration. Taken together, these developments underscore the intellectual and methodological necessity of revisiting and extending the 2019 framework.

Accordingly, the expansion from 5 to 43 narrative profile types presented in this article is not intended as a merely additive extension, but rather as a systemic elaboration of the underlying logic of qualitizing. This expansion aims to address specific gaps in the original framework by incorporating narrative forms that capture temporal dynamics, contextual embeddedness, cultural positioning, ethical reflexivity, and transformative processes of meaning-making. In this sense, the expanded taxonomy reflects a shift from representing isolated analytic functions toward modeling a multidimensional architecture of integration, wherein diverse forms of narrative reasoning collectively instantiate a broad and evolving range of qualitizing processes. Thus, the proposed typology responds directly to the need for a more comprehensive and operationalized system that aligns with the increasing complexity, pluralism, and reflexivity of contemporary mixed methods research practice.

### Revisiting the evolution and methodological expansion of quantitizing

Despite the foundational contributions of Tashakkori and Teddlie (1998) and the theoretical consolidation by Sandelowski et al. (2009), the empirical and methodological use of *quantitizing* has not increased substantially over the years. As noted by Onwuegbuzie (2025), an examination of publications in the *Journal of Mixed Methods Research* (*JMMR*) revealed that out of 346 empirical articles published from Volume 1, Issue 1 through Volume 18, Issue 2, only 55 (15.9%) included the word *quantitizing* or one of its variants (e.g., *quantitize, quantitized, quantitises, or quantitising*). A follow-up statistical trend analysis showed no linear, quadratic, cubic, or quartic pattern, indicating no clear increase in the use of the term, thereby suggesting that the practice and discussion of quantitizing remain sporadic despite its centrality to mixed methods integration.

According to Onwuegbuzie (2025), this limited uptake of quantitizing appears to have resulted from a lack of methodological development following Sandelowski et al.’s (2009) article. Methodological publications addressing *quantitizing* often reiterate its basic definition without substantially expanding its conceptual or procedural boundaries. Moreover, some scholars (e.g., Seltzer-Kelly et al., 2012) have raised concerns about the processes and outcomes associated with *quantitizing*, while others (e.g., Love and Corr, 2022) have proposed alternative analytic logics. Collectively, these factors may have constrained the evolution and widespread adoption of *quantitizing* as a mixed methods research technique.

In response to this conceptual stagnation, Onwuegbuzie (2025) authored *On Quantitizing Revisited* to reinvigorate and to extend the methodological foundations of the concept. In this article, Onwuegbuzie (2025) refined and expanded this construct through what he coined the *DIME-Driven Model of Quantitizing*, which conceptualizes *quantitizing* as a multi-level, meaning-centered act of data transformation situated within the broader *1 + 1 = 1* integration framework of Onwuegbuzie (2017) and Onwuegbuzie and Hitchcock (2019). The *1 + 1 = 1* integration framework provides the conceptual foundation for understanding the role of quantitizing within mixed methods research. Unlike additive models of integration (e.g., *1 + 1 = 3 approach*; Fetters and Freshwater, 2015), in which qualitative and quantitative components/phases/strands are integrated but remain analytically distinct, the *1 + 1 = 1* approach conceptualizes integration as a synthesis process in which data are transformed and reconstituted into a unified, meaning-centered whole. Within this framework, quantitizing functions not merely as a technical procedure for converting qualitative data into numerical form, but as a mechanism of transformation that contributes to the generation of integrated meta-inferences. Accordingly, the DIME-Driven Model operationalizes this logic by demonstrating how different forms of quantitizing serve as pathways through which qualitative meaning is systematically transformed, structured, and integrated within a broader interpretive framework.

Consistent with the acronym, the DIME-driven model comprises four interrelated forms:

- **D**escriptive quantitizing, involving the conversion of qualitative data into quantitative metrics (e.g., measures of central tendency, variation/dispersion, position/relative standing, and distributional shape) for descriptive characterization and pattern identification.
- **I**nferential quantitizing, in which qualitative data are transformed to enable general linear modeling (GLM) inferential analyses (e.g., multiple regression or analysis of variance) that allow prediction or generalization beyond the sample.
- **M**easurement quantitizing, which involves transforming qualitative observations into quantitative scales or indicators for measurement development and validation, using techniques such as Rasch modeling or item response theory (IRT).
- **E**xploratory quantitizing, which entails converting qualitative data into quantitative formats to reveal new structures or patterns through methods like exploratory factor analysis, cluster analysis, multidimensional scaling, and correspondence analysis.

Each phase of the model emphasizes transformation not as a mechanical conversion, but as a *meaning-making act* that enables integration across traditions. Through this typology, Onwuegbuzie’s (2025) framework redefines quantitizing as both a *methodological* and *philosophical* process—bridging numeric representation with narrative interpretation and reinforcing mixed methods’ integrative essence.

More broadly, Onwuegbuzie’s (2025) DIME-driven model underscores that the application and value of quantitizing extend beyond technical integration; they encompass qualitative research, quantitative research, *and* mixed methods research traditions. This article demonstrates how differing philosophical orientations—ontological, epistemological, axiological, and methodological—shape researchers’ stances toward quantitizing while emphasizing that few traditions reject its value entirely. Ultimately, Onwuegbuzie (2025) advocates for a balanced, reflexive use of quantitizing that complements qualitative analysis, enhances analytical transparency, and strengthens interpretive validity without diminishing the richness of qualitative meaning.

### Revisiting the evolution and methodological expansion of qualitizing

Despite its conceptual importance and potential for promoting full(er) integration in mixed methods research, the empirical and methodological use of *qualitizing* has been limited, lagging significantly behind that of *quantitizing*. At the time of the publication of Onwuegbuzie and Leech’s (2019, 2021) article, an extensive review of the existing methodological and empirical literature by these authors revealed not a single work devoted exclusively—or even in part—to the technique of qualitizing. Moreover, the authors found that there had been a statistically significantly greater mention of *quantitizing* than *qualitizing* in the literature, with *quantitizing* being 2.13 times more likely (95% confidence interval [CI] = 1.20–3.78) to appear than *qualitizing* in the 240 articles published in *JMMR* from its inception in 2007 to 2019. When disaggregated by article type, only 6.9% of the *JMMR* empirical research articles and 10.5% of the *JMMR* methodological or theoretical articles contained the words *qualitize* and/or *qualitizing*. These proportions likely represent an upper bound for the broader literature, given that *JMMR*—with its high impact factor and ranking at the time—tends to publish articles authored by highly experienced scholars, with Onwuegbuzie and Leech (2019) reporting that the average years of postdoctoral publishing experience was 13.67. Interestingly, Onwuegbuzie and Leech (2019) identified no statistically significant relationship between the years of publishing experience of the lead author and whether the article contained *qualitize* or *qualitizing*, suggesting that author seniority does not predict engagement with the qualitizing construct.

Following this systematic review, two methodological works by Onwuegbuzie and Leech (2019, 2021) formally introduced and expanded the concept of *qualitizing*, helping to fill a major theoretical and procedural gap in mixed methods research. These publications provided the first comprehensive articulation of qualitizing as a process of transforming quantitative data into forms suitable for qualitative analysis, situating it as a core component of crossover mixed analysis (Hitchcock and Onwuegbuzie, 2020) and of the *1 + 1 = 1* integration approach (Onwuegbuzie, 2017; Onwuegbuzie and Hitchcock, 2019, 2022). Their work also produced the first meta-framework for qualitizing, including five key elements that position it as both a technical and interpretive act capable of producing fully integrated meta-inferences.

Nevertheless, the empirical and methodological uptake of qualitizing remains limited compared to quantitizing. Several factors might account for this ongoing disparity. First, historical precedence and familiarity bias favor quantitizing: Because researchers have long been trained in quantitative techniques (Capraro and Thompson, 2008; Leech and Goodwin, 2008), transforming qualitative data into numbers might feel more intuitive, concrete, and statistically defensible. In contrast, converting quantitative data into narratives can be perceived as less rigorous or less replicable (cf. Nzabonimpa, 2018).

Second, epistemological asymmetry continues to shape research cultures; many scholars, particularly in quantitatively oriented disciplines, view qualitative transformation as interpretive rather than purely technical or procedural (Onwuegbuzie and Leech, 2019), thereby undervaluing its methodological legitimacy. Importantly, the distinction drawn here between *interpretive* and *analytic* (i.e., purely technical or procedural) is not intended to position these processes as mutually exclusive. Rather, it reflects a differentiation between analysis understood as primarily technical, procedural, or reductionist and analysis understood as a meaning-centered, interpretive act. Within qualitative inquiry—and within the framework advanced in this article—analysis is inherently interpretive, involving the construction, contextualization, and synthesis of meaning rather than mere categorization or transformation of data. Thus, the emphasis on interpretive transformation is meant to foreground the depth, reflexivity, and meaning-making dimensions of analysis, rather than to suggest a separation between analytic and interpretive processes. This clarification aligns with the broader argument advanced in this article that narrative profiling dissolves traditional dichotomies by demonstrating that analysis and interpretation are integrative, co-constitutive processes.

Third, limited methodological development and training have meant that most mixed methods research curricula, in general, and analytic frameworks, in particular, have lacked formal instruction in qualitizing procedures. Finally, conceptual inertia has reinforced this imbalance: Because *quantitizing* has been more extensively operationalized and demonstrated (Cabrera and Reiner, 2018; Collingridge, 2013; Driscoll et al., 2007; Isaac et al., 2016; Kerrigan, 2014; Leal et al., 2018; Miles and Huberman, 1994; Onwuegbuzie, 2025; Onwuegbuzie and Teddlie, 2003; Sandelowski et al., 2009; Tashakkori and Teddlie, 1998; Wao et al., 2011; Weaver-Hightower, 2014), it has had a greater opportunity to become normalized in both methodological practice and scholarly discourse.

However, the works of Onwuegbuzie and Leech (2019, 2021) mark a turning point in the evolution of qualitizing. Their meta-framework not only represents an important step towards counterbalancing the field’s historic emphasis on quantitizing, but also advances qualitizing as a rigorous, reflexive, and interpretive act that generates meaning from numerical data. In so doing, their work situates qualitizing at the heart of full integration.

Nevertheless, despite these advancements, the empirical uptake of qualitizing continues to lag noticeably behind that of quantitizing. A systematic review of the *JMMR* revealed that among the 130 articles (excluding editorials, media reviews, notes, and errata) published in the six full years since the release of *On Qualitizing* (Onwuegbuzie and Leech, 2019)—that is, from Volume 14, Issue 1 (2020) through Volume 19, Issue 4 (2025)—only 12 articles included the term *qualitizing* or one of its variants. This frequency represents 9.23% (12 of 130) of all *JMMR* article publications during this period that engaged with the concept to any degree. Yearly frequencies (4 articles in 2020, 1 in 2021, 4 in 2022, 1 in 2023, 1 in 2024, and 1 in 2025) reveal no discernible temporal trend, suggesting that the use of *qualitizing* remains sporadic rather than steadily increasing. Indeed, it appears that the prevalence of discussion of qualitizing has diminished in the most recent years, indicating a possible waning of methodological attention despite its growing theoretical importance. By comparison, 18 articles (13.85%) published in this same 6-year span referenced *quantitizing* or one of its variants—a modestly higher proportion, although this difference is not statistically significant (χ^2^[1, *N* = 130] = 1.36, *p* = 0.24; Fisher’s Exact *p* = 0.33; odds ratio = 1.58, 95% CI = 0.73, 3.43). Collectively, these findings indicate that although both processes remain underrepresented in the mixed methods research literature, quantitizing continues to hold a slight edge in methodological discourse, reflecting the enduring imbalance between numerical translation and narrative transformation in mixed methods inquiry.

Therefore, to help motivate more mixed methods researchers to recognize and to harness the benefits of *qualitizing*, there is a pressing need for additional methodological publications devoted specifically to this topic. Just as Onwuegbuzie’s (2025) *On Quantitizing Revisited*—which itself involved revisiting the topic of *quantitizing*—extended Sandelowski et al.’s (2009) conceptual analysis of *quantitizing* (which had centered on its philosophical assumptions and interpretive complexities) by transforming the construct into a fully operationalized, multi-level methodological framework (i.e., the DIME-Driven Model) that shifted the discourse from critical reflection to structured methodological praxis and integrative application across research traditions, there is also a need to revisit the topic of *qualitizing* by expanding Onwuegbuzie and Leech’s (2019) *On Qualitizing*. The present article responds to this need by advancing and extending the concept of *qualitizing*, offering an extensive and integrative framework that elevates it from an underdeveloped analytic transformation to a meta-framework for meaning-making and methodological synthesis.

## Purpose

Given that the transformation of qualitative data into quantitative form has been prioritized over the equally rich and interpretively powerful process of transforming quantitative data into qualitative meaning, the conceptual, methodological, and applied aspects of qualitizing clearly warrant further elaboration and expansion. In particular, there remains a critical need to articulate the philosophical grounding, procedural rigor, and integrative potential of qualitizing within contemporary mixed methods research practice. The present article extends this line of inquiry, building on the foundation laid by Onwuegbuzie and Leech (2019, 2021) and by the integrative logic of crossover analysis (Onwuegbuzie and Combs, 2010).

Specifically, the purpose of this article is twofold. First, it revisits the conceptual and methodological architecture of *qualitizing* by demonstrating how narrative profiles embody the five major elements of qualitizing. Second, it presents a comprehensive typology of 43 narrative profile types—ranging from modal and holistic profiles to transformative, resilience, and cultural profiles—as the most advanced forms of data representation in mixed methods research. The follow-up article extends this framework by situating narrative profiles at the very center of qualitizing practice. Narrative profiles, as first introduced by Tashakkori and Teddlie (1998), provide a concrete, systematic, and philosophically grounded way of producing qualitative narratives from quantitative, qualitative, or integrated datasets. Drawing on empirical examples, this article illustrates how narrative profiling transforms numerical precision into storied meaning, thereby achieving full integration—what Onwuegbuzie (2017) and Onwuegbuzie and Hitchcock (2019) referred to as the *1 + 1 = 1* integration approach. As such, the present article responds directly to this imbalance by advancing the theoretical, methodological, and representational foundations of qualitizing and positioning narrative profiling as its most integrative expression.

To strengthen further the procedural rigor of qualitizing, it is important to clarify that narrative profiling is not an intuitive or purely impressionistic activity, but rather a systematic and transparent analytic process. Specifically, the development of narrative profiles involves a sequence of interrelated steps, including (a) identifying salient quantitative, qualitative, or mixed data patterns; (b) selecting an appropriate narrative profile type aligned with the analytic purpose; (c) translating these patterns into narrative form using explicit interpretive logic; and (d) iteratively refining the profile to ensure coherence, fidelity to the data, and alignment with the underlying philosophical framework. In this sense, narrative profiling functions as a structured methodological procedure that integrates analytic precision with interpretive depth, thereby enhancing both the transparency and reproducibility of qualitizing-based analyses.

Furthermore, careful attention must be paid to ethical considerations when constructing individual-level narrative profiles. Rich, detailed narratives—particularly those derived from a single participant—can increase the risk of identifiability, especially in small or contextually bounded samples. Accordingly, narrative profiling should incorporate strategies such as abstraction, compositing, or selective generalization to protect participant confidentiality while preserving analytic meaning. This reinforces the principle that qualitizing is not only a methodological process but also an ethically grounded representational practice.

In addition, it is important to emphasize that the contribution of qualitizing through narrative profiling extends beyond conventional narrative description or discussion-based interpretation. Whereas traditional discussion sections typically contextualize findings *after* separate analyses have been completed, narrative profiling embeds integration within the analytic process itself by transforming data into unified, meaning-centered representations.

It is important to distinguish qualitizing through narrative profiling from the type of narrative contextualization typically found in the Discussion sections of research reports. Whereas Discussion sections are generally retrospective and interpretive—serving to explain, to contextualize, or to theorize findings after analyses have been completed—narrative profiling constitutes an integral component of the analytic process itself. That is, narrative profiles are generated through the systematic transformation of data into structured, interpretive representations guided by explicit analytic decisions, rather than being constructed as post-hoc reflections on results. Consequently, qualitizing through narrative profiling operates at the level of analysis rather than commentary, thereby positioning integration not as an outcome of interpretation, but as a methodologically enacted process.

This distinction highlights the unique contribution of narrative profiling as a methodological approach. Unlike traditional Discussion sections, which often synthesize separate quantitative and qualitative findings in a discursive manner, narrative profiling generates unified representations in which analytic integration is achieved within the narrative form itself. In so doing, it enables researchers to represent complex patterns, relationships, and processes in ways that preserve contextual richness while maintaining analytic coherence and transparency. Moreover, because narrative profiles are explicitly tied to identifiable analytic procedures and profile types, they offer a level of structure, replicability, and methodological accountability that extends beyond conventional narrative interpretation. Thus, qualitizing through narrative profiling should be understood not as an extension of discussion, but as a distinct and systematic mode of data analysis that transforms how integration is conceptualized and operationalized in mixed methods research. Qualitizing through narrative profiling is not merely a descriptive or interpretive add-on, but a distinct methodological approach that operationalizes integration as a core analytic act. Crucially, the full analytic potential of narrative profiling—and its alignment with qualitizing—is realized at the group or cross-case level. Whereas individual profiles can provide insight into particular experiences, qualitizing requires the transformation of data across cases into integrated, meaning-centered representations. This involves identifying patterns, relationships, or structures that transcend individual accounts and synthesizing them into higher-order narrative forms. Therefore, the subsequent illustrations shift from individual-level representation toward group-level narrative synthesis, wherein integration is enacted through the construction of composite, comparative, or meta-narrative profiles.

To illustrate more concretely how group-level narrative synthesis embodies critical dialectical pluralism, it is necessary to clarify the analytic transformations underlying these representations. Specifically, the group-level narrative profiles presented here are derived from the identification of cross-case patterns, thematic convergence and divergence, and the integration of multiple data sources into unified interpretive accounts. These profiles do not merely juxtapose individual voices; rather, they synthesize them into higher-order meaning systems that reflect both shared structures and productive tensions among participants. In this way, group-level narrative synthesis operationalizes qualitizing by transforming distributed data into coherent, integrative narratives that embody Onwuegbuzie and Hitchcock’s (2019) *1 + 1 = 1* logic of full integration. This distinction underscores the added value of the present framework in advancing mixed methods research toward more coherent, transparent, and meaning-centered forms of inquiry.

Taken together, these illustrations demonstrate that the value of narrative profiling lies not only in the descriptive richness of individual accounts, but also in the capacity to transform data into analytically integrated representations. By moving from individual-level description to group-level synthesis, the heuristic illustration more fully reflects the principles of qualitizing as a process of meaning-centered integration rather than narrative restatement.

This article holds particular significance for the field of psychology and its related disciplines, wherein the challenge of uniting empirical precision with interpretive understanding remains central to research and practice. By demonstrating how structured data can be transformed into meaning-rich narratives, this meta-framework offers psychologists, counselors, and behavioral scientists a way to represent human complexity with both analytic rigor and contextual depth. Beyond psychology, the ideas advanced herein possess broad interdisciplinary utility: Across the social sciences, health and medical research, education, and the humanities, the creation of narrative profiles provides a systematic means for transforming data into coherent, human-centered accounts of experience. In this way, the framework proposed in this article offers a unifying approach to data interpretation that can enrich inquiry across virtually all fields concerned with understanding human meaning and behavior.

## Philosophical framework: critical dialectical pluralism (CDP) 2.0

Serving as the meta-transformative and multidimensional philosophical foundation for this article, Critical Dialectical Pluralism (CDP) 2.0 (Onwuegbuzie and Abrams, 2025; see also Onwuegbuzie and Frels, 2013, for its predecessor, CDP 1.0) provides the guiding metaparadigm and metaphilosophy through which the process of qualitizing and narrative profiling is conceptualized. CDP 2.0 is anchored in three metaprinciples—*criticality*, *dialectical engagement*, and *pluralism*—and is supported by five foundational pillars: **s**ocial **j**ustice, **i**nclusivity, **d**iversity, **e**quity, and **s**ocial responsibility (SIDES). Together, these pillars embody a reflexive and ethically grounded orientation that democratizes inquiry and foregrounds participant voice. CDP 2.0 also encompasses 10 interrelated metaphilosophically informed dimensions—**C**ultural, **L**ife, **E**nvironmental, **A**cademic, **R**esearch, **S**piritual, **T**heoretical, **E**thical, **P**olitical, and **S**ocietal (CLEAR STEPS)—that collectively frame research as a value-infused and socially responsive endeavor. As a meta-transformative and multidimensional metaparadigm and metaphilosophy, CDP 2.0 aligns seamlessly with the goal of narrative profiling: to integrate quantitative precision and qualitative meaning into unified, equitable, and human-centered representations of experience.

Within this study, CDP 2.0 functions not only as a philosophical foundation, but also as a meta-methodological compass for integration. Its dialectical principle fosters synthesis across quantitative and qualitative forms of knowing, while its pluralistic orientation legitimizes multiple epistemic and methodological voices within a single analytical frame. The critical dimension ensures that the construction of narrative profiles remains ethically responsive and socially conscious—attuned to issues of equity, inclusion, and representation. In this way, CDP 2.0 does not merely frame qualitizing; it enacts it, turning integration itself into a transformative act of meaning-making.

Unlike mixed methods research philosophies that emphasize *methodological pragmatism* [e.g., *Pragmatism-of-the-Middle* (Johnson and Onwuegbuzie, 2004; Johnson et al., 2007); *Pragmatism-of-the-Right* (Putnam, 2002; Rescher, 2000); *Pragmatism-of-the-Left* (Maxcy, 2003; Rorty, 1991); *Transformative–Emancipatory* (Mertens, 2003, 2007, 2010; Mertens et al., 2010); *Dialectical Pluralism* (Johnson, 2012, 2017; Johnson et al., 2014; Tucker et al., 2020)] those grounded in *epistemic relativism* [e.g., *Phenomenography* (Feldon and Tofel-Grehl, 2022); *Dialectical Stance* (Greene, 2007, 2008; Greene and Caracelli, 1997; Greene and Hall, 2010; Maxwell and Loomis, 2003), or *Communities of Practice* (Denscombe, 2008)], CDP 2.0 foregrounds the ethical, ontological, and social justice dimensions of inquiry. CDP 2.0 researchers view integration not as a technical synthesis or pragmatic alignment but as a dialogical and value-infused act that bridges multiple perspectives while interrogating power, privilege, and inequity in knowledge construction. In so doing, CDP 2.0 extends beyond methodological convenience or epistemic contingency to embrace a meta-transformative stance, positioning *qualitizing*—and the creation of narrative profiles—as processes of ethical engagement, reflexive understanding, and emancipatory meaning-making.

The present article introduces what can be characterized as a *Critical Dialectical Pluralism Meta-Framework for Qualitizing-Based Integration*. Situated within the philosophical tenets of CDP 2.0 (Onwuegbuzie et al., 2011), this meta-framework operates at a level above traditional conceptual or theoretical frameworks. It integrates philosophical, methodological, analytical, and ethical dimensions into a unified, meta-transformative structure that guides the process of qualitizing and narrative profiling. The article is multi-family in scope—meeting criteria from the *foundational* (frameworks that establish the philosophical, ontological, epistemological, and axiological grounding of inquiry), *methodological and analytical* (frameworks that guide the design, data collection, and analytic strategies of research), *cross-cutting and critical* (frameworks that interrogate power, equity, ethics, and reflexivity across paradigms and methodologies), and *structural/process* (frameworks that delineate the organization, sequencing, and integration of research processes and meaning-making) families of frameworks simultaneously—representing four of the 23 framework families identified by Onwuegbuzie and Leech (2025). Drawing on the five major elements of qualitizing (Onwuegbuzie and Leech, 2019, 2021) and operationalizing them through 43 narrative profile types, this meta-framework functions across traditions—quantitative research, qualitative research, and mixed methods research—while remaining anchored in the pluralist and critical ethos of CDP 2.0. As such, it provides researchers with an overarching architecture for synthesizing data, analysis, and interpretation into a coherent whole, thereby transforming integration itself into an act of reflexive, ethical, and human-centered meaning-making.

## Conceptual foundations: from on qualitizing to on qualitizing revisited

### Foundational premise: narrative profiles as a mode of qualitizing

Constructing narrative profiles represents one of the most effective, human-centered, and meaning-oriented modes of *qualitizing* data. Through this process, numerical data are transformed into descriptive, story-like representations that maintain statistical rigor while deepening interpretive and contextual meaning. In so doing, narrative profiling transforms quantitative results—traditionally confined to statistical tables or figures—and qualitative findings—often reported as (insufficiently) decontextualized codes, subthemes, themes, metathemes, or categories—into interpretive portraits that communicate lived experience, motivation, and nuance. Importantly, the interpretive power of narrative profiling should not be understood as providing direct access to participants’ *lived realities* or as restoring participant voice in any unmediated sense. Rather, narrative profiles constitute researcher-constructed representations that recontextualize data within coherent, meaning-centered accounts. In this respect, narrative profiling does not reproduce participant experience but instead offers an interpretive synthesis that renders patterns, relationships, and implications of the data more intelligible. The value of this approach lies not in claiming epistemic transparency but in its capacity to situate quantitative and qualitative findings within analytically grounded narrative forms that highlight their contextual significance and interpretive consequence. This transformation recontextualizes data by situating it within storied meaning systems that reflect the complexity of human experience. In both cases, whether translating numbers into narrative or weaving themes into coherent storylines, narrative profiling yields representational forms that transcend isolated analytic traditions, integrating precision with depth and abstraction with embodiment. This approach functions as a bridge between postpositivist and interpretivist paradigms: It retains the precision and generalizability valued in quantitative traditions while embracing the contextual sensitivity and narrative richness central to qualitative inquiry. Grounded in the metaprinciples and pillars of CDP 2.0, it reflects the fact that data do not “speak” on their own but acquire meaning only through interpretive narration and contextualization.

Tashakkori and Teddlie (1998) first formalized this transformative process by identifying five foundational types of narrative profiles—*modal, average, holistic, comparative,* and *normative*—which collectively offered a pioneering typology for qualitizing quantitative results. Each type represents a distinct way of translating numerical patterns into narrative understanding, thereby humanizing the act of data representation.

#### Modal narrative profile

The *modal profile* represents the most frequently occurring characteristics or attributes within a dataset, essentially the statistical *mode* recast as narrative. It portrays the *most common* participant or phenomenon in story form, highlighting dominant tendencies or recurrent experiences. For instance, in a study of teacher motivation, a modal profile might read, “The typical teacher in this sample reports high intrinsic motivation, moderate work stress, and consistent job satisfaction, describing her professional identity as driven by student growth and classroom creativity.” This profile translates central quantitative frequencies into qualitative essence, capturing the most representative experience while still acknowledging individual variation.

- Strengths: It offers clarity, simplicity, and recognizability; it easily links descriptive statistics to narrative understanding.
- Limitations: It risks oversimplification or erasure of minority or divergent experiences; best suited for homogeneous datasets. To mitigate this, the modal profile should be presented *alongside* the original quantitative findings from which it was derived, allowing readers to see how the narrative abstraction reflects and simplifies the empirical distributions.

#### Average narrative profile

The *average profile* is derived from the mean of multiple quantitative attributes, presenting a composite or “typical” case based on aggregated data. It conveys what the central tendency of a group *looks like* when expressed as a single narrative portrait. For example, in a psychological study on resilience, an average profile might be as follows: “On average, participants reported moderate resilience, moderate perceived stress, and average emotional well-being, describing themselves as coping adequately but not exceptionally in the face of challenges.” This profile synthesizes group-level data into a single interpretive case, representing a balanced but abstracted “average person.”

- Strengths: It captures general trends and central tendencies in human terms; useful for conveying typicality.
- Limitations: It might obscure variability or mask meaningful outliers; it operates under the assumption that the “average” experience adequately represents the group. Because of this abstraction, the average profile should be *accompanied* by the original statistical summaries or illustrative cases from which it was derived, ensuring that readers can trace how the narrative generalization relates to the underlying data distribution.
- Although modal and average narrative profiles share the common goal of summarizing central tendencies within a dataset, they differ in important ways that have implications for their use. The modal profile emphasizes the most frequently occurring characteristics, thereby highlighting dominant or typical patterns; however, it might obscure variability and overrepresent the most common category at the expense of less frequent but substantively important variation. In contrast, the average profile synthesizes data across cases to produce a more balanced representation of central tendency, but in doing so, it might smooth over meaningful distinctions and mask heterogeneity within the data. Therefore, both profiles provide accessible and parsimonious summaries while also sharing the limitation of potentially reducing complexity. Accordingly, the choice between modal and average profiles should be guided by the researcher’s analytic purpose—whether the emphasis is on identifying dominant patterns or on representing aggregate tendencies across cases.

#### Holistic narrative profile

The *holistic profile* integrates data across multiple dimensions or domains—often combining quantitative and qualitative indicators—to construct a multidimensional understanding of a participant, group, or phenomenon. Rather than focusing on central tendency, it emphasizes contextual coherence and relational depth. For example, in a mixed methods research study of immigrant adjustment, a holistic profile might integrate survey results, interview narratives, and observation data to tell the story of a participant whose quantitative acculturation score is linked to qualitative themes of belonging, nostalgia, and identity negotiation. This approach aligns with interpretivist logics by privileging complexity and interconnectedness.

- Strengths: It promotes comprehensive understanding; it also allows nuanced integration of diverse data sources.
- Limitations: It requires greater interpretive skill and is less generalizable due to its idiographic focus. Given its integrative nature, the holistic profile should be presented *alongside* a clear account of the data sources and analytic steps that informed it, enabling readers to trace how multiple forms of evidence were synthesized into a coherent narrative representation.
- To clarify further, although holistic narrative profiles are frequently constructed from the integration of quantitative and qualitative data, such integration is not a definitional requirement. Rather, the defining feature of the holistic profile lies in its multidimensional and integrative orientation—namely, the synthesis of multiple domains, constructs, or layers of meaning into a coherent narrative representation. As such, holistic profiles can be generated from quantitative data alone (e.g., integrating multiple measured variables into a unified interpretive portrait), from qualitative data alone (e.g., synthesizing themes across interviews or observations), or from mixed datasets that combine both forms. In this way, the holistic narrative profile is best understood not as being defined by its data source but by the scope and depth of its integrative logic.

#### Comparative narrative profile

The *comparative profile* juxtaposes two or more cases, groups, or conditions to highlight similarities and differences across contexts. It represents the comparative logic of mixed methods research in narrative form. For example, in an educational study, a comparative profile might contrast “high-performing schools, where teachers describe strong collaborative cultures and high student engagement,” with “low-performing schools, where staff cite administrative instability and emotional fatigue.” This type of profile translates comparative statistical analysis (e.g., *t* tests or analysis of variance) into interpretive contrastive narratives, making between-group differences conceptually vivid.

- Strengths: It promotes cross-case insight and reveals patterns of convergence and divergence. Importantly, the comparative narrative profile is intended to encompass both convergence and divergence across cases or contexts. That is, it not only highlights contrasts or differences, but also identifies shared patterns, common structures, or parallel experiences that emerge across participants, groups, or settings. For example, a comparative profile might reveal that despite differences in institutional context, instructors share similar commitments to fostering reflexivity or methodological integration, thereby illustrating convergence alongside divergence. In this way, comparison functions as an inclusive analytic process that integrates similarity and difference within a single interpretive frame rather than privileging one over the other.
- Limitations: It might, if not carefully constructed, overemphasize differences at the expense of shared patterns or conversely, overgeneralize similarities in ways that obscure meaningful contextual distinctions, thereby requiring careful attention to contextual balance. To maintain interpretive fidelity, the comparative profile should be *accompanied* by the underlying comparative data—quantitative summaries and/or qualitative excerpts—that substantiate the narrative contrasts, ensuring that distinctions are evidence-based rather than rhetorically overstated.

#### Normative narrative profile

The *normative profile* positions individual or group data relative to a benchmark, standard, or ideal—often using z scores or percentile ranks to describe qualitatively deviations from a reference point. In this context, the term *normative* encompasses a range of reference points, including empirical benchmarks, expected standards, and idealized criteria, all of which serve as bases for evaluating or situating individual or group data within a broader frame of meaning. It answers the question, “How does this case compare to what is expected or ideal?” For example, in a clinical psychology context, a normative profile might describe a patient’s wellbeing as “one standard deviation below the normative average for emotional health among adults aged 30–40, characterized by moderate anxiety and reduced social engagement.” The narrative thus embeds normative comparison within personal context, making statistical deviation meaningful in human terms.

- Strengths: It integrates evaluative or diagnostic interpretation and bridges statistical positioning with evaluative interpretation by situating data relative to benchmarks, standards, or ideals. This bridging function is distinctive in that the normative narrative profile does not merely translate numerical results into descriptive or thematic meaning but interprets them in relation to explicit or implicit standards of evaluation. For example, statistical indicators such as percentile ranks or standard scores become narratively meaningful when framed in terms of meeting, exceeding, or falling short of expected or ideal benchmarks. In this way, the normative profile uniquely integrates quantitative positioning with evaluative interpretation, transforming statistical comparison into contextually grounded judgments about performance, status, or alignment with reference criteria.
- Limitations: It risks reinforcing normative bias or deficit framing if not reflexively applied and is contextually dependent. Therefore, to ensure transparency and interpretive fairness, the normative profile should be presented *alongside* explicit information about the benchmarks or standards used for comparison, as well as the rationale for their selection, allowing readers to assess the cultural and contextual appropriateness of the normative frame.

#### Comparison and integration of the five foundational profile types

Although all five profile types exemplify qualitizing by converting quantitative data into narrative meaning, they differ in epistemic orientation and purpose. The *modal* and *average* profiles are primarily *descriptive*, reflecting group-level tendencies through narrative synthesis. The *holistic* and *comparative* profiles are *analytic and interpretive*, emphasizing relationships, contrasts, and context. Meanwhile, the *normative* profile is *evaluative*, situating data relative to standards or expectations. Together, these five types form a methodological continuum—from statistical summarization to interpretive integration—demonstrating the versatility of narrative profiling as both a descriptive and an inferential act.

Importantly, the designation of the five foundational profiles as a distinct family is not based solely on their historical precedence but reflects their unique epistemic role within the taxonomy. Specifically, these profiles establish the core representational logics—descriptive, aggregative, integrative, comparative, and evaluative—that underpin all subsequent narrative profile types. In this sense, they function as conceptual anchors within the broader framework, providing the baseline modes of narrative reasoning from which more specialized, contextually embedded, and analytically complex profiles emerge. Although certain foundational profiles (e.g., normative or comparative) share affinities with later profile families, their inclusion within the foundational category highlights their role in establishing the primary dimensions of qualitizing rather than their alignment with any single downstream analytic purpose. Accordingly, the taxonomy is structured not only as a set of categories, but also as a developmental continuum, wherein foundational profiles represent the entry points into narrative integration, and the subsequent families represent progressive elaborations of these core logics.

Notwithstanding their foundational importance, these five narrative profile types also exhibit several limitations that constrain their capacity to capture the full scope of meaning-centered integration in contemporary mixed methods research. Specifically, they primarily emphasize static representations of central tendency, comparison, or normative positioning, thereby offering limited capacity to represent dynamic processes such as development, transformation, adaptation, or resilience. In addition, they do not explicitly accommodate the increasing importance of contextual, cultural, ethical, and relational dimensions of inquiry that have emerged as central concerns across the social, behavioral, and health sciences. Furthermore, although these profiles demonstrate how quantitative findings can be rendered narratively, they provide comparatively less guidance for representing complex, multi-layered integrations that involve recursive, multi-phase, or meta-analytic forms of reasoning. As such, the original typology, while pioneering, remains necessarily partial in its ability to reflect the expanding epistemological and methodological landscape of mixed methods research.

The present article builds on Tashakkori and Teddlie’s (1998) important foundation by extending their typology into an expanded and multidimensional taxonomy of 43 narrative profiles, encompassing new families such as *temporal, cultural, transformative, ethical, and resilience-based* profiles. This expansion transforms the original framework into a philosophically plural, reflexive, and value-infused model of data transformation—one that operates fluidly across quantitative research, qualitative research, and mixed methods research traditions. Through this expansion, narrative profiling emerges not only as a technique for qualitizing data, but also as a meta-framework for representing complexity, contextuality, and human experience in mixed methods research integration.

## Reframing the five elements of qualitizing through narrative profiling

As noted previously, Onwuegbuzie and Leech’s (2019, 2021) formulation of *qualitizing* established five major elements that together defined the process of transforming quantitative data into qualitative meaning: (i) the generation of multiple representational forms, (ii) the bidirectional potential between data types, (iii) the dual engagement of qualitative and quantitative analyses, (iv) the iterative nature of analytic transformation, and (v) the achievement of full integration as an interpretive end point. In the present article, I retain this foundational architecture but reinterpret each element through the lens of *narrative profiling*—a representational mode that recasts data transformation as a process of storying, contextualizing, and humanizing information. In this reframed perspective, qualitizing is not only a methodological operation, but also a narrative act that integrates inference and interpretation.

1 Multiple representational forms

The first element, originally emphasizing qualitizing’s capacity to yield codes, themes, or narratives, is expanded in this meta-framework into a comprehensive representational taxonomy comprising 43 distinct narrative profile types. Each profile type translates data into a specific form of storied meaning—ranging from modal, average, and holistic profiles to transformative, ethical, or cultural ones. Thus, narrative profiling extends the principle of representational multiplicity from *forms of data* to *forms of meaning*, demonstrating that data can be narrated in ways that preserve statistical precision while revealing interpretive depth.

2 Bidirectionality of transformation

Whereas Onwuegbuzie and Leech (2019) framed qualitizing as a process of converting quantitative data into qualitative expression, narrative profiling reconceptualizes this movement as *reciprocal*. Through narrative logic, quantitative data are qualitized into interpretive portraits, and qualitative narratives, in turn, can be re-structured into quantifiable or patterned representations (e.g., thematic frequencies or narrative archetypes). This reciprocity underscores that the boundary between quantitizing and qualitizing is interpretively constructed rather than ontologically fixed—each mode capable of transforming the other through narrativization.

3 Dual analytical modes

The third element—integrating qualitative and quantitative analyses—is deepened through the introduction of *narrative inference*. Within narrative profiling, the construction of profiles involves both statistical reasoning (identifying distributions, contrasts, or benchmarks) and interpretive reasoning (inferring meaning, context, and perspective). Therefore, narrative profiling operationalizes the coexistence of analytic modes, uniting the inferential logic of the quantitative with the hermeneutic logic of the qualitative into a single interpretive synthesis.

4 Iterative processes

Qualitizing always has been iterative, but narrative profiling makes this explicit through the *layered* construction of profiles. Profiles are rarely written once; rather, they evolve through cycles of data condensation, interpretation, narrative synthesis, and recontextualization. This process mirrors the recursive logic of narrative inquiry itself—moving back and forth between data and meaning until a coherent yet reflexively transparent story form emerges. Iteration here is not merely procedural but interpretive, foregrounding the researcher’s reflexivity in shaping representation.

5 Full integration

Finally, the fifth element—full integration—achieves its most concrete expression in narrative profiling. Importantly, the concept of *full integration* should not be interpreted as implying a fixed, absolute, or objectively verifiable endpoint of analytic completeness. Rather, within the present framework, full integration is understood as a representational ideal—an interpretive state in which quantitative and qualitative components/phases/strands are brought into coherent, meaning-centered alignment within a unified narrative form. As such, the degree of integration achieved is necessarily contingent, situated, and reflexively constructed, depending on the analytic decisions, data sources, and interpretive aims of the researcher. This perspective aligns with the broader argument advanced in this article that integration is not a final destination but an ongoing, iterative, and narrative accomplishment.

Instead of positioning the proposed typology and its underlying analytic worldview as representing the “highest potential” of qualitizing, it is more appropriate to view this framework as one possible—albeit substantively elaborated—instantiation of qualitizing’s evolving methodological and epistemological possibilities. Within the pluralistic orientation of CDP 2.0 (Onwuegbuzie and Abrams, 2025), qualitizing is not understood as progressing toward a single, fixed endpoint, but as an open, generative, and continually expanding domain of meaning-centered integration. In this sense, the present taxonomy is intended to illustrate how narrative profiling can facilitate highly integrative and conceptually rich forms of qualitizing while remaining one of multiple viable pathways through which such integration can be achieved. Accordingly, the contribution of this framework lies not in establishing a definitive or maximal form of qualitizing but in advancing a comprehensive and extensible model that invites further refinement, application, and theoretical development.

Taken together, these reinterpretations re-situate the five elements of qualitizing within a narrative epistemology. To elucidate further the interpretive architecture underlying qualitizing, it is useful to conceptualize narrative profiling as operating across multiple, interrelated layers of reasoning. These layers—descriptive, analytic, contextual, relational, and reflexive—are not introduced here as a formally derived or exhaustive framework but rather as a heuristic synthesis that emerges from the integration of the five elements of qualitizing (Onwuegbuzie and Leech, 2019, 2021), the narrative turn in mixed methods research, and the pluralistic, reflexive commitments of CDP 2.0 (Onwuegbuzie and Abrams, 2025). In this sense, they represent overlapping dimensions of interpretive engagement through which data are transformed into meaning rather than discrete or sequential stages of analysis.

Within this interpretive framing, descriptive reasoning corresponds to the representation of central tendencies and patterns (e.g., modal or average profiles), analytic reasoning involves identifying relationships, contrasts, or structures across data (e.g., comparative or cluster-based profiles), contextual reasoning situates findings within broader environmental, cultural, or temporal settings (e.g., contextual or cultural profiles), relational reasoning emphasizes connections among individuals, systems, or processes (e.g., relational or interactional profiles), and reflexive reasoning foregrounds the role of values, positionality, and ethical interpretation (e.g., critical or transformative profiles). Importantly, these layers should be understood as dynamically interwoven rather than as independently operating components. Although Table 1 illustrates the multidimensional and integrative nature of qualitizing, it is not intended to depict these layers explicitly; rather, the layers provide a complementary interpretive lens through which the table —and the broader narrative profiling framework—can be more fully understood.

**Table 1:** Conceptual grouping of the 43 narrative profile types according to meta-categorical analytic and methodological orientations within qualitizing.

**Group / category**	**Narrative profile types**	**Illustrative orientation / key focus**
1. Foundational profiles	Modal, average, holistic, comparative, normative	Core narrative foundations; representativeness and comparison
2. Exemplar and pattern-based profiles	Exemplar, cluster, aggregated, latent, behavioral, predictive, causal, thematic, sentiment	Pattern discovery and typological synthesis
3. Temporal and developmental profiles	Dynamic, trajectory, evolutionary, developmental, historical, event-centric, narrative arc, transformative	Temporal change, growth, and transformation
4. Contextual and environmental profiles	Contextual, comparative contextual, context-sensitive, geographic, spatial	Environmental and situational influence
5. Cultural, identity, and social positioning profiles	Cultural, intersectional, marginalized, identity, persona	Cultural framing, self-construction, and positionality
6. Meaning, value, and ethical profiles	Value-based, ethical or moral, aspirational, critical	Value systems, ethical reasoning, and transformative meaning
7. Relational and interactional profiles	Relational, interactional, conflict, network, decision-making	Interpersonal dynamics, collaboration, and discourse
8. Experiential and reflective profiles	Experiential, resilience, holistic (also cross-listed)	Lived experience, introspection, and adaptive meaning-making
9. Analytic and structural profiles *(cross-cutting group)*	Aggregated, cluster, latent, network, causal	Methodological structuring and integrative modeling

Certain profiles (e.g., Holistic, Transformative, Sentiment) appear in more than one group due to their integrative analytic functions.

Narrative profiling demonstrates that qualitizing is most powerful when it not only converts data forms, but also transforms representational logic—shifting from numerical or categorical abstraction toward narrative embodiment. This reframing serves as a conceptual bridge between the foundational work of Onwuegbuzie and Leech (2019, 2021) and the expanded taxonomy developed in the present article, positioning narrative profiling as both a continuation and an evolution of the qualitizing enterprise.

## The narrative turn in qualitizing

The reinterpretation of qualitizing through narrative profiling coincides with, and contributes to, what may be described as the *narrative turn in mixed methods research*. This turn reflects a broader epistemological shift across the social, behavioral, and health sciences—including the medical and clinical fields—from viewing data as static indicators of reality to understanding them as *constructed representations of meaning*. Within this orientation, the act of research itself becomes a process of storying: selecting, sequencing, and contextualizing information to make human experience intelligible. Thus, qualitizing is not simply a transformation of data form but an evolution in representational consciousness, situating integration within the realm of interpretation and meaning-making.

The narrative turn reframes the purpose of integration. Historically, mixed methods researchers have sought integration primarily as a *technical* goal—combining numerical precision with interpretive depth to achieve fuller understanding. However, narrative integration transcends the procedural level. It operates as an *epistemic synthesis*, wherein the coherence of mixed methods research arises from the narrative logic that binds data, interpretation, and context into a unified account. Through narrative profiling, integration is achieved not only by aligning datasets, but also by aligning *meaning systems*—the researcher’s interpretive narrative, the participants’ storied experiences, and the patterns revealed through quantitative analysis.

This shift is grounded in the long intellectual lineage of the narrative turn in the human sciences. Scholars such as Bruner (1990, 2009), Polkinghorne (1995), and Riessman (2008) have long argued that human understanding is fundamentally narrative in nature: We come to know through story. Applied to mixed methods research, this view repositions qualitizing as an act of narrative knowing—wherein numbers are not merely translated into words, but *re-voiced* within storied frames that infuse temporal, emotional, and cultural context. In this way, the narrative turn deepens the interpretive dimension of mixed methods integration by reasserting that knowledge is not found within data alone but within the *narrative relationships* that give data meaning.

Moreover, narrative profiling redefines the role of the researcher. Within the narrative turn, the researcher is not a neutral translator between quantitative and qualitative realms but an *authorial integrator* who constructs coherence through reflexive narration. Each narrative profile represents a crafted act of integration in which interpretation is both disciplined and creative. This repositioning does not erode methodological rigor; rather, it relocates rigor in transparency, reflexivity, and the coherence of the interpretive logic that binds the profile together.

Ultimately, the narrative turn in qualitizing extends the field’s conceptual boundaries by joining the methodological ambitions of mixed methods research with the interpretive ethos of narrative inquiry. It advances an understanding of integration as a narrative accomplishment: one that unites data forms, analytical logics, and representational voices into a cohesive expression of meaning. In so doing, it situates qualitizing at the heart of the interpretive transformation of mixed methods research—an approach that recognizes that the most powerful integrations are not merely *mechanical convergences of data* but *narrative convergences of meaning*.

Importantly, the use of the term *meaning* rather than *understanding* is intentional and reflects the pluralistic epistemological stance that underpins this article. It is essential to distinguish narrative profiling from the common practice in qualitative research of illustrating findings through participant quotations or data excerpts. Although the inclusion of verbatim quotations is widely regarded as a hallmark of rigor and transparency in qualitative reporting, such practices typically function as evidentiary support for themes rather than as integrative analytic representations in their own right. As noted by Bazeley (2009), reliance on isolated excerpts can result in what she characterizes as *superficial reporting*, wherein data are presented descriptively without sufficient synthesis or interpretive integration. In contrast, narrative profiling does not merely display data but transforms it into coherent, structured, and analytically grounded representations that integrate patterns across cases, variables, or data sources. Thus, whereas quotations and excerpts serve to exemplify analytic claims, narrative profiles function as the analytic product itself—embodying the synthesis of evidence, interpretation, and meaning within a unified narrative form.

Importantly, this distinction should not be interpreted as a rejection of participant quotations or qualitative excerpts. Rather, narrative profiling can incorporate such materials as embedded elements within a broader integrative structure. In this way, excerpts serve as supporting evidence within a narrative profile, while the profile itself provides the higher-order synthesis that situates these data within a coherent interpretive framework. Consequently, narrative profiling extends—rather than replaces—conventional qualitative reporting practices by moving from illustrative description toward integrative representation.

The appropriateness and value of narrative profiling also are contingent upon the purpose of the research and the methodological orientation guiding the inquiry. Narrative profiling is particularly well-suited to studies that seek to achieve integration, synthesis, or meaning-centered interpretation across data sources, cases, or analytic dimensions—such as in mixed methods research or in qualitative research approaches emphasizing interpretation, explanation, or theory building. However, not all qualitative inquiries aim to move beyond description. For example, methodologies such as qualitative descriptive analysis or certain forms of content analysis prioritize faithful representation of categories or frequencies over interpretive synthesis. In such contexts, the use of narrative profiling may be neither necessary nor appropriate. Accordingly, the call to move *beyond* description should be understood not as a universal imperative, but as a methodological option aligned with specific research aims, questions, and epistemological commitments.

In this light, narrative profiling is best conceptualized not as a superior or obligatory mode of qualitative reporting, but as one of several possible analytic strategies available to researchers. Its contribution lies in its capacity to facilitate deeper integration and meaning-making when such goals are aligned with the study’s purpose. Thus, the decision to employ narrative profiling should be guided by the research question, the nature of the data, and the intended level of analytic interpretation, rather than by any prescriptive hierarchy of methodological approaches.

Within the framework of CDP 2.0 (Onwuegbuzie and Abrams, 2025), meaning is not assumed to be singular, fixed, or uniformly understood across readers or contexts. Rather, meaning is co-constructed, contextually situated, and open to multiple interpretations that can coexist without requiring convergence toward a single, unified understanding. In this sense, narrative integration is best conceived not as producing definitive understanding but as facilitating rich, layered, and dialogical engagements with data that invite diverse interpretive possibilities.

Narrative profiles mark the interpretive culmination of the qualitizing process. By converting data into story, they preserve precision while generating depth. The 43 narrative profile types presented here represent both a classification system and a methodological tool, offering structured ways to integrate data forms, analytic modes, and interpretive purposes.

The narrative turn in qualitizing thus establishes the philosophical foundation for the methodological expansion that follows. If narrative represents the *epistemic language* through which integration achieves meaning, then narrative profiling provides the *grammar*—the structured, typological system through which diverse forms of narrative integration can be realized. Building on the five foundational profiles identified by Tashakkori and Teddlie (1998), I advance this representational logic into a broader, more differentiated taxonomy of 43 narrative profile types. This expansion operationalizes the principles of the narrative turn by demonstrating how varied narrative forms—ranging from temporal and cultural to ethical and transformative—can function as distinct modes of data integration, each translating complex patterns of evidence into coherent and human-centered meaning.

## The expansion: the 38 additional narrative profile types

The expansion from 5 to 43 narrative profile types arises from both methodological necessity and conceptual maturation. Although the foundational profiles articulated by Tashakkori and Teddlie (1998)—modal, average, holistic, comparative, and normative—provided a pioneering framework for transforming quantitative data into qualitative meaning, they represented only the initial manifestations of a far broader representational potential. As the mixed methods research field has evolved, the scope of qualitizing has expanded beyond describing “typicality” or “comparison” to include dynamic processes, ethical stances, temporal sequences, and cultural contexts through which meaning is constituted. The present taxonomy extends the original typology to capture this expanded ontology of integration: one in which narrative profiling is not limited to summarizing data, but is capable of expressing transformation, reflexivity, and value orientation. Each additional profile type thus represents a new *mode of narrative reasoning*—a distinct way of embodying the integration of inference and interpretation—thereby positioning narrative profiling as a comprehensive yet evolving, pluralistic framework for meaning-centered data transformation across the social, behavioral, and health sciences. It should be noted that although extensive, the 43 narrative profile types are not presented as an exhaustive or final enumeration; rather, they represent the principal modes of narrative integration currently identifiable within qualitizing.

To maintain epistemological coherence with the pluralistic orientation of this article, it is important to emphasize that the typology is not intended to represent a definitive or exhaustive mapping of all possible narrative profile forms. Rather, it should be understood as an evolving and generative framework that captures a broad and conceptually grounded range of narrative integration strategies identifiable at the current stage of methodological development. Consistent with CDP 2.0 (Onwuegbuzie and Abrams, 2025), the framework remains intentionally open to extension, refinement, and the incorporation of emergent forms as new epistemological perspectives and analytic innovations continue to shape the field.

Importantly, the characterization of this typology as comprehensive should be understood in a relative and developmental sense rather than as implying exhaustiveness or finality. The present framework seeks to capture the most salient and conceptually distinct forms of narrative profiling currently identifiable within the evolving landscape of mixed methods research. At the same time, consistent with the pluralistic and generative orientation of this work, the typology remains intentionally open to refinement, extension, and the emergence of new profile types as methodological innovation and interdisciplinary application continue to expand the boundaries of qualitizing practice.

### Extended typology of the 43 narrative profile types

Building on this conceptual expansion, Table 2 presents the typology of 43 distinct narrative profiles as a comprehensive, integrative map of the representational possibilities that emerge when quantitative and qualitative logics are synthesized through narrative form. Whereas the five foundational profiles identified by Tashakkori and Teddlie (1998)—modal, average, holistic, comparative, and normative—offered the first formal articulation of qualitizing as narrative translation, the expanded taxonomy extends their pioneering work into a broader meta-framework that captures a wide and evolving range of analytic, interpretive, and contextual variation evident in contemporary mixed methods research.

**Table 2:** Comprehensive typology of 43 narrative profile types: definitions, analytic foci, operations, outputs, and applications.

**Narrative profile type**	**Definition**	**Analytic focus / purpose**	**Core analytic operations**	**Potential outputs / outcomes**	**Example of application**
Modal	Represents the most frequently occurring pattern or participant characteristics within a dataset.	Descriptive representativeness	Frequency analysis; modal value translation	Typical participant profile; representative case summary	Identifying the “typical” teacher’s motivational pattern
Average	Synthesizes mean or central tendency data into a composite narrative portrait.	Central tendency; group-level summarization	Mean computation; aggregation; narrative synthesis	“Average person” narrative; typical trend summary	Depicting the “average” respondent’s resilience level
Holistic	Integrates multiple dimensions of data into a cohesive narrative of one case or phenomenon.	Multidimensional integration	Cross-domain synthesis; matrix coding	Whole-person or system narrative	Depicting a participant’s integrated adjustment experience
Comparative	Contrasts two or more groups to highlight differences and similarities.	Between-group contrast	Cross-case comparison; mixed data juxtaposition	Contrastive narratives	Comparing high vs. low-performing schools
Normative	Positions data relative to a benchmark or standard.	Evaluation against norms	z score/percentile transformation	Norm-referenced case narrative	Patient’s well-being relative to population norms
Exemplar	Highlights a single case as illustrative of a broader pattern or principle.	Case illustration	Purposeful selection; narrative elaboration	Exemplar case description	A prototypical patient narrative
Cluster	Groups similar cases based on quantitative and/or qualitative attributes.	Typological categorization	Cluster analysis; thematic grouping	Typology of participant types	Identifying motivational subgroups
Aggregated	Highlights specific cases or individuals who exemplify a particular characteristic or behavior within the group being studied; useful for illustrating extreme or particularly illustrative examples within the data set.	Cross-level integration	Data merging; cross-tab synthesis	Aggregate-level insight	Combining survey and interview data
Latent	Derived from hidden patterns or underlying variables within data, this profile focuses on factors not immediately apparent but significant in shaping the narrative (e.g., unconscious biases or implicit attitudes).	Hidden structures	Factor or latent class analysis	Latent narrative construct	Interpreting latent resilience profiles
Behavioral	Focused on patterns of action or decision-making within the unit of analysis, offering insights into behavioral tendencies or routines.	Behavioral expression of constructs	Coding of behavioral data; pattern linking	Behavior-based narrative	Linking observed classroom actions to motivation
Predictive	Leveraging existing data to construct profiles that forecast future behaviors, decisions, or outcomes, often integrating qualitative interpretations with statistical trends.	Forecasting and inference	Regression; predictive modeling	Anticipatory narrative	Projecting future student engagement patterns
Dynamic	Capture changes over time in an individual’s or group’s attitudes, behaviors, or experiences; Particularly useful in longitudinal studies wherein the goal is to track development or shifts in perceptions or actions.	Temporal change	Time-series or longitudinal modeling	Temporal storyline	Depicting motivation trajectories over semesters
Trajectory	Similar to dynamic profiles but with a specific focus on the pathway or sequence of events leading to an outcome; often used in process-oriented studies like life histories or career trajectories.	Developmental progression	Growth modeling; repeated measures	Growth narrative	Tracking identity development in adolescents
Evolutionary	Focused on tracing the development or transformation of a phenomenon over extended periods, this profile emphasizes slow or generational changes in attributes or behaviors.	Transformational sequence	Sequential qualitative analysis	Evolution narrative	Evolution of organizational culture
Developmental	Highlights stages of growth or change within individuals or groups, focusing on progression or regression across a timeline, often in education, psychology, or child development research.	Individual maturation	Stage analysis; mixed synthesis	Developmental profile	Tracking leadership skill growth
Contextual	Integrates the influence of situational or environmental factors on individuals or groups; Emphasizes how context shapes behaviors, attitudes, or outcomes and particularly is useful in ecological or contextual analyses.	Contextual influence	Context mapping; matrix coding	Context-rich narrative	Understanding teacher motivation within policy context
Comparative contextual	A hybrid of comparative and contextual profiles, focusing on comparing units of analysis across different environmental or situational contexts to highlight the role of external influences.	Context-by-context comparison	Cross-case contextual mapping	Contextual contrasts	Comparing rural vs. urban schools
Context-sensitive	Takes into account variability in individual or group responses based on shifting contexts or environments, emphasizing adaptability or situational dependence.	Interpretive adaptation	Iterative coding; adaptive interpretation	Adaptive narrative	Interpreting data differently by setting
Geographic	Integrates spatial data to construct narratives that emphasize location-based differences or patterns, often useful in geographic or environmental studies.	Spatial distribution	GIS/spatial analysis	Spatial narrative map	Mapping public health disparities
Spatial	Integrating geographical or locational data into narrative descriptions, this profile explores the role of physical space or environment in shaping the unit of analysis.	Relational positioning	Spatial metric modeling	Spatial pattern narrative	Analyzing spatial inequality in cities
Cultural	Highlighting cultural influences and shared norms within a group, this profile emphasizes the role of cultural identity in shaping behaviors, attitudes, or outcomes.	Cultural interpretation	Cross-cultural coding; ethnographic synthesis	Cultural narrative	Teachers’ professional identities across cultures
Intersectional	Analyzes the interaction between multiple identity factors (e.g., race, gender, socioeconomic status) and how these intersections influence experiences or outcomes.	Intersecting identity analysis	Intersectional coding; matrix analysis	Intersectional narrative	Women of color in academia narratives
Marginalized	Highlighting the experiences of individuals or groups at the margins of societal norms or systems, this profile focuses on voices often underrepresented in traditional analyses.	Equity and inclusion	Critical analysis; narrative reconstruction	Counter-narrative	Narratives of marginalized youth
Identity	Constructed to explore how personal or collective identities are formed, expressed, and negotiated within the group of interest.	Self and identity construction	Discourse or thematic analysis	Identity narrative	How first-year teachers form professional identity
Persona	Often used in user research and human-centered design to represent archetypical users based on shared goals, behaviors, and attitudes. It synthesizes qualitative and quantitative data into fictional yet representative profiles.	User or participant archetype	Cross-case synthesis	Persona profiles	Designing educational interventions via personas
Value-based	Explores the core values, beliefs, or principles guiding individuals or groups, often constructed from qualitative data like interviews or focus groups.	Values and ethics	Value coding; interpretive synthesis	Value-centered narrative	Decision-making informed by moral principles
Ethical or moral	Explore ethical frameworks, decision-making processes, or moral reasoning within the unit of analysis, often constructed from qualitative insights in bioethics or moral psychology.	Ethics and moral judgment and sensemaking	Ethical narrative analysis; narrative moral analysis	Ethical reflection profile; moral reasoning narrative	Moral decision-making in health care; students’ ethical identity formation
Aspirational	Focuses on the goals, hopes, or aspirations of individuals or groups, emphasizing potential or desired outcomes.	Motivation and goal pursuit	Future-oriented coding	Aspirational narrative	Students’ visions of career success
Causal	Explicitly constructed to investigate cause-and-effect relationships within the data, focusing on how specific variables or interventions impact individuals or groups.	Causation and mechanism	Path modeling; inferential linkage	Causal narrative	Explaining intervention effects
Critical	Focused on deconstructing power dynamics, ideologies, or systemic structures affecting the unit of analysis. This profile is rooted in critical theory and aims to uncover hidden assumptions or societal influences.	Power, discourse, and ideology	Critical discourse analysis	Counter-hegemonic narrative	Deconstructing neoliberal education rhetoric
Transformative	Highlights the impact of interventions or changes on individuals or groups, documenting shifts in perspectives, behaviors, or outcomes. It is particularly relevant in evaluative research or action-oriented studies.	Social change and empowerment	Mixed transformative analysis	Change-oriented narrative	Community transformation through research
Relational	Focuses on relationships and interactions between individuals or groups, emphasizing dynamics like power, collaboration, or conflict. Useful in studies of social networks or organizational behavior.	Networked meaning	Network analysis; co-occurrence mapping	Relationship-based narrative	Mapping teacher collaboration networks
Interactional	Emphasizes the dynamics and relationships between individuals or groups, highlighting interactions and their effects on collective outcomes or perceptions.	Interactional meaning	Conversation or discourse analysis	Dialogic narrative	Doctor–patient interaction stories
Conflict	Designed to explore tensions, disagreements, or conflicts within or between groups, this profile focuses on understanding sources, dynamics, and resolutions of conflict.	Conflict and resolution	Narrative tension analysis	Conflict-resolution narrative	Workplace or policy conflict stories
Network	Constructed by mapping connections and relationships within a group, this profile focuses on social or organizational networks and their influence on behavior or information flow.	Structural relationships	Social network analysis	Network-based narrative visualization	Mapping interagency collaboration
Decision-Making	Exploring how individuals or groups arrive at decisions, this profile emphasizes cognitive processes, contextual factors, and outcomes.	Judgment and choice	Decision modeling + interpretive synthesis	Decision narrative	Understanding patient treatment decisions
Experiential	Focuses on detailed accounts of lived experiences, highlighting the subjective perspectives of individuals or groups. This profile often is used in phenomenological studies.	Phenomenological understanding	Thematic phenomenological analysis	Lived-experience narrative	Chronic illness experiences
Resilience	Focuses on coping mechanisms, adaptive strategies, and recovery pathways for individuals or groups facing challenges or adversity.	Strength and adaptation	Mixed resilience modeling; thematic synthesis	Resilience narrative	Recovery stories post-disaster
Historical	Constructed using historical data to explore how past events or trends influence current behaviors or outcomes. This profile is valuable in historical or sociological research.	Historical process	Archival/narrative reconstruction	Historical storyline	Institutional change histories
Event-Centric	Constructed around specific events or phenomena, this profile analyzes how a significant occurrence impacts individuals or groups, offering a detailed narrative of its ripple effects.	Event-based meaning	Critical incident analysis	Event narrative	Trauma or crisis narratives
Narrative Arc	Constructed to represent the “storyline” of a group or individual, this profile incorporates beginning, middle, and end phases, emphasizing the sequence and resolution of key events or processes.	Narrative coherence	Structural narrative analysis	Story-structure representation	Plot development in identity narratives
Thematic	Derived from recurring themes in the data, this profile focuses on capturing the essence of qualitative findings centered on thematic groupings rather than on individual or aggregate characteristics.	Cross-cutting patterns	Thematic content analysis	Thematic synthesis	Common themes across participants
Sentiment	Built from emotional or attitudinal data, this profile provides a nuanced look at the affective dimensions of individuals or groups, often using qualitative descriptions derived from sentiment analysis.	Emotional or attitudinal dimensions	Sentiment analysis; affective coding	Affective narratives; tone summaries	Emotional responses to organizational change

Importantly, the designation of the nine profile families as *hierarchical* should not be interpreted as implying a rigid, linear, or strictly nested ordering of profile types. Rather, the taxonomy is more accurately understood as a multidimensional and layered architecture in which certain profiles—particularly the foundational types—provide conceptual grounding, while subsequent families represent progressively specialized, contextually embedded, and analytically elaborated forms of narrative integration. In this sense, the hierarchy is not one of rank or exclusivity, but of conceptual scaffolding and developmental extension. At the same time, consistent with the pluralistic orientation of this framework, the profile families operate across intersecting epistemic dimensions, reflecting a system in which multiple forms of narrative reasoning coexist, interact, and can be combined in flexible and non-linear ways. Thus, the taxonomy is best understood as both structured and multidimensional—simultaneously organized and open, layered yet integrative.

Table 2 functions not merely as a classificatory table, but as a conceptual atlas of qualitizing practice. Each profile type corresponds to a distinct epistemic purpose, reflecting different ways in which narrative profiling can be used to generate, to organize, and to interpret meaning within mixed methods research. In this sense, the typology visualized in Table 2 is both *methodological* and *epistemological*: methodological because it delineates concrete analytic procedures that guide how different kinds of data can be narratively integrated, and epistemological because it embodies the underlying logics of meaning-making that define the narrative turn in mixed methods research.

To aid interpretive clarity, Table 2 is organized across several key columns. The *Narrative Profile Type* column names each representational form, beginning with the five foundational types and extending through additional categories derived from iterative theoretical synthesis and cross-disciplinary exemplars. The “Concise Definition” column provides brief but conceptually rich descriptions of each profile, allowing readers to grasp its essential logic without needing extended textual elaboration. The “Analytic Focus / Purpose” column identifies the primary epistemic aim of each profile—whether descriptive, comparative, evaluative, developmental, or transformative. The “Core Analytic Operations” column indicates the analytic procedures typically associated with each profile, linking representational forms to corresponding methods (e.g., cluster analysis, thematic synthesis, network modeling, or narrative interpretation). The “Potential Outputs / Outcomes” column summarizes what each profile produces as an integrative artifact—such as a composite narrative, typology, developmental storyline, or interpretive model. Finally, the *Example of Application* column grounds each profile in a brief, discipline-relevant illustration, demonstrating how it may be operationalized in psychological, educational, health, or social research contexts.

Together, these columns enable the table to function as both a reference taxonomy and a heuristic tool. Researchers can use it to identify the narrative profile most suited to their analytic aims or to design mixed methods research studies that intentionally integrate quantitative precision with qualitative depth through narrative logic. Importantly, the typology is not prescriptive: It is designed to be pluralistic, allowing scholars to combine or adapt profile types as warranted by their epistemological stance, research question, and dataset configuration. By presenting the 43 types side by side, Table 2 makes visible the continuum of qualitizing—from foundational descriptive forms to advanced integrative and transformative profiles—thereby illustrating the methodological elasticity and representational richness that narrative profiling introduces to mixed methods inquiry.

## Guidance for selecting narrative profile types

Given the breadth and multidimensionality of the 43-profile taxonomy, it is neither feasible nor desirable within a single article to provide fully elaborated procedural guidance for each individual profile type. Accordingly, a follow-up methodological article is planned that will offer detailed, profile-specific guidance, including procedural steps, decision criteria, and applied exemplars for a broad and evolving range of narrative profile types. At the same time, it is important to support researchers in making informed decisions about when and how to apply different narrative profiles in practice. Thus, the following discussion provides a set of guiding principles that can assist researchers in selecting among profile types based on their analytic purposes, data characteristics, and interpretive goals.

In general, the selection of narrative profile types can be guided by three overarching considerations. First, the analytic purpose of the study should serve as the primary driver. For example, descriptive and representative aims align most closely with foundational profiles (e.g., modal, average, or holistic), whereas pattern detection and typological synthesis align with exemplar and pattern-based profiles (e.g., cluster, latent, or thematic). Second, the structure and source of the data should inform profile selection. Quantitatively derived datasets lend themselves to profiles such as predictive, causal, or trajectory, whereas qualitatively rich datasets are well suited to experiential, identity, or narrative arc profiles. Third, the level of integration sought—whether within-case, cross-case, or cross-community—should guide the choice of profile, with more complex integrative aims aligning with contextual, relational, or meta-narrative profiles. Taken together, these considerations position the taxonomy not as a fixed classification system but as a flexible analytic toolkit adaptable to diverse research contexts.

To support applied decision-making further, these considerations can be operationalized heuristically. For instance, when the primary goal is to translate statistical summaries into accessible meaning, researchers might prioritize modal or average profiles; when the goal is to illuminate variability or subgroup differentiation, cluster, latent, or comparative profiles are likely to be more appropriate. Studies focused on change over time or processual dynamics would benefit from trajectory, developmental, or narrative arc profiles, whereas research emphasizing context, positionality, or lived experience might be better served by contextual, identity, or cultural profiles. Additionally, when the aim is to achieve higher-order integration across cases or communities, researchers might employ relational, meta-narrative, or cross-community synthesis profiles. Importantly, these decisions are not mutually exclusive; rather, researchers can combine and sequence multiple profile types within a single study to reflect the iterative and multidimensional nature of qualitizing. In this way, the taxonomy supports not only selection, but also the intentional design of integrated analytic pathways.

These guiding principles are not intended to be prescriptive but rather heuristic. The strength of the 43-profile taxonomy lies in its flexibility and extensibility, allowing researchers to adapt, to combine, and to iterate across profile types in alignment with their epistemological stance and research questions. In this way, narrative profiling becomes not only a set of techniques, but also a mode of analytic reasoning that supports the transformation of data into meaning-centered, integrated representations.

In sum, Table 2 encapsulates the evolution of qualitizing as both a technique and a philosophy of integration. It concretizes the narrative turn by translating abstract principles of meaning-making into a structured yet flexible repertoire of analytic possibilities. Each profile type represents a unique narrative pathway through which data may be transformed, integrated, and humanized. Collectively, they reveal that qualitizing is not confined to a single representational act but, instead, is an evolving, multidimensional framework for rendering complexity intelligible through story.

### Mapping the 43 narrative profile types to the five elements of qualitizing

Whereas Table 2 introduces the 43 narrative profile types as distinct modes of narrative reasoning, Table 3 advances the discussion by showing how these profiles align with the five major elements of qualitizing formulated by Onwuegbuzie and Leech (2019, 2021): multiple representations, data source flexibility, analytic flexibility, analytic multiplicity, and full integration. In this context, the designation “mixed” in the *Data Source Flexibility* column indicates that a profile type can accommodate the integration of both quantitative and qualitative data, but does not imply that both forms are required; rather, many profiles can be generated from quantitative data alone, qualitative data alone, or their combination, depending on the analytic purpose and design. This mapping reveals that the expansion from 5 to 43 profiles is not merely additive but *systemic*: The new profiles collectively instantiate the full qualitizing logic across diverse analytic orientations.

**Table 3:** Conceptual mapping of the 43 narrative profile types to the five major elements of qualitizing and their analytic orientations.

**Narrative profile type**	**Numerous representations**	**Data source flexibility**	**Analytic flexibility**	**Analysis type(s)**	**Analytic multiplicity**	**Full integration (1 + 1 = 1)**
Modal	✓	✓ (Quantitative)	✓ (Descriptive/inferential integration)	QUAN → QUAL	✓	✓
Average	✓	✓ (Quantitative)	✓ (Descriptive/inferential integration)	QUAN → QUAL	✓	✓
Holistic	✓	✓ (Qualitative)	✓ (Interpretive synthesis)	QUAL	✓	✓
Comparative	✓	✓ (Mixed)	✓ (Cross-case comparative)	MIXED (QUAL + QUAN)	✓	✓
Normative	✓	✓ (Quantitative)	✓ (Benchmark comparison)	QUAN	✓	✓
Exemplar	✓	✓ (Qualitative)	✓ (Case-based interpretation)	QUAL	✓	✓
Cluster	✓	✓ (Quantitative)	✓ (Cluster analytic modeling)	QUAN	✓	✓
Aggregated	✓	✓ (Mixed)	✓ (Data integration synthesis)	MIXED	✓	✓
Latent	✓	✓ (Quantitative)	✓ (Latent variable/factor modeling)	QUAN	✓	✓
Behavioral	✓	✓ (Mixed)	✓ (Behavioral coding/statistical frequency)	MIXED	✓	✓
Predictive	✓	✓ (Quantitative)	✓ (Regression/predictive modeling)	QUAN	✓	✓
Dynamic	✓	✓ (Mixed longitudinal)	✓ (Sequential time-based modeling)	QUAN → QUAL	✓	✓
Trajectory	✓	✓ (Quantitative)	✓ (Growth curve/sequential modeling)	QUAN → QUAL	✓	✓
Evolutionary	✓	✓ (Mixed)	✓ (Sequential developmental interpretation)	QUAL → QUAN	✓	✓
Developmental	✓	✓ (Mixed)	✓ (Sequential developmental synthesis)	QUAL → QUAN	✓	✓
Contextual	✓	✓ (Mixed)	✓ (Contextual pattern analysis)	QUAL + QUAN	✓	✓
Comparative contextual	✓	✓ (Mixed)	✓ (Contextual cross-case comparison)	MIXED	✓	✓
Context-Sensitive	✓	✓ (Qualitative)	✓ (Interpretive adaptation analysis)	QUAL	✓	✓
Geographic	✓	✓ (Quantitative)	✓ (Spatial analysis/GIS)	QUAN	✓	✓
Spatial	✓	✓ (Quantitative)	✓ (Spatial metric modeling)	QUAN	✓	✓
Cultural	✓	✓ (Mixed)	✓ (Interpretive cultural analysis)	MIXED	✓	✓
Intersectional	✓	✓ (Qualitative)	✓ (Intersectional interpretation)	QUAL	✓	✓
Marginalized	✓	✓ (Qualitative)	✓ (Critical narrative)	QUAL	✓	✓
Identity	✓	✓ (Qualitative)	✓ (Identity construction analysis)	QUAL	✓	✓
Persona	✓	✓ (Mixed)	✓ (User archetype modeling)	MIXED	✓	✓
Value-based	✓	✓ (Mixed)	✓ (Interpretive moral/value analysis)	QUAL → QUAN	✓	✓
Ethical or moral	✓	✓ (Qualitative)	✓ (Interpretive ethical or moral narrative reasoning)	QUAL	✓	✓
Aspirational	✓	✓ (Qualitative)	✓ (Goal-oriented interpretation)	QUAL	✓	✓
Causal	✓	✓ (Mixed)	✓ (Causal modeling/inference)	QUAN → QUAL	✓	✓
Critical	✓	✓ (Qualitative)	✓ (Interpretive/critical discourse)	QUAL	✓	✓
Transformative	✓	✓ (Mixed)	✓ (Mixed transformative sequence)	MIXED (QUAL + QUAN)	✓	✓
Relational	✓	✓ (Mixed)	✓ (Social network or thematic mapping)	MIXED	✓	✓
Interactional	✓	✓ (Qualitative)	✓ (Conversational/interpersonal analysis)	QUAL	✓	✓
Conflict	✓	✓ (Qualitative)	✓ (Conflict narrative analysis)	QUAL	✓	✓
Network	✓	✓ (Quantitative)	✓ (Network metrics analysis)	QUAN	✓	✓
Decision-making	✓	✓ (Mixed)	✓ (Decision model + narrative synthesis)	MIXED	✓	✓
Experiential	✓	✓ (Qualitative)	✓ (Phenomenological analysis)	QUAL	✓	✓
Resilience	✓	✓ (Mixed)	✓ (Scale integration + thematic interpretation)	MIXED (QUAN + QUAL)	✓	✓
Historical	✓	✓ (Qualitative)	✓ (Chronological narrative analysis)	QUAL	✓	✓
Event-centric	✓	✓ (Qualitative)	✓ (Event-based interpretive analysis)	QUAL	✓	✓
Narrative arc	✓	✓ (Qualitative)	✓ (Story-structure analysis)	QUAL	✓	✓
Thematic	✓	✓ (Mixed)	✓ (Thematic content analysis)	MIXED	✓	✓
Sentiment	✓	✓ (Mixed)	✓ (Sentiment and affective analysis)	MIXED	✓	✓

Table 3 functions as a *meta-framework of correspondence*. It positions each narrative profile within the larger architecture of qualitizing, demonstrating that narrative profiling operates simultaneously at several analytic levels—representational, procedural, and integrative. In so doing, this table highlights the multidimensional coherence of the expanded taxonomy: Each profile type embodies one or more of the five elements to varying degrees, depending on its analytic orientation and methodological lineage.

Table 3’s columns serve interpretive functions that clarify these relationships.

- The “Numerous Representations” column identifies which profiles generate multiple narrative forms or outputs, illustrating the first element of qualitizing—its capacity for plural representation.
- “Data Source Flexibility” indicates the range of data inputs (quantitative, qualitative, or mixed methods) that can give rise to each profile, thereby reflecting the second element.
- “Analytic Flexibility” corresponds to the third element, showing how each profile can accommodate different modes of analysis—descriptive, inferential, interpretive, or cross-case. It is important to clarify that the categorization of narrative profile types in terms of analytic flexibility does not represent a direct mapping onto the third element of qualitizing (i.e., the transformation and integration of quantitative and qualitative data) but rather reflects an indirect manifestation of this element. Specifically, analytic flexibility captures the range of analytic modes—such as descriptive, inferential, interpretive, or cross-case—that become possible as a result of integrating diverse forms of data within narrative profiling. In this sense, the third element pertains to the *sources and forms of data being integrated*, whereas analytic flexibility pertains to the *variety of analytic operations that such integration enables*. Accordingly, the categories presented in Table 3 should be understood as heuristic and illustrative rather than exhaustive or mutually exclusive classifications, intended to highlight dominant analytic tendencies rather than to impose rigid boundaries across profile types.
- The “Analysis Type(s)” column captures the typical analytic sequence (e.g., QUAN → QUAL, QUAL → QUAN, or MIXED), directly connecting the profiles to mixed methods integration logic.
- “Analytic Multiplicity” addresses the fourth element, indicating profiles that operate through iterative or multi-stage interpretation.
- Finally, “Full Integration (1 + 1 = 1)” visualizes the fifth and culminating element: the unification of quantitative and qualitative reasoning into a single coherent interpretive product.

Viewed together, these columns make Table 3 a *conceptual synthesis* linking micro-level analytic processes to the macro-level logic of integration. For example, the mapping shows that profiles such as *modal* and *average* primarily engage in descriptive integration of quantitative findings, whereas profiles such as *holistic* and *transformative* achieve higher-order interpretive integration across mixed data forms. Similarly, profiles like *trajectory*, *developmental*, and *resilience* demonstrate the iterative, temporal dimension of qualitizing, whereas *ethical*, *value-based*, and *critical* profiles exemplify interpretive reflexivity and value orientation.

Thus, Table 3 operationalizes the *continuum of qualitizing*—from representational multiplicity through analytic synthesis to integrative wholeness. It shows that the 43 narrative profiles are not discrete categories but interrelated expressions of a single methodological ethos: That *meaning emerges through the narrative integration of data, context, and interpretation*. Therefore, Table 3 complements Table 2 by providing the structural scaffolding that unites the typology within the theoretical core of qualitizing.

Rather than emphasizing the recurrence of specific concepts across multiple profile families, the analytic value of the taxonomy lies in the distinct epistemic purposes that differentiate each family of narrative profiles. Each grouping represents a particular mode of narrative reasoning—such as descriptive, developmental, contextual, relational, or transformative—that frames how data are interpreted and integrated. It is precisely this differentiation that enables higher-order analysis across profiles: By comparing how similar phenomena are represented through different narrative logics, researchers can identify meta-level patterns of integration that cut across analytic purposes rather than across repeated concepts. In this way, the taxonomy supports cross-profile analysis not by collapsing distinctions but by making them analytically visible, thereby facilitating a more nuanced and multidimensional understanding of how integration operates within and across narrative forms.

#### Table 3 synthesis: interpretive patterns and conceptual alignments

The conceptual mapping in Table 3 reveals several higher-order patterns that, together, illustrate the evolving logic of qualitizing as instantiated through narrative profiling. First, the *representational continuum* becomes apparent: Foundational profiles such as *modal*, *average*, and *comparative* occupy the descriptive end of the spectrum, emphasizing clarity, generalizability, and central tendency. In contrast, profiles like *transformative*, *ethical*, *critical*, and *resilience* anchor the interpretive end, wherein narrative serves as an instrument for reflexivity, value articulation, and transformation. This gradient demonstrates that the act of qualitizing is scalable—from summarizing patterns within data to reconstituting meaning systems through narrative reasoning.

A second interpretive insight emerging from the typology is that the distribution of narrative profile types can be understood as reflecting a multidimensional continuum of integration, rather than a set of discrete or hierarchically bounded categories. Importantly, reflexivity is not confined to particular profile families, nor does it emerge in a linear progression; rather, it is variably embedded across narrative profile types depending on their epistemic orientation and analytic purpose. Specifically, the typology suggests that narrative profiles vary along intersecting dimensions—for example, from descriptive to transformational orientations and from methodological to interpretive emphases—thereby illustrating the breadth of ways in which integration can be conceptualized and enacted through narrative profiling. In this sense, the typology is best understood not as demonstrating a fixed structure but as offering a conceptual lens for interpreting the diverse forms that integration can take within mixed methods research. This multidimensional distribution reinforces the argument that integration, within the *1 + 1 = 1* integration approach (Onwuegbuzie, 2017; Onwuegbuzie and Hitchcock, 2019), is not a procedural endpoint but continually emerges through the relational interplay of methodological precision, contextual depth, and narrative coherence.

A third interpretive insight is that several narrative profile types, although assigned to a primary profile family for taxonomic clarity, possess conceptual affinities with other families. This does not mean that these profiles appear in multiple groups; rather, it indicates that their analytic logic may resonate across more than one epistemic dimension. For example, a resilience profile might be situated primarily within developmental or transformative reasoning while also carrying contextual or reflexive implications; similarly, a cultural profile may be organized within socio-cultural reasoning while also intersecting with identity, positionality, and ethical interpretation. In this sense, the taxonomy should be understood as a structured but porous framework, in which profile types are assigned to primary families for clarity while remaining open to conceptual overlap, adaptation, and combination in applied research.

A fourth insight involves the *ethical and reflexive dimension* of qualitizing. Profiles such as *ethical*, *moral*, *value-based*, *critical*, and *transformative* foreground the interpretive responsibility of the researcher. They extend qualitizing beyond epistemology into axiology, showing that data transformation is never neutral but always value-laden. Through these profiles, narrative profiling becomes a form of *reflexive integration*—a means of aligning methodological transparency with moral intentionality. In so doing, they strengthen the link between the narrative turn in research and the broader humanistic commitment to meaning, justice, and empathy.

Equally significant are the *relational and structural clusters*, including *relational*, *interactional*, *network*, and *decision-making* profiles. These embody qualitizing as a connective process, one that maps relationships among agents, systems, or decisions to reveal how meaning circulates across networks. They reinforce the premise that integration occurs not only between data types, but also among people, practices, and interpretive communities. Similarly, *cultural*, *intersectional*, *identity*, and *marginalized* profiles demonstrate qualitizing’s capacity to embed narrative within socio-cultural and political contexts, illustrating that integration must account for diversity and positionality as much as for methodological coherence.

Finally, the mapping underscores how *full integration (1 + 1 = 1)* functions as both a process and an outcome across all 43 profiles. For quantitatively anchored forms, integration is achieved through the narrative translation of statistical insights into human terms. For qualitatively anchored forms, it emerges through the systematic incorporation of structure, pattern, and inference into interpretive accounts. For mixed profiles, integration is inherent—realized through reciprocal transformation, wherein quantitative and qualitative strands converge into unified narrative representations. The cumulative insight is that integration is not an endpoint but a representational *state of being*—a mode of knowing in which inference and interpretation coexist seamlessly.

Taken together, these patterns affirm that the expanded typology is more than a catalogue of narrative techniques: It is a *conceptual ecology* that operationalizes the five elements of qualitizing across varied analytic and epistemological terrains. Descriptive, developmental, contextual, ethical, and transformative clusters interact dynamically, demonstrating that qualitizing functions as both an analytic system and an interpretive ethos. In revealing these interconnections, Table 3 transforms the abstract principles of qualitizing into a tangible architecture of practice—one that captures the full complexity, reflexivity, and integrative potential of narrative profiling in contemporary mixed methods research.

### Meta-grouping of the 43 narrative profile types within qualitizing

Following the detailed typology and structural mapping presented in Tables 2, 3, Table 1 synthesizes the 43 narrative profile types into higher-order *meta-categorical groupings* that reflect their shared analytic logics, epistemological orientations, and methodological purposes. Whereas Table 2 depicted the profiles as discrete representational forms and Table 3 mapped their correspondence to the five major elements of qualitizing, Table 1 operates at a meta-level of conceptual organization. It shows how these profiles cluster into coherent *families of narrative reasoning*, thereby revealing the deep structural and functional architecture of qualitizing as both a process and a philosophy of integration.

The purpose of Table 1 is twofold. First, it provides an organizational lens for understanding the expanded typology not as 43 isolated techniques but as a system of interrelated analytic orientations. Second, it clarifies how different narrative profiles serve complementary roles in the process of qualitizing—from descriptive to interpretive, from structural to reflexive, and from empirical to ethical. In doing so, Table 1 makes explicit the layered nature of narrative integration: How specific representational forms combine to produce holistic understanding across the continuum of mixed methods inquiry.

The table arranges the profiles into nine meta-categories, each defined by a dominant analytic and methodological orientation. Although these nine meta-categories are conceptually comprehensive, they are not exhaustive. Rather, they represent a theoretically grounded and generative framework for organizing the principal analytic and methodological orientations through which narrative profiling currently operates. Each meta-category captures a family of narrative reasoning that structures the 43 profile types at this stage of theoretical development, while remaining open to future expansion as new hybrid forms and epistemological innovations emerge. These categories represent thematic constellations rather than rigid taxonomic classes, acknowledging that many profiles function at the intersections of multiple domains. For example, the *holistic* and *transformative* profiles are intentionally cross-listed because their integrative character spans both experiential and temporal dimensions of meaning-making.

Each group encapsulates a distinct mode of qualitizing practice:

1. Foundational Profiles—(*modal, average, holistic, comparative, normative*)—anchor the typology in the original framework of Tashakkori and Teddlie (1998), representing the essential descriptive, comparative, and representational bases upon which all other profiles are constructed. They emphasize typicality, frequency, and benchmarked understanding.
2. Exemplar and Pattern-Based Profiles—(*exemplar, cluster, aggregated, latent, behavioral, predictive, causal, thematic, sentiment*)—extend this foundation into the analytic domain, focusing on identifying emergent structures, typologies, and predictive or causal relationships within data. These profiles are distinguished by their emphasis on identifying, organizing, and narratively representing underlying patterns, structures, or groupings within data—such as clusters, latent classes, thematic configurations, or typological distinctions. Unlike foundational descriptive profiles, which summarize central tendencies, exemplar and pattern-based profiles highlight relational structure and variability across cases, transforming analytic pattern detection into coherent narrative representations that make latent organization and subgroup differentiation interpretable.
3. Temporal and Developmental Profiles—(*dynamic, trajectory, evolutionary, developmental, historical, event-centric, narrative arc, transformative*)—capture processes of change, adaptation, and growth. They embody the temporal logic of narrative, translating statistical or thematic shifts into coherent developmental storylines.
4. Contextual and Environmental Profiles—(*contextual, comparative contextual, context-sensitive, geographic, spatial*)—situate findings within ecological, spatial, or situational settings, underscoring that data derive meaning only through their embeddedness in context.
5. Cultural, Identity, and Social Positioning Profiles—(*cultural, intersectional, marginalized, identity, persona*)—foreground the interpretive construction of self and social reality, emphasizing diversity, positionality, and the reflexive awareness of voice and representation.
6. Meaning, Value, and Ethical Profiles—(*value-based, ethical, moral, aspirational, critical*)—introduce the axiological dimension of qualitizing, wherein interpretation is inseparable from values, ethics, and transformative intent. They connect narrative profiling to reflexivity, social justice, and moral reasoning.
7. Relational and Interactional Profiles—(*relational, interactional, conflict, network, decision-making*)—focus on human relationships, communication, and collaboration, revealing how meaning is co-constructed through interaction and dialogue.
8. Experiential and Reflective Profiles—(*experiential, resilience, holistic*)—illuminate phenomenological and introspective dimensions of qualitizing, capturing lived experience, adaptation, and reflective transformation. They bridge analytic structure with emotional and embodied understanding.
9. Analytic and Structural Profiles (Cross-Cutting Group)—(*aggregated, cluster, latent, network, causal*)—span multiple categories by providing methodological scaffolding for integration. These profiles serve as connective tissue across the typology, modeling the analytic mechanisms that enable narrative synthesis.

Collectively, these nine groupings show that the expanded typology is *systemic and hierarchical* rather than additive or fragmented. Each group corresponds to a distinct epistemic purpose within qualitizing—whether describing, patterning, contextualizing, positioning, or transforming—and, together, they illustrate the multidimensional architecture through which quantitative and qualitative reasoning are narratively fused.

#### Table 1 synthesis: higher order patterns and integrative insights

The meta-categorical grouping presented in Table 1 yields several overarching insights into the internal logic of the expanded typology and, by extension, into the evolving nature of qualitizing itself. First, it reveals that *qualitizing operates through interlocking layers of reasoning*—descriptive, analytic, contextual, relational, and reflexive—each representing a necessary dimension of integration. Foundational and exemplar profiles serve as the structural base, ensuring analytic rigor and representational clarity. Temporal and contextual families add dynamic and situational nuance, whereas ethical and experiential profiles reintroduce the interpretive, value-based, and humanistic dimensions that anchor the narrative turn.

Second, the groupings demonstrate that integration is distributed across the system, not confined to a single act of data merging. Structural profiles like *cluster* and *aggregated* operationalize integration at the methodological level, whereas reflective profiles like *ethical* and *transformative* realize it at the interpretive level. This multi-level distribution reinforces the argument that full integration (i.e., the *1 + 1 = 1* integration approach; Onwuegbuzie, 2017; Onwuegbuzie and Hitchcock, 2019) is not a procedural endpoint but a *meta-condition of inquiry* that emerges through the relational interplay of methodological precision, contextual depth, and narrative coherence.

A third insight concerns the *cross-cutting nature of certain profiles*, particularly *holistic*, *transformative*, and *aggregated*, which appear in multiple groups. Their recurrence underscores their function as *integrative nodes*—profiles that bridge categories by embodying several analytic and epistemic dimensions simultaneously. This fluidity reflects the pluralism inherent in mixed methods reasoning: the capacity to think narratively across levels of abstraction and types of evidence.

Finally, the meta-grouping reveals a thematic progression across the typology—from description (*foundational* and *pattern-based* profiles), to interpretation (*contextual*, *cultural*, *identity* profiles), to transformation (*ethical*, *critical*, *transformative* profiles). This progression mirrors the developmental arc of qualitizing itself as it evolves from methodological technique to interpretive paradigm. It illustrates that the end point of qualitizing is not merely the production of integrated data but the cultivation of integrated understanding—a synthesis of analytic, moral, and human insight expressed through narrative form. Collectively, these nine categories delineate the current architecture of narrative qualitizing while preserving conceptual openness for future elaboration as the field continues to evolve.

Taken together, the insights from Table 1 confirm that the 43 narrative profile types function not as a loose collection of strategies but as a *coherent meta-framework for integration*. They collectively embody the movement from data toward meaning, from representation toward reflexivity, and from methodological synthesis toward interpretive wholeness. In so doing, they demonstrate that the future of qualitizing lies not only in methodological innovation but also in cultivating a narrative consciousness capable of holding complexity, diversity, and coherence in dynamic relation.

## Analytical significance of the 43-profile taxonomy

The 43-profile taxonomy advances qualitizing not only as a representational innovation, but also as a transformation in the logic of analysis itself. Each narrative profile type formalizes a distinct *mode of analytic reasoning*—a patterned way of moving from data to meaning through the integrative interplay of inference and interpretation. In this sense, the taxonomy extends qualitizing from a descriptive act of converting numbers into words to an epistemic system for generating, testing, and refining meaning through narrative synthesis. It provides the analytical scaffolding through which researchers can trace how quantitative tendencies, qualitative insights, and interpretive judgments converge within a single, coherent framework of integration.

Conceptually, the taxonomy operates as an *analytic grammar* for mixed methods inquiry. Each profile type embodies an implicit rule of translation and transformation—what might be called the “syntax” of qualitizing—by which patterns in data are reconfigured into coherent narratives. Foundational profiles such as modal, average, and comparative enact descriptive reasoning, translating statistical regularities into interpretive generalizations. Developmental and temporal profiles embody sequential reasoning, narrating change and progression. Reflexive and ethical profiles instantiate evaluative reasoning, integrating values, positionalities, and moral insights into the analytic process. Collectively, these modes of reasoning delineate a pluralistic system of analysis that moves beyond binary distinctions between quantitative and qualitative operations—reflecting a synechistic (i.e., anti-dualistic) orientation (Johnson and Gray, 2010) and aligning with Johnson’s (2023) advocacy of a *both/and* stance, also known as the logic of combination, as opposed to an *either/or* stance—illustrating that analysis itself can be narratively structured.

Analytically, the taxonomy introduces a logic of *multi-dimensional integration*. In traditional mixed methods analysis, integration often occurs post-hoc, after separate strands of data analysis are completed (cf. Fetters and Freshwater, 2015; *1 + 1 = 3 integration approach*). In contrast, narrative profiling embeds integration within the analytic process itself. Profiles such as transformative, relational, and resilience enact what might be termed *intra-analytic integration*, wherein the construction of meaning arises through the ongoing synthesis of inferential and interpretive reasoning. Quantitative indicators and qualitative accounts are not merely juxtaposed but are mutually reconstituted through narrative logic, yielding analytical outcomes that are both generalizable and contextually grounded. This embeddedness of integration transforms qualitizing from a procedural step into a *mode of analytical cognition*—a way of thinking through data narratively.

The taxonomy also enhances *analytical reflexivity*, making the interpretive agency of the researcher both visible and systematic. Each narrative profile requires explicit methodological choices about focus, voice, and interpretive stance. For instance, an ethical profile demands reflexive articulation of the researcher’s moral positioning, whereas a comparative contextual profile necessitates critical awareness of boundary conditions and contextual contingencies. In this way, the taxonomy renders reflexivity an analytic act rather than a post-analytic reflection. It invites researchers to trace how interpretation is constructed, justified, and narrated, positioning transparency and accountability as integral components of analytic rigor.

Equally significant is the capacity of the 43-profile taxonomy to expand the *analytic bandwidth* of mixed methods research. By formalizing a repertoire of 43 integrative profiles, it allows for a fine-grained selection of analytic strategies matched to specific research purposes. A researcher seeking to identify emergent typologies might employ cluster profiles or exemplar profiles; a longitudinal intervention study might draw on developmental profiles or trajectory profiles; a participatory project might utilize relational profiles or transformative profiles to capture collective meaning-making processes. In this respect, the taxonomy functions as both a design framework and an analytic toolkit, supporting methodological precision while maintaining interpretive richness. It offers a means to operationalize integration at multiple analytic levels, from micro-interpretive case synthesis to macro-level narrative modeling.

From a theoretical standpoint, the taxonomy’s analytical significance lies in how it redefines *what counts as analysis* in mixed methods research. Within postpositivist traditions, analysis often has been equated with measurement, estimation, or categorization—acts that privilege numerical or thematic reduction. Within interpretivist traditions, it has emphasized meaning construction through inductive narrative or thematic elaboration. The 43-profile taxonomy dissolves this dichotomy by demonstrating that analysis is not the domain of one tradition but rather the *integrative process* that connects them. Each narrative profile represents a hybrid analytical form, wherein measurement informs meaning, and meaning recontextualizes measurement. In so doing, it advances qualitizing as an epistemic technology of synthesis: a method for thinking simultaneously in analytic patterns and in narrative stories.

In this context, the term *analytic patterns* refers to abstracted regularities, structures, or relationships identified through quantitative, qualitative, or mixed methods analysis (e.g., distributions, clusters, associations, or thematic configurations), rather than to the “pattern-based” narrative profile family within the present taxonomy. Thus, patterns and stories are not intended to represent opposing constructs; rather, they reflect complementary modes of reasoning. Patterns capture the structural organization of data, whereas stories recontextualize these structures within meaning-centered, temporally situated narratives. Narrative profiling operates at the intersection of these modes by transforming analytic patterns into storied representations.

The taxonomy also provides a foundation for *meta-analytic reasoning* within qualitizing. Because it organizes analytic approaches into families of reasoning—foundational, developmental, contextual, ethical, relational—it enables researchers to analyze across profiles, identifying higher-order patterns of integration. For example, the recurrence of reflexivity and temporality across several profile clusters suggests that narrative logic inherently privileges process and perspective rather than static representation. Thus, the taxonomy becomes not only a guide to analytic practice, but also a heuristic for theorizing how integration itself unfolds—how meaning is built, layered, and reconstituted through iterative acts of narrative interpretation.

In sum, the analytical significance of the 43-profile taxonomy is reflected in its capacity to illuminate how diverse narrative profile types support multiple, complementary modes of integration within mixed methods research. It makes visible the interpretive architectures that underlie integration, providing researchers with a structured yet flexible framework for constructing analytically rigorous and narratively coherent accounts of data. More than a typology, it is a *theory of analytic action*: a demonstration that qualitizing achieves its highest potential not as a bridge between methods, but as an analytic worldview—one in which inference, interpretation, and narrative are inseparable dimensions of human understanding.

## Relationship to *on qualitizing* and mixed methods research integration

The conceptual and analytical architecture of the present taxonomy is grounded in, and simultaneously extends, the foundational principles articulated in Onwuegbuzie and Leech’s (2019) *On Qualitizing*. This original work established qualitizing as a central mechanism of integration in mixed methods research—defined as the process through which quantitative data are transformed into qualitative meaning to facilitate a fuller, more interpretive understanding. This formulation represented a methodological correction to the long-standing asymmetry in the literature, wherein quantitizing—transforming qualitative data into numerical indicators—had received far greater procedural attention and theoretical development (Sandelowski et al., 2009). By formally delineating the five major elements of qualitizing—multiple representational forms, bidirectionality of transformation, dual analytic engagement, iterative processes, and full integration—Onwuegbuzie and Leech (2019) provided the first meta-framework for understanding how numerical information could be rendered narratively intelligible.

The present work builds directly on this foundation but advances the discussion in several critical ways. Whereas *On Qualitizing* conceptualized transformation largely in terms of analytic form—the movement of data from quantitative expression to qualitative interpretation—*On Qualitizing Revisited* reconceptualizes this process as a *representational act of narrative construction*. In this expanded formulation, qualitizing is not only a mode of data conversion, but also a mode of meaning-making, wherein narrative becomes the integrative grammar through which quantitative and qualitative logics are reconciled. The introduction of the 43-profile taxonomy concretizes this reframing by translating the abstract dimensions of qualitizing into operational narrative forms. Each narrative profile type represents a specific instantiation of the five elements identified by Onwuegbuzie and Leech (2019), illustrating how multiple representations, analytic flexibility, and full integration are realized through the narrative logic of storying.

In this sense, the relationship between *On Qualitizing* and the present framework is both *genealogical* and *generative*. Genealogically, narrative profiling preserves the structural integrity of the original meta-framework: It remains anchored in the five core elements and their commitment to methodological integration as an interpretive act. Generatively, it extends these principles by elaborating the representational spectrum through which integration can occur. The 43-profile taxonomy thus operationalizes what Onwuegbuzie and Leech (2019) termed *full integration* (i.e., *1 + 1 = 1*) not merely as a procedural goal but as a *representational reality*—a narrative synthesis in which quantitative inference and qualitative interpretation converge within a unified storied account. This shift from integration as *methodological sequence* to integration as *narrative ontology* constitutes a significant theoretical advancement within mixed methods research.

Within the broader context of mixed methods integration, this framework aligns closely with, yet also refines, the evolving understandings of integration advanced by other scholars. Through an arts-based mixed methodological movie script, Onwuegbuzie (2024c) distinguished between partial (i.e., *1 + 1 = 3*) and full (i.e., *1 + 1 = 1*) integration, underscoring that the highest form of mixed methods synthesis is achieved not by juxtaposition but by fusion. Narrative profiling materializes this fusion in representational form: Rather than merging datasets mechanically, it integrates them narratively, producing interpretive coherence at the level of meaning rather than method alone. Similarly, the taxonomy resonates with Creswell and Plano Clark’s (2018) call for integration to occur across all phases of research—from design through interpretation—by embedding integration within the analytic process itself. Through narrative logic, integration becomes recursive and reflexive, occurring not only at the conclusion of analysis but throughout the interpretive construction of profiles.

The narrative profiling framework also deepens the philosophical commitments implicit in *On Qualitizing*. Onwuegbuzie and Leech’s (2019) original formulation was grounded in social constructionism, emphasizing that data—whether quantitative or qualitative—are not self-evident entities but interpretive artifacts shaped by context, perspective, and representation. The present article advances this constructionist foundation into a fully CDP-based interpretive epistemology, wherein *narrative itself is the medium of integration*. Data are no longer simply converted into another form; they are *revoiced* within storylines that reveal the relational, temporal, and value-laden dimensions of human meaning. This revoicing expands the scope of integration from epistemic coherence (consistency of findings) to hermeneutic coherence (consistency of meaning), thereby aligning qualitizing with the interpretive and narrative turns in the human sciences (Bruner, 1990; Polkinghorne, 1995; Riessman, 2008).

Importantly, the narrative profiling approach extends the principle of *bidirectionality*—central to *On Qualitizing*—to its full potential. Although Onwuegbuzie and Leech (2019) defined qualitizing primarily as transforming quantitative data into qualitative meaning, they also acknowledged that the relationship between data forms is reciprocal. The 43-profile taxonomy embodies this reciprocity by providing representational modes that can move in either direction: Quantitative data may be storied (qualitizing), and qualitative data may be structured or patterned (quantitizing) within the same analytic space. This reciprocal capacity situates narrative profiling squarely within the logic of *integration through transformation*, demonstrating that the distinction between qualitizing and quantitizing is methodological rather than ontological—each representing different pathways toward meaning-centered synthesis.

In this regard, the present framework also contributes to the ongoing discussion of *integrative pluralism* in mixed methods research (Greene, 2007; Tashakkori et al., 2020). Narrative profiling exemplifies pluralism not as methodological eclecticism but as interpretive synergy—a logic of combination wherein diverse data forms, analytic procedures, and representational modes coexist within a single coherent account. The taxonomy illustrates how pluralism can be enacted through narrative design, allowing researchers to blend precision and depth, generalization and particularity, and inference and interpretation. This narrative pluralism mirrors the synechistic orientation (Johnson and Gray, 2010) that underlies mixed methods research philosophy, rejecting dualistic separations between quantitative and qualitative reasoning and, instead, embracing the continuity of meaning that unites them.

Taken together, the relationship between *On Qualitizing* and the narrative profiling framework represents a shift from *methodological integration* to *representational integration*—from *combining data* to *combining ways of knowing*. The 43-profile taxonomy provides the analytic and narrative infrastructure through which this integration is realized, transforming qualitizing from a procedural technique into an interpretive worldview. In doing so, it repositions mixed methods research not simply as a combination of techniques but as a form of narrative inquiry into complexity—an approach that unites statistical inference and narrative imagination within a single, coherent act of understanding.

Having articulated the analytical significance of the 43-profile taxonomy, the next logical step involves demonstrating its empirical applicability. Although the taxonomy operates as a conceptual and methodological framework, its ultimate value lies in how effectively it can be mobilized to interpret real-world data. Cross-referencing the narrative profile types with empirical datasets enables researchers to test, to refine, and to substantiate the taxonomy’s analytic propositions, illustrating how abstract representational logics manifest in concrete research practice. This empirical anchoring transforms the taxonomy from a theoretical schema into a dynamic instrument of inquiry—one capable of tracing how data become meaning through the iterative, integrative, and narrative processes that define qualitizing.

Having established how the present framework both extends and deepens the theoretical foundations of qualitizing and mixed methods research integration, the next section outlines its broader theoretical, methodological, pedagogical, and practical implications for research design and analysis.

## Theoretical and methodological implications

The expanded 43-profile taxonomy of narrative profiling carries significant theoretical, methodological, and pedagogical implications for the future of mixed methods research. Theoretically, it redefines qualitizing not as a peripheral act of data translation but as an epistemic architecture for constructing meaning across traditions. By positioning narrative profiling as the central mechanism of interpretive integration, the taxonomy provides a unifying grammar through which quantitative and qualitative logics converge within a single analytic worldview. In doing so, it advances social constructionism in practice—treating data as co-constructed meaning systems rather than as independent representations of reality.

Methodologically, the taxonomy extends mixed analysis beyond transformation toward full analytic integration. It operationalizes the *1 + 1 = 1* integration approach by embedding integration within each representational act rather than reserving it for post-hoc synthesis. This reframing shifts integration from a procedural goal to an analytic condition, wherein quantitative precision and qualitative depth coexist within every profile. Moreover, the taxonomy offers a replicable yet flexible structure for researchers seeking to design, to analyze, and to interpret data narratively across disciplines. It transforms qualitizing from a descriptive tool into a generative analytic process capable of producing coherent, multidimensional narratives of human experience.

Pedagogically, the taxonomy functions as a heuristic for teaching qualitizing and narrative integration. Its typological clarity provides educators and emerging scholars with a structured repertoire of approaches for representing mixed data narratively. By tracing how each profile embodies specific analytic logics—descriptive, interpretive, reflexive, or transformative—students can learn to design consciously and transparently integrated analyses. This pedagogical function also promotes reflexivity and ethical awareness by highlighting the interpretive agency involved in all acts of transformation. More specifically, this ethical awareness emerges from the recognition that narrative profiling is not a neutral representational act but one that involves consequential decisions about whose voices are emphasized, how experiences are interpreted, and how meaning is constructed and conveyed. In learning to apply different narrative profile types, researchers and students are required to reflect on the implications of these choices, including issues of representation, potential bias, and the responsible translation of data into narrative form. In this way, the pedagogical use of the taxonomy makes visible the ethical dimensions of qualitizing, emphasizing that the transformation of data into meaning-centered narratives carries obligations related to fidelity, transparency, and respect for the complexity of participants’ experiences.

Practically, the 43-profile taxonomy provides a methodological toolkit adaptable to diverse phenomena, data types, and disciplinary settings. Researchers can select or combine profiles to match their analytic purposes, thereby enacting integration as a dynamic, context-sensitive process rather than as a prescriptive formula. Through this adaptability, the taxonomy positions narrative profiling as the epistemic core of fully integrated mixed methods research—a meta-framework that not only bridges methods, but also deepens understanding of how data acquire meaning. These theoretical and methodological implications come to life most vividly when applied to empirical data. The following heuristic illustration demonstrates how the principles of qualitizing and narrative profiling can be enacted in practice, using a reanalysis of Onwuegbuzie et al.’s (2011) foundational mixed methods research study.

## Heuristic illustration: revisiting a foundational mixed methods research study

To clarify the purpose of this heuristic illustration, it is important to emphasize that the goal is not merely to present narrative accounts of participants’ experiences but to demonstrate how the narrative profiling taxonomy can be applied as a systematic qualitizing procedure. Accordingly, the examples that follow are structured to illustrate how different forms of data—quantitative, qualitative, or mixed—can be transformed into narrative profiles that embody analytic integration. In this sense, the illustration is intended to function not as a descriptive presentation of findings but as an explicit demonstration of how interpretive synthesis is achieved through the application of specific narrative profile types.

To illustrate concretely how the process of qualitizing through narrative profiling functions in practice—and to demonstrate the value of the 43-profile meta-framework—the following section revisits the same heuristic study used by Onwuegbuzie and Leech (2019): Onwuegbuzie et al. (2011). By reanalyzing this study through the lens of narrative profiling, particularly the *context-sensitive profile*, the example demonstrates how the expanded framework transforms analytically derived typologies into human-centered narrative syntheses.

### Heuristic example: revisiting the article, *“A Mixed Research Study of Pedagogical Approaches and Student Learning in Doctoral-Level Mixed Research Courses,”* through narrative profiles

Using the same heuristic example as Onwuegbuzie and Leech (2019), this heuristic example aims to go beyond what those authors did in using Onwuegbuzie et al.’s (2011) study to exemplify their five-element typology of qualitizing. Specifically, the goal here is to show how Onwuegbuzie et al. (2011) could have derived significantly more interpretive and pedagogical depth from their data by using narrative profiles—particularly the expanded 43-profile meta-framework.

### Summary of the heuristic study

In their mixed methods research investigation, *A Mixed Research Study of Pedagogical Approaches and Student Learning in Doctoral-Level Mixed Research Courses* (Onwuegbuzie et al., 2011), the authors primarily sought to compare and to contrast the pedagogical approaches used by first-generation instructors of mixed methods research courses. Eight instructors (4 women, 4 men) representing diverse U. S. institutions were interviewed using semi-structured interviews. The qualitative data were analyzed via constant comparison analysis (Glaser, 1965), leading to the emergence of three metathemes that each represented a pedagogical dimension:

1. Orientation (Methodologically Focused vs. Question/Topic Focused)
2. Level of Application (Conceptual vs. Applied)
3. Level of Structure (Structured vs. Exploratory)

Each of these three metathemes—representing a distinct pedagogical dimension—comprised two contrasting themes, as identified earlier in parentheses. The *Orientation dimension* ranged along a continuum from *Methodologically Focused* to *Question/Topic Focused*. At the *Methodologically Focused* end were instructors who devoted substantial class time to discussing quantitative research, qualitative research, and mixed methods research traditions, emphasizing methodological traditions and their underlying assumptions. At the opposite end, *Question/Topic Focused* instructors centered their pedagogy on guiding students in how to conduct mixed methods research studies with minimal or no explicit attention to methodological traditions, instead emphasizing that the research question or topic should determine the methodological approach.

The *Level of Application dimension* spanned a continuum from *Conceptual* to *Applied*. Courses positioned at the *Conceptual* end emphasized abstract understanding and theoretical discussion of mixed methods research principles, whereas those at the *Applied* end required students to engage directly in empirical practice—collecting and analyzing real data and producing complete mixed methods research reports.

Finally, the *Level of Structure dimension* extended from *Structured* to *Exploration*. At the *Structured* pole were courses characterized by high levels of organization and guidance, in which students were introduced to models, typologies, and frameworks and were expected to employ these conceptual tools to shape their learning. At the *Exploration* pole were courses emphasizing experiential learning and open-ended inquiry, providing students with greater autonomy to construct their own understanding of mixed methods research.

**Figure 1:**
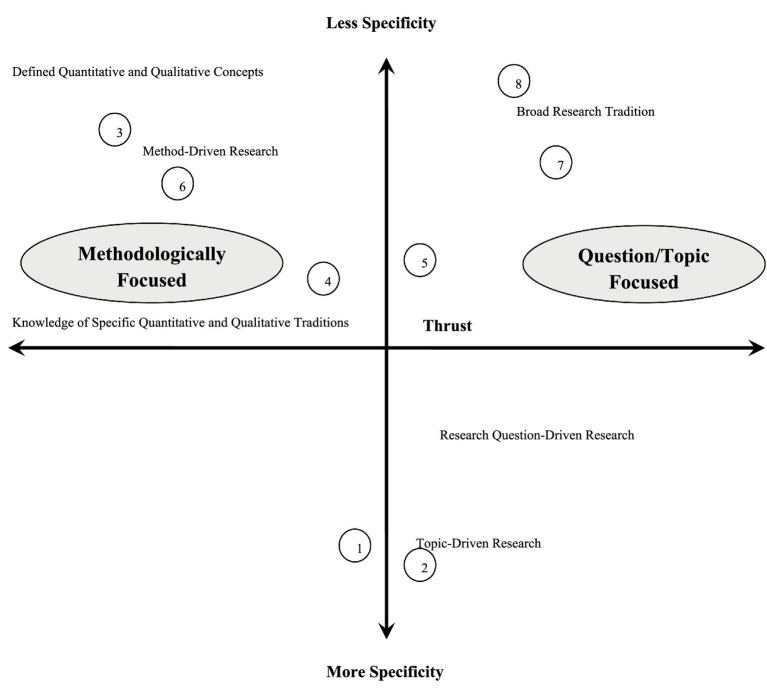
Metatheme of orientation: Methodological focused pedagogy versus research focused pedagogy. Adapted from Onwuegbuzie et al. (2011, p. 182). Copyright 2011 by Dialectical Publishing LLC.

These dimensions reflected how instructors differed in their conceptual and practical approaches to teaching mixed methods research. Using QDA Miner, the qualitative data then were quantitized by constructing matrices of co-occurrences among themes within each metatheme. These matrices were subjected to a series of correspondence analyses—a multivariate statistical analysis—generating multidimensional maps. As an example, one correspondence plot (i.e., Figure 1) illustrated how the eight instructors were positioned along the *Orientation* continuum. As shown in Figure 1, four instructors were methodologically focused (Instructors 1, 3, 4, 6), whereas the remaining four instructors were question/topic focused (Instructors 2, 5, 7, 8). Despite clustering patterns, each instructor demonstrated unique pedagogical nuances—what, using Miles and Huberman’s (1994) terminology, Onwuegbuzie et al. (2011) referred to as *partially ordered meta-matrices*, which displayed the pedagogical profiles of each participant as a function of her/his orientation (i.e., Metatheme 1), level of application (i.e., Metatheme 2), and level of structure (i.e., Metatheme 3).

Comparing across dimensions, Onwuegbuzie et al. (2011) developed five pedagogical profiles, each representing a modal combination of the three metatheme dimensions (see Table 4). For example, three instructors exhibited the *question/topic focused–applied–structured (QAS*) *profile*, reflecting courses in which the research question drove methodological choice, students engaged in real data collection, and instruction followed a structured framework.

**Table 4:** Partially ordered meta-matrix: pedagogical profiles of each of the participants as a function of orientation, level of application, and level of structure.

Participant	Orientation	Application	Structure	Total in group
**2**	Question/Topic	Applied	Structured	
**5**	Question/Topic	Applied	Structured	
**7**	Question/Topic	Applied	Structured	3
**1**	Methodological	Conceptual	Exploratory	
**6**	Methodological	Conceptual	Exploratory	2
**8**	Question/Topic	Conceptual	Exploratory	1
**3**	Methodological	Conceptual	Structured	1
**4**	Methodological	Applied	Structured	1

Adapted from Onwuegbuzie et al. (2011, p. 193). Copyright 2011 by Dialectical Publishing LLC.

Onwuegbuzie et al. (2011) described each profile narratively and noted that these pedagogical profiles provided a typology for understanding variation in mixed methods research teaching. Indeed, the study was among the first to apply mixed methods research techniques to the pedagogy of mixed methods research itself, and its five-profile typology has since been validated by numerous instructors.

### Extending the heuristic example through narrative profiles

Although it could be argued that the five-profile typology presented by Onwuegbuzie et al. (2011) is rich, systematic, and conceptually insightful, it could have been even more compelling, humanized, and pedagogically resonant through the incorporation of narrative profiles derived from the 43-profile meta-framework. In fact, narrative profiling would have transformed the typology from a structural model into a *living interpretive portraiture*—capturing the voices, reflexivities, and contextual complexities of each instructor’s teaching philosophy. To illustrate this, one of the instructors—Participant 2, one of the three instructors categorized under the QAS profile—is revisited here through the lens of the *context-sensitive profile* from the 43-profile meta-framework.

#### Justification for the context-sensitive profile

Box 1 presents the rationale for identifying Participant 2 as exemplifying the *context-sensitive profile*. It is important to note that individual-level narrative profiles are not intended to function as standalone descriptive accounts of a single participant’s experiences. Rather, within the framework of qualitizing, such profiles serve as analytically constructed representations that emerge from systematic engagement with the data. To align with this purpose, the following individual-level illustration should be interpreted as an initial, partial representation that requires further transformation and integration within a broader analytic framework. On its own, an individual narrative—if presented primarily as a descriptive summary—does not fully instantiate qualitizing, which requires the synthesis, abstraction, or integration of data into higher-order meaning structures.

BOX 1 **Justification for the context-sensitive profile.**

The participant most clearly embodies the *Context-Sensitive Profile* because her teaching philosophy and practices are shaped by continuous responsiveness to situational, institutional, and relational contexts. Her narrative reveals a pedagogy that adapts fluidly to the structure of her course, the diversity of her students, and the evolving nature of mixed methods research itself. She teaches in a compressed three-weekend format and transforms this limitation into a strength by creating continuity through short quizzes, reflective dialogue, and intensive mentoring. Her approach to defining mixed methods research also demonstrates epistemological flexibility; she shifted from insisting on a single definition to inviting multiple perspectives, noting that “the field is still emerging.”

Her responsiveness extends to ethical and relational dimensions as well. Drawing on her experience with the Institutional Review Board (IRB), she embeds ethics throughout her teaching, emphasizing that it should be “an ongoing practice, not just a onetime IRB activity.” She also personalizes learning through sustained mentorship, ensuring that she “checks in with everybody in the class” and remains “available by email 24 h a day.” Finally, she situates her reflections within the broader ecology of doctoral education, arguing that “we are short-changing mixed methods” by restricting it to one course. Collectively, these insights reflect the hallmark of a *Context-Sensitive Profile*—pedagogy as a living, adaptive process shaped by and responsive to the contexts in which it unfolds.

As shown in the box, her teaching philosophy reflects an adaptive, relational, and ethically grounded responsiveness to the multiple contexts in which she teaches—temporal, institutional, and interpersonal. She transforms the constraints of a condensed 3-weekend format into opportunities for sustained engagement, fosters dialogical exploration of mixed methods research definitions, integrates ethics throughout the research process, and maintains close mentoring relationships with her students. Her reflections further reveal structural awareness of the limitations of doctoral curricula, advocating for deeper institutional commitment to mixed methods research pedagogy. Collectively, these elements capture the defining feature of the context-sensitive profile—teaching as a dynamic and contextually attuned act of integration.

#### Context-sensitive profile: the reflective practitioner in mixed methods research pedagogy

Box 2 presents the full *context-sensitive profile: the reflective practitioner in mixed methods research pedagogy*, which encapsulates Participant 2’s narrative as an example of contextually adaptive teaching. As shown in the box, her pedagogy is characterized by reflexivity, ethical integration, and relational attentiveness—balancing structure with openness and methodological precision with dialogical engagement. She transforms the constraints of an intensive 3-weekend course into a dynamic learning environment through continuity-building activities, experiential assignments, and sustained mentorship. Her responsiveness extends beyond classroom practice to ethical and institutional concerns, embedding moral reflection into methodological instruction and advocating for a more robust curricular treatment of mixed methods research. Collectively, this profile portrays an educator who embodies the ethos of integration itself—teaching as an adaptive, reflective, and human-centered process attuned to context, complexity, and the evolving nature of inquiry.

BOX 2 **Context-sensitive profile of participant 2: the reflective practitioner in mixed methods research pedagogy.**

Participant 2’s teaching philosophy is grounded in responsiveness and reflexivity, a continual recalibration of approach shaped by the contexts in which teaching and learning unfold. Her lived experience as a mixed methods research instructor reflects the balance between structure and openness, between precision and dialogue, and between the pragmatic constraints of course delivery and the deeper aspiration to cultivate authentic understanding. Reflecting on her earlier teaching, she noted that “the last time I taught it, I was very structured about how I wanted everyone to think the way I thought. I wasn’t open to talking with them,” recognizing that this rigidity limited her students’ engagement with the evolving nature of mixed methods research. Over time, she learned that openness to diverse perspectives strengthens learning: “I’d rather have that conversation first and then say, well this is the definition that I like, and then also present other people’s definitions so that they realize that the field is still emerging.” Her evolution as an educator mirrors the epistemological stance of mixed methods research itself—one that values multiplicity and invites dialogue.

Her reflections reveal a pedagogy deeply attuned to the diversity of learners and the complexity of institutional context. Each course brings together students from “multiple schools within the university—nursing, education, public affairs, and health and behavioral sciences,” each entering with “varying levels of understanding of qualitative and quantitative research.” She acknowledges the challenge of bridging these disciplinary dialects: “Especially with qualitative research… someone will be talking about narrative research, and someone else will be talking about ethnography, but they really mean about the same thing.” In response, she deliberately constructs a shared language, offering overviews of both qualitative and quantitative research traditions so that “we are all on the same page and we can talk to each other about these.” This attention to linguistic and epistemic coherence transforms her classroom into a collaborative space of translation and synthesis—a microcosm of the very integration that defines mixed methods inquiry.

Time, structure, and pacing also emerge as contextual forces that shape her teaching. Unlike traditional semester-long courses, her mixed methods research course is delivered “in three weekends—Friday night and Saturday for three different weekends throughout the semester.” She acknowledges that this design “becomes a challenge for students,” particularly because “they do not have that weekly recharge of coming into class and everybody talking about mixed methods and getting back into that frame of thinking.” The compressed format requires creativity and intentionality. In order to sustain engagement, she uses “short quizzes… mainly because of the three weekends” to “give them a sense that they better be doing the reading” and to “facilitate a review for what we talked about the last weekend.” Within this structure, she embeds opportunities for reflection and reconnection, weaving continuity through pedagogical design even when temporal gaps threaten to fragment learning.

Participant 2’s course centers on experiential learning through a signature assignment: “They have to do their own mixed methods study.” This project-based approach invites students to inhabit the role of the researcher, confronting real methodological tensions rather than merely theorizing about them. “I try to keep the project as a mini dissertation,” she explained, “and many students do something similar to what they want to do for their dissertation so that they have a practice run of what they’ll be doing in the larger sense.” Through this design, she transforms her classroom into a rehearsal space for scholarly identity formation, one where learners discover the gaps in their understanding. “It helps them to realize really what goes into a mixed methods study, and it helps them to see the holes in their learning.” This experiential immersion situates learning within the context of authentic research practice, emphasizing process over perfection and growth over performance.

Her relational ethic of teaching is equally central. Despite the constraints of format and time, she cultivates a deeply personal connection with her students. “I make myself available by email 24 h a day,” she explained, noting that “many students choose to send me these long emails explaining their issues so that I can help them.” During class weekends, she uses informal moments to build rapport and monitor progress: “During lunch time I’m available—I’ll sit in the classroom and eat my lunch and they can stay and talk to me… and by the end of the day I make sure I’ve checked in with everybody in the class.” These intentional gestures transform logistical necessity into human connection. Her attentiveness allows her to “know who’s struggling with what, who needs help with what, and who’s doing really well,” enabling her to tailor support responsively. In her teaching, care is not ancillary—it is infrastructural.

Ethics occupies a prominent place in her pedagogy, not as a compliance task but as a living thread woven throughout the course. Drawing on her experience on the Institutional Review Board, she either “has somebody from the IRB come in and talk” or leads discussions herself, emphasizing that “students and most researchers need to think through the ethics of research on an ongoing basis.” She integrates ethical reflection into methodological content: “When we talk about sampling, we also talk about ethics you need to consider when you are doing sampling, or when you are talking about data collection.” This integration transforms ethics from a compartmentalized topic into a sustained moral orientation, one that shapes how students see their roles as researchers. She acknowledges that this approach “keeps me more on my toes of what ethical things are happening,” as she personally takes responsibility for ensuring that “it is an ethical study.” Through this embedded practice, she models an ethic of care that extends beyond regulatory compliance to the cultivation of moral mindfulness.

Her reflections also reveal a critical awareness of structural constraints in doctoral education. She challenges the institutional norm that “mixed methods has to be one course,” arguing that “we are short-changing mixed methods by doing that.” She advocates that programs “should contemplate giving mixed methods just as much respect as we give qualitative and quantitative research,” suggesting that mastery requires more time and sustained engagement. This perspective reflects her context-sensitivity to curricular design and institutional priorities. For her, teaching mixed methods research is not merely about transmitting content, but about transforming structures that limit the depth of student learning.

Ultimately, her narrative portrays a teacher who moves fluidly between structure and sensitivity, rigor and empathy. She teaches not only the techniques of integration, but also the disposition to dwell within complexity. “I want them to be able to do a good mixed methods study,” she reflected, “because many of them go on to do a dissertation that they call mixed methods.” Through patience, presence, and practical wisdom, she helps her students move from mimicry to mastery. Her teaching becomes a form of methodological enactment—context-sensitive, adaptive, and dialogic—rooted in the belief that both research and pedagogy are most powerful when they are responsive to the lived realities in which they unfold.

This *context-sensitive profile* portrays a pedagogue of responsiveness—what Schön (2017) described as the *reflective practitioner*—who continually navigates the tensions between structure and flexibility, rigor and empathy, and institutional constraints and creative adaptation. Her practice demonstrates methodological reflexivity, addressing student diversity, compressed instructional formats, ethical continuity, and disciplinary translation. Through such responsiveness, her pedagogy enacts *contextual pragmatism*, a stance in which meaning and method coevolve within specific situational realities. Beyond enriching the analytic model, her narrative humanizes it, revealing the ethical and affective dimensions of teaching that conventional typologies often overlook. Her attentiveness to care, time, and communication situates mixed methods research pedagogy as both a technical and moral enterprise—bridging analytic abstraction and lived experience, and illustrating how theory becomes embodied in the act of teaching.

### Methodological positioning of the narrative profiling process

The narrative profiling process—which *substantively extends and deepens* the qualitizing framework articulated by Onwuegbuzie and Leech (2019)—illustrates how the data transformation procedures in Onwuegbuzie et al. (2011) could evolve into a multi-stage qualitizing sequence. The original study began with qualitative data (i.e., semi-structured interviews; *Element 2*) that yielded multiple representations (*Element 1*). These were analyzed qualitatively via constant comparison analysis (*Element 3*), producing *metathemes and themes* that then were quantitized and subjected to correspondence analyses (i.e., quantitative analyses; *Element 3*). The resulting *modal pedagogical profiles* (*Element 1*) then could be re-qualitized through the process of narrative profiling to produce interpretive portraits—such as the context-sensitive profile for Participant 2—that collectively exemplify a fully integrated analysis (*Element 5*). This recursive movement from qualitative to quantitative and back to narrative interpretation exemplifies the cyclical and reflexive nature of qualitizing, wherein data transformation progresses toward increasingly human-centered and contextually enriched meta-inferences.

### Scalability and adaptability of narrative profiles

Although the narrative presented in Box 2 represents the optimal level of analytic, contextual, and interpretive richness for the *context-sensitive profile*, it is important to acknowledge that many researchers operate under strict page and word limitations. In such cases—particularly when multiple participant profiles must be reported—it might not be feasible to present full-length narratives of this scope. To demonstrate the scalability of the 43-profile meta-framework, Box 3, 4 present two more compact versions of the same profile: a two-paragraph form and a three-paragraph form.

BOX 3 **Two-paragraph condensed context-sensitive narrative profile.**

Participant 2’s teaching philosophy is grounded in responsiveness and reflexivity—a continual recalibration shaped by the contexts in which learning unfolds. She balances structure with openness, precision with dialogue, transforming the constraints of a three-weekend course into a dynamic environment that fosters continuity through short quizzes, experiential projects, and sustained mentorship. Reflecting on earlier rigidity—“I wanted everyone to think the way I thought”—she now invites multiple perspectives, telling students that “the field is still emerging.” Her approach mirrors the epistemological stance of mixed methods inquiry itself: integrative, adaptive, and dialogic. Her classroom, populated by students from “multiple schools within the university,” becomes a collaborative space of translation, where she constructs a shared language to bridge disciplinary and methodological divides.

Ethics and care form the backbone of her pedagogy. Drawing on her experience with the Institutional Review Board, she weaves ethical reflection throughout lessons, teaching that ethics should be “an ongoing practice.” She maintains constant accessibility—“I’m available by email 24 h a day”—and uses informal class moments to “check in with everybody.” These gestures transform structure into relationship and compliance into moral mindfulness. Finally, she challenges structural constraints in doctoral education, arguing that “we are short-changing mixed methods by doing that” when it is confined to a single course. Her narrative portrays an educator who enacts the essence of the Context-Sensitive Profile: pedagogy as an adaptive, ethical, and human-centered practice responsive to the complexity of lived contexts.

BOX 4 **Three-paragraph condensed context-sensitive narrative profile.**

Participant 2’s teaching philosophy reflects responsiveness, reflexivity, and contextual awareness—a continual recalibration between structure and openness, rigor and empathy. She adapts her methods to institutional, temporal, and relational realities, transforming the constraints of a three-weekend course into opportunities for deeper learning. Reflecting on her early rigidity—“I wasn’t open to talking with them”—she now embraces dialogical flexibility, encouraging students to see that “the field is still emerging.” This stance embodies the integrative ethos of mixed methods inquiry itself: pluralistic, evolving, and grounded in reflection.

Her pedagogy is deeply attuned to student diversity and disciplinary difference. With students drawn from “multiple schools within the university,” she constructs a shared methodological language, bridging varying research traditions and comfort levels. She integrates experiential learning—“They have to do their own mixed methods study”—allowing students to test ideas through action, discovery, and self-assessment. Her quizzes and reflective activities sustain engagement between sessions, turning a compressed delivery format into a rhythm of reinforcement and reconnection.

Ethics and care permeate her approach. Drawing on Institutional Review Board experience, she embeds ethical reflection throughout, ensuring that “students and most researchers think through the ethics of research on an ongoing basis.” Her constant availability—“I’m available by email 24 h a day”—and personal check-ins foster individualized support, transforming logistical structure into human connection. Finally, she critiques institutional limits that “short-change mixed methods,” advocating for its equal curricular standing alongside qualitative and quantitative inquiry. Collectively, her narrative exemplifies the Context-Sensitive Profile: pedagogy as an adaptive, ethical, and relational practice—alive to context, complexity, and the transformative potential of integration.

The *two-paragraph version* (Box 3) represents the *minimum sufficient form* of the narrative profile. It preserves conceptual and ethical fidelity and is ideal when space is constrained but interpretive integrity must be maintained. In contrast, the *three-paragraph version* (Box 4) represents the *ideal minimum*—the shortest structure that still fully expresses the multidimensional essence of the *context-sensitive profile* without sacrificing contextual, relational, or ethical depth.

An ultra-condensed *one-paragraph version* of this same profile can be developed for use in typology tables, appendices, or meta-syntheses; however, it functions as an *analytic précis* rather than as a fully representational narrative profile and, therefore, is not included here. If Onwuegbuzie et al. (2011) had presented narrative profiles of two paragraphs each for the eight participants in their study, this would have produced approximately 2,800 words (95% CI = 2,200–3,400)—equivalent to about 5 double-spaced pages (95% CI = 3.5–6.5)—plus a short synthesis paragraph summarizing interpretive patterns across profiles. Although this expansion would add several pages, the interpretive benefits substantially would outweigh the costs: Each two-paragraph profile transforms abstract analytic findings into human-centered representations that illuminate contextual nuance, participant agency, and methodological reflexivity. In effect, a few thousand additional words yield an exponential gain in interpretive transparency, coherence, and reader comprehension—an excellent cost–benefit ratio for mixed methods research reporting.

Taken together, Box 3, 4 demonstrate that the narrative profiling approach is scalable, adaptable, and philosophically coherent—a clear reflection of the pluralistic ethos of CDP 2.0, which enables researchers to tailor representational form to contextual and pragmatic realities while maintaining methodological rigor and depth even under publication constraints. Having demonstrated the representational flexibility of narrative profiles across publication contexts, the next section outlines their interpretive and methodological value—specifically, how integrating such profiles could have deepened the analytic and pedagogical contributions of Onwuegbuzie et al.’s (2011) study.

## Extending narrative profiling to community-level analyses

Up to this point, the illustration of the narrative profiling process has focused on a *within-case* (individual-level) example—specifically, the context-sensitive profile for Participant 2. However, the same logic of narrative transformation can be extended beyond single cases to analyze patterns and meanings across *groups* of participants. This next analytic tier comprises two interrelated forms: *Within-Community Narrative Profile Analyses* and *Cross-Community Narrative Profile Analyses*. Together, these levels enable researchers to move from *individual voice* to *collective storyline*, and from *within-group coherence* to *between-group comparison*.

### Within-community narrative profile analyses

Within-community analyses examine how meaning, identity, and practice coalesce among members of a single community (e.g., instructors within one program, department, or cultural group). In the context of Onwuegbuzie et al. (2011), the eight mixed methods research instructors could collectively serve as such a community, allowing exploration of how their pedagogical philosophies, constraints, and reflexivities interact to form shared patterns of teaching identity.

Table 5 presents the *Within-Community Narrative Profile Meta-Syntheses*—representing 14 analytic types. These analyses focus on *pattern integration*—that is, identifying recurrent structures and conceptual linkages across individual narrative profiles within a community. Examples include *thematic integration analysis* (consolidating shared pedagogical themes), *relational–resilience mapping* (tracing how collegial or mentoring relationships support adaptive teaching), and *identity positioning analysis* (revealing how instructors negotiate power or belonging within institutional contexts). Each analytic type produces integrative outputs such as community thematic matrices or reflexivity frameworks.

**Table 5:** Within-community narrative profile meta-syntheses (14 analytic types).

Type of meta-synthesis	Analytic focus / purpose	Core analytic operations	Potential outputs / outcomes	Example of application
1. Thematic integration analysis	Identify and consolidate core themes and conceptual anchors across community narratives.	Inductive coding, pattern clustering, cross-profile comparison.	*Community Thematic Matrix* showing dominant and counter-themes.	Cross-case integration in education or healthcare.
2. Relational–resilience mapping	Examine how relationships sustain adaptive processes within the community.	Map relational ties and adaptive responses.	*Relational–Resilience Topography.*	Community development or support networks.
3. Comparative narrative profiling	Identify archetypal differences within the same community (e.g., mentor vs. learner).	Cross-case typological analysis.	*Comparative Typology Table.*	Teacher or leadership identity research.
4. Narrative coherence analysis	Explore how members construct coherence, closure, and meaning continuity in their stories.	Temporal sequencing and coherence mapping.	*Narrative Coherence Continuum.*	Psychological resilience or reflective identity work.
5. Narrative pattern analysis	Analyze recurrent narrative structures, motifs, archetypes, and plot configurations across profiles.	Map structural archetypes (journey, struggle, transformation); compare story grammars.	*Narrative Pattern Matrix* showing common storylines or motifs.	Educational or leadership storytelling research.
6. Symbolic ecology analysis	Identify metaphors and symbols that organize meaning and culture.	Metaphor analysis, symbolic coding.	*Symbolic Landscape Map.*	Cultural or organizational storytelling.
7. Identity positioning analysis	Reveal how speakers position themselves and others within power and belonging structures.	Positioning theory, discourse analysis.	*Identity Positioning Matrix.*	Professional identity or gender discourse studies.
8. Linguistic and discourse analysis	Examine the micro-linguistic features of narrative—word choice, tone, and rhetorical framing—to uncover implicit meaning and ideology.	Discourse and linguistic coding; attention to register, pronouns, evaluative language.	*Linguistic–Rhetorical Profile* capturing narrative voice and ideology.	Organizational communication, policy narrative, or media studies.
9. Affective–emotional dynamics analysis	Trace emotional trajectories, affective intensity, and collective sentiment across community narratives.	Emotional coding, affective sequencing, tone mapping.	*Affective Energy Map.*	Leadership, caregiving, or motivation research.
10. Temporal trajectory analysis	Examine narrative progression and life-course continuity.	Chronological coding, temporal mapping.	*Temporal Trajectory Chart.*	Lifespan development or career trajectory studies.
11. Spatial–contextual analysis	Analyze the role of place, environment, and setting in meaning construction.	Place-based coding; spatial narrative mapping.	*Spatial Context Map.*	Migration, urban education, or digital ethnography.
12. Cognitive–sensemaking analysis	Explore mental models and interpretive logics underlying community reasoning.	Cognitive metaphor and schema analysis.	*Sensemaking Schema Diagram.*	Organizational learning or cross-disciplinary inquiry.
13. Meta-reflexive synthesis	Integrate diverse insights into a reflexive understanding of community transformation.	Cross-analysis and conceptual integration.	*Reflexivity Framework.*	Professional learning or participatory inquiry.
14. Power and ideology analysis	Examine how discourse and structure reproduce or challenge hierarchies.	Critical discourse analysis.	*Ideological Field Map.*	Critical pedagogy or institutional narratives.

Meta-Syntheses (Level 1) focus on analytic integration—what patterns exist across profiles.

Table 5 exemplifies the *within-community narrative profile meta-synthesis: thematic integration analysis*, selected as the most suitable analytic type for the Onwuegbuzie et al. (2011) interviews because it captures how the teaching philosophies of the eight instructors coalesced into shared pedagogical patterns. This analysis integrates discrete thematic clusters—such as *epistemic reflexivity*, *modeling of inquiry*, and *the balancing of methodological instruction with philosophical orientation*—into a coherent structural synthesis. It reveals that within the community of mixed methods research instructors, meaning-making arises through thematic convergence: The instructors’ collective identity emerges not from uniformity but from patterned resonance across distinctive voices. Thus, Box 5 illustrates how thematic integration functions as a meaning-centered act of qualitizing, transforming dispersed insights into a unified interpretive structure.

BOX 5 **Within-community narrative profile meta-synthesis: thematic integration analysis: narrative write-up.**

Across the eight instructors interviewed in Onwuegbuzie et al. (2011) study, a coherent narrative emerges—one that reflects a shared struggle and evolution in shaping what it means to teach mixed methods research during its formative era. Despite diverse institutional contexts, modalities (e.g., online, weekend, or semester-long formats), and disciplinary homes, the instructors collectively demonstrate a unifying commitment: to help students move beyond *methodological dichotomies* toward *integrated research thinking*.

A central thematic pattern is ***scaffolding conceptual clarity amid epistemic diversity***. Instructors repeatedly emphasize the need to establish a common language of mixed methods research—teaching that integration is not a casual blending but a *philosophically grounded process* governed by specific assumptions, logics of inquiry, and legitimation criteria. Whether drawing from the “13-step model” or explicitly juxtaposing the Johnson and Onwuegbuzie (2004) definition with evolving alternatives, each instructor underscores the foundational importance of *terminological precision* and *epistemological transparency* in the classroom.

The second integrative theme concerns ***responding to learner variability through adaptive pedagogy***. Instructors consistently report that mixed methods research classrooms are heterogeneous spaces—composed of students with uneven quantitative and qualitative preparation, disciplinary vocabularies, and analytic competencies. Consequently, these educators design flexible teaching strategies: pairing theory with experiential learning, balancing descriptive-level analyses with thematic coding, and using typologies or real datasets to anchor abstract concepts in practice. This adaptability reflects not only pedagogical pragmatism, but also a deeper *context-sensitive ethos*—a hallmark of effective mixed methods research pedagogy.

A third unifying theme involves ***modeling integration as reflexive practice***. The instructors view teaching itself as an enactment of integration—blending structure and openness, rigor and empathy, analytic formality and narrative engagement. They transform constraints (e.g., limited contact hours, diverse cohorts, or online formats) into opportunities for creative pedagogy. Their courses serve as microcosms of mixed methods inquiry, wherein students learn *by doing*—collecting, analyzing, and reflecting on quantitative and qualitative data in tandem. This recursive teaching process—mirroring the qualitizing–quantitizing–requalitizing cycle—helps students internalize integration as both methodological and ethical practice.

Finally, a metatheme of **emergent professional identity** runs through the narratives. Collectively, these instructors recognize themselves as *first-generation architects* of mixed methods research pedagogy, consciously building a field still defining its contours. They position their teaching not merely as skill transmission, but as identity cultivation—guiding students, and themselves, toward becoming reflexive, pluralistic scholars capable of bridging paradigmatic divides. Their shared story is one of co-constructed learning, wherein both teacher and student participate in the evolving narrative of what it means to “think mixed.”

*Interpretive summary*

This thematic integration reveals a *community identity* grounded in four interlocking commitments:

1. Conceptual clarity and philosophical grounding;
2. Adaptive pedagogy responsive to learner diversity;
3. Reflexive modeling of integration as a teaching act; and
4. Co-creation of a professional identity for mixed methods instructors.

Together, these themes depict a within-community meta-synthesis of **pedagogical reflexivity-in-action**—a collective striving to teach not only the *methods of mixing*, but also the *mindset of integration*.

Bold text denotes themes, the final bolded entry denotes the meta-theme that integrates the preceding themes into an overarching interpretive synthesis.

Table 6 shows the *Within-Community Meta-Narrative Profile Syntheses*—representing 14 interpretive types. These represent the second, interpretive tier, moving from *pattern integration* to *meaning integration*. For instance, a *Meta-Narrative of Practice Pluralism* could capture how diverse instructional styles coexist as complementary strengths, whereas a *Meta-Narrative of Relational Resilience* might depict the collective ethic of care and collaboration uniting instructors across institutional boundaries.

**Table 6:** Within-community meta-narrative profile syntheses (14 interpretive types).

**Type of meta-narrative synthesis**	**Interpretive aim / function**	**Core integrative logic**	**Resulting meta-narrative form**	**Example of application**
1. Meta-narrative of thematic convergence	To weave recurring themes across individual narratives into one coherent, shared storyline reflecting the community’s collective understanding.	Synthesizes major and minor themes from thematic integration analyses to articulate a unified sense of meaning.	*Community Meaning Narrative* portraying shared identity, values, and aspirations.	Used in professional learning communities or team identity studies.
2. Meta-narrative of relational resilience	To portray the community’s capacity for connection, support, and renewal as a source of strength and continuity.	Integrates relational–resilience mapping and affective insights into an overarching storyline of interdependence.	*Collective Resilience Narrative* emphasizing cooperation and mutual care.	Applied in healthcare, education, or social services research.
3. Meta-narrative of practice pluralism	To express how diverse roles, voices, and practices coexist within the same community, forming a pluralistic culture of learning.	Synthesizes typological and comparative profiles to celebrate multiplicity as a resource for growth.	*Dialogic Meta-Narrative* of inclusion and adaptive collaboration.	Used in interdisciplinary or multicultural community studies.
4. Meta-narrative of narrative coherence	To represent how individuals and groups strive to maintain continuity and coherence in their lived stories.	Integrates findings from coherence analyses and temporal trajectories to reveal narrative integration.	*Meta-Narrative of Wholeness* that highlights reconciliation between past and present identities.	Applied in identity formation, recovery, or organizational continuity studies.
5. Meta-narrative of archetypal storylines	To identify and to interpret the recurring story patterns and archetypal plots that define the community’s shared worldview.	Builds on narrative pattern analysis to reveal dominant narrative grammars (e.g., quest, transformation, rebirth).	*Archetypal Story Narrative* presenting collective identity as a repeating journey or cycle.	Used in leadership development, life history, or creative practice studies.
6. Meta-narrative of symbolic ecology	To interpret the metaphors and symbols that sustain collective meaning and cultural identity.	Integrates symbolic ecology findings with thematic and cognitive analyses to portray shared cultural imagery.	*Cultural–Semiotic Narrative* describing how symbols bind the community’s worldview.	Used in cultural anthropology or organizational storytelling.
7. Meta-narrative of identity evolution	To capture how members reconstruct personal and collective identities over time.	Synthesizes identity positioning and temporal analyses to depict transformation, role shifts, and maturation.	*Narrative of Identity Renewal* describing self-redefinition within changing contexts.	Used in teacher identity, professional development, or migration studies.
8. Meta-narrative of discursive voice and power	To interpret how linguistic and rhetorical patterns construct community voice, authority, and agency.	Integrates linguistic and discourse analyses with power–ideology insights to reveal how language encodes inclusion, dominance, or resistance.	*Meta-Narrative of Discursive Agency* showing how collective voice emerges through power negotiation.	Used in critical discourse, organizational communication, or equity studies.
9. Meta-narrative of emotional integration	To portray emotional life as central to the community’s cohesion and meaning-making.	Synthesizes affective–emotional dynamics with relational analyses to represent emotion as connective tissue.	*Affective Meta-Narrative* describing empathy, care, and shared feeling as forces of unity.	Used in leadership, nursing, or counseling research.
10. Meta-narrative of temporal continuity and change	To interpret how the community’s identity evolves through temporal phases of growth, disruption, and renewal.	Builds on temporal trajectory and coherence syntheses to show continuity through adaptation.	*Temporal Meta-Narrative* charting transformation across historical or developmental time.	Used in longitudinal, institutional, or intergenerational studies.
11. Meta-narrative of spatial belonging	To represent how place, environment, and spatial experience shape communal meaning and identity.	Integrates spatial–contextual and symbolic analyses into a storyline of emplacement and belonging.	*Situated Belonging Narrative* connecting geography, space, and sense of home.	Used in migration, community geography, or digital ethnography research.
12. Meta-narrative of cognitive transformation	To capture collective learning, insight, and paradigm shifts within the community’s evolution of thought.	Synthesizes cognitive–sensemaking and reflexive analyses to represent changes in understanding or worldview.	*Learning Community Meta-Narrative* showing intellectual and epistemic growth.	Used in educational innovation or organizational learning studies.
13. Meta-narrative of reflexive praxis	To depict the community’s collective self-awareness and capacity for reflective transformation.	Integrates meta-reflexive synthesis and thematic insights into a narrative of iterative self-renewal.	*Reflexive Praxis Narrative* emphasizing learning through reflection and adaptation.	Used in participatory, professional, or action research contexts.
14. Meta-narrative of ideological negotiation	To articulate how communities grapple with structures of power, belief, and ideology in shaping shared meaning.	Synthesizes power–ideology and discourse analyses into a story of contestation and transformation.	*Transformative Ideological Narrative* representing empowerment, resistance, or reformation.	Used in critical pedagogy, decolonial, or institutional reform studies.

Box 6 represents the *Meta-Narrative of Pedagogical Identity Evolution*, the interpretive complement to the previous thematic analysis. This approach is most pertinent to the Onwuegbuzie et al. (2011) data because the instructors’ accounts collectively chart an identity trajectory—from tentative methodological explorers to confident, reflexive pedagogues constructing an emergent discipline. The meta-narrative synthesis weaves these developmental arcs into a shared storyline of transformation, illuminating how early uncertainty gave way to integrative teaching philosophies grounded in experiential learning and epistemic negotiation. As shown in Box 6, this narrative lens converts individual reflections into a collective chronicle of pedagogical becoming, demonstrating qualitizing’s capacity to portray evolution, not merely aggregation, within interpretive communities.

BOX 6 **Within-community meta-narrative profile synthesis: meta-narrative of pedagogical identity evolution: narrative write-up.**

At the heart of this community of eight first-generation mixed methods research instructors lies a shared journey—a *pedagogical evolution* from methodological uncertainty toward integrative reflexivity. Each participant entered the instructional space amid disciplinary fragmentation, drawing from divergent traditions of quantitative precision and qualitative depth. Yet through teaching, they came to embody a new identity: the *reflexive integrator*, one who not only teaches mixed methods research but *lives* its dialectic in practice.

Early in this narrative arc, instructors positioned themselves as *translators* among paradigms, seeking to demystify the “language of mixing” for students bewildered by methodological divides. For instance, one instructor emphasized grounding students in a conceptual framework with explicit attention to assumptions, definitions, and credible data interpretation, revealing an awareness that integration must begin with epistemic literacy. Similarly, another instructor reflected on the evolution from prescriptive instruction to dialogical openness—no longer insisting that students adopt a single authoritative definition but guiding them to recognize the multiplicity of legitimate frameworks within a still-emerging field. These accounts mark the first stage of pedagogical identity evolution: *from teaching methods to teaching meaning*.

The next phase of development is defined by *adaptive pedagogy and reflexive modeling*. As instructors grappled with heterogeneous student backgrounds, they discovered that teaching mixed methods research required not only conveying knowledge, but also constructing the very conditions for integrative learning. Several instructors described tailoring assignments, scaffolding analytical complexity, and transforming structural limitations—such as compressed weekend courses or online settings—into catalysts for creativity and dialogue. In so doing, they enacted what is termed within the 43-profile meta-framework as *contextual pragmatism*: the capacity to translate methodological pluralism into responsive, situated pedagogy.

As their experience deepened, these educators’ narratives converged on a metatheme of *identity as praxis*. Teaching mixed methods research became a reflexive mirror of their own philosophical commitments. They described teaching not as the transmission of procedure, but as the enactment of a worldview—one that honors multiplicity, balance, and ethical attentiveness. Their classes became laboratories of integration, wherein students’ methodological struggles mirrored the instructors’ ongoing negotiation of mixed methods research as both field and philosophy. Teaching thus became a site of epistemic inquiry: a continuous process of aligning one’s professional, ethical, and methodological selves.

Finally, across these intertwined stories emerges a *collective self-recognition*: that they are, together, the architects of a new pedagogical paradigm. Their evolving identities exemplify the formation of an interpretive community that both constructs and is constructed by the emergence of mixed methods research as a scholarly tradition. Through iterative cycles of teaching, reflection, and adaptation, they co-author a communal narrative of becoming—one that transforms isolated expertise into a shared ethos of integration.

*Interpretive summary*

This meta-narrative portrays the *collective identity evolution* of first-generation mixed methods research instructors as a story of movement—from methodological fragmentation to integrative reflexivity, from prescriptive teaching to dialogical meaning making, and from individual technique to collective philosophy. Their evolution exemplifies the essence of Qualitizing Revisited: integration not as the mechanical convergence of data, but as the *narrative convergence of identities*.

Through this synthesis, the eight instructors emerge not as disparate cases but as a *community of reflexive practitioners*, enacting the very logic of mixed methods inquiry in their pedagogy. They model the transformation of knowledge into meaning, of method into story, and of teaching into the living practice of integration itself.

Together, Tables 5 and 6 form the following two-tier hierarchy: *Individual Profiles → Within-Community Meta-Syntheses → Within-Community Meta-Narrative Syntheses → Cross-Community Frameworks.*

Applied to Onwuegbuzie et al.’s (2011) mixed methods research study, one particularly relevant analytic path would be a *thematic integration analysis* (Table 5) combined with a *Meta-Narrative of Identity Evolution* (Table 6). This pairing could reveal, for example, how instructors’ teaching identities collectively evolve toward greater reflexivity and methodological integration. Unfortunately, space precludes an illustration of these analyses here.

### Cross-community narrative profile analyses

Beyond single-community synthesis, *Cross-Community Narrative Profile Analyses* enable comparative interpretation across two or more communities. A *community* can denote any meaningful subgroup, such as gender, ethnicity, disciplinary field, institutional type, or career stage. For example, in Onwuegbuzie et al.’s (2011) study, the four men and four women instructors could serve as two distinct communities, allowing for comparative analyses of gendered orientations toward teaching mixed methods research.

Table 7 depicts the *Cross-Community Narrative Profile Meta-Syntheses*—representing 17 analytic types. These analyses extend from *within-group coherence* to *between-group differentiation*. Key examples include

- *Comparative Thematic Analysis*, identifying convergent and divergent teaching philosophies between men and women instructors;
- *Intersectional Comparative Analysis*, exploring how gender interacts with discipline or institutional context; and
- *Power–Ideology Cross-Analysis*, examining whether structural or discursive hierarchies shape how instructors perceive methodological authority.

**Table 7:** Cross-community narrative profile meta-syntheses (17 analytic types).

**Type of cross-community meta-synthesis**	**Analytic focus / purpose**	**Core analytic operations**	**Potential outputs / outcomes**	**Example of application**
1. Comparative thematic analysis	Identify convergent and divergent themes across multiple communities.	Cross-case coding and comparative theme mapping.	*Cross-Community Thematic Matrix* distinguishing universal vs. context-specific motifs.	Cross-national education reform or identity studies.
2. Cultural–contextual integration analysis	Interpret how cultural and situational contexts shape narrative meaning differently across communities.	Contextual coding, ethnographic integration.	*Cultural–Contextual Integration Map.*	Indigenous–nonindigenous comparative pedagogy.
3. Ecological systems narrative analysis	Situate communities within multilevel ecological systems to reveal cross-scale influences.	Apply ecological frameworks to map interdependencies.	*Ecological Narrative Diagram.*	Multi-sector sustainability or educational ecosystems.
4. Cross-community narrative meta-synthesis	Integrate findings from all communities into an overarching meta-narrative.	Aggregate meta-themes and meta-patterns.	*Grand Narrative Integration Model.*	Large-scale multi-site qualitative synthesis.
5. Intersectional comparative analysis	Explore how intersecting identities (gender, race, class) vary across communities.	Intersectional discourse and comparative identity analysis.	*Intersectional Comparison Grid.*	Global equity or gender justice studies.
6. Transpositional narrative analysis	Examine how stories and symbols migrate across contexts and transform meaning.	Trace adaptation and translation of narrative elements.	*Narrative Transfer Map.*	Cross-cultural innovation or policy translation.
7. Temporal–comparative trajectory analysis	Analyze how communities evolve over time, identifying parallel or divergent temporal arcs.	Longitudinal cross-case comparison.	*Temporal Trajectory Map.*	Comparative institutional change or generational studies.
8. Power–ideology cross-analysis	Reveal how structures of power and ideology shape differences between communities.	Critical discourse and decolonial analysis.	*Cross-Community Power Map.*	Comparative politics or critical education.
9. Symbolic–semiotic cross-analysis	Compare symbolic repertoires and cultural imagery across communities.	Semiotic mapping, metaphor analysis.	*Cross-Cultural Symbolic Atlas.*	Comparative religion or cultural media studies.
10. Symbolic convergence and divergence analysis	Examine how communities’ symbolic systems either align (converge) or conflict (diverge), revealing shared visions or contested imaginaries.	Identify converging metaphors, shared rhetorical visions, and conflicting symbolic codes.	*Symbolic Resonance–Dissonance Map.*	Intercultural collaboration, peacebuilding, or ideological polarization.
11. Relational–network integration analysis	Compare how communities organize relationships, hierarchies, and collaboration.	Social and narrative network analysis.	*Relational Integration Matrix.*	Transnational partnerships or coalition building.
12. Cognitive–epistemic integration analysis	Compare how communities conceptualize and validate knowledge.	Analyze cognitive metaphors, epistemic logics.	*Epistemic Integration Framework.*	Interdisciplinary or indigenous–scientific epistemologies.
13. Narrative function and purpose analysis	Investigate the communicative, performative, or legitimizing functions of community narratives.	Rhetorical, performative, and discourse-functional coding.	*Narrative Function Map* identifying purpose clusters (e.g., advocacy, sensemaking, identity maintenance).	Comparative public policy, leadership, or advocacy research.
14. Trajectory analysis of resilience	Trace how resilience emerges, declines, or transforms across communities and contexts.	Chronological coding of resilience-related storylines; identify adaptive mechanisms.	*Resilience Trajectory Diagram.*	Cross-community post-crisis or post-conflict adaptation research.
15. Narrative polarity and tension analysis	Examine paradoxes, dualities, and tensions that define intergroup dynamics.	Identify binary oppositions and paradoxical motifs (e.g., freedom–security, tradition–innovation).	*Narrative Polarity Matrix.*	Used in sociopolitical polarization, reform, or ideology comparison.
16. Meta-integrative cross-community synthesis	Integrate diverse findings into a unified, reflexive narrative synthesis.	Combine thematic, ecological, symbolic, and epistemic analyses.	*Meta-Integrative Synthesis Model.*	Transdisciplinary or meta-narrative research.
17. Meta-integrative framework construction	Develop a theoretical model that transcends synthesis—constructing a conceptual architecture linking all analytic levels.	Translate cross-community synthesis into a coherent framework or theory.	*Meta-Integrative Framework Diagram.*	Theory-building, methodological synthesis, or design-based research.

Each analysis type moves from within-group coherence to between-group comparison, spanning structural, cultural, ecological, and epistemic dimensions. The framework complements the within-community analyses by extending integration across communities.

Such analyses produce integrative outputs—for example, cross-community thematic matrices, symbolic resonance maps, or meta-integrative frameworks—that synthesize collective differences into coherent comparative narratives.

Box 7 portrays the *Comparative Thematic Analysis* contrasting the four men and four women instructors. This analytic form was selected as the most suitable cross-community synthesis because the original Onwuegbuzie et al. (2011) dataset was naturally stratified by gender, allowing for examination of structural and epistemic tendencies across sub-communities. The comparative mapping reveals that the men instructors tended to emphasize *methodological scaffolding*, *analytic precision*, and *conceptual frameworks*, whereas the women instructors foregrounded relational *pedagogy*, *contextual responsiveness*, and *reflective dialogue*. Yet, both groups converged around the ideal of integration through flexibility and reflexivity. Therefore, Table 8 visualizes differentiation within unity—showing how thematic contrasts across gendered sub-communities enrich the collective understanding of mixed methods research pedagogy.

BOX 7 **Cross-community narrative profile meta-synthesis: comparative thematic analysis: narrative write-up.**

Across both men and women instructor groups, a shared ethos unites their pedagogical work: each conceives teaching mixed methods research as an act of integration—of knowledge systems, identities, and instructional praxis. Yet, within this unity, gendered inflections emerge, shaping how integration is *performed*, *experienced*, and *communicated*.

1. Epistemic framing: structural vs. relational orientation

The male instructors tend to frame mixed methods research pedagogy through *structural and procedural lenses*. Two of them emphasize the importance of definitional clarity, philosophical grounding, and technical frameworks such as the “13-step model” and typologies to scaffold student understanding. Their narratives are marked by an architectural sensibility—seeking to build conceptual frameworks and to standardize language to ensure methodological rigor. For example, another male instructor underscores the necessity of conceptual sequencing and typological grounding, reflecting an orientation toward procedural coherence.

In contrast, the female instructors foreground a *relational and contextual epistemology*. Two instructors view learning as an evolving dialogue between instructor and student, emphasizing responsiveness, empathy, and iterative sensemaking. For one female instructor, teaching in a compressed weekend format, explicitly centers her pedagogy on “getting students to think like mixed researchers” rather than on mastering steps or definitions. Likewise, another female instructor integrates relational scaffolding—adapting activities to diverse student needs and creating “safe interpretive spaces” where uncertainty is reframed as intellectual growth. This relational orientation manifests a gendered form of *epistemic care*, prioritizing meaning co-construction over methodological prescription.

2. Pedagogical emphasis: design logic vs. transformative learning

The male instructors predominantly position themselves as *design mentors*. Their teaching aims to equip students with systematic frameworks to structure inquiry. One instructor’s reliance on typologies and quantitative-qualitative integration tasks exemplifies a rationalist teaching mode that seeks order and transparency. Although being reflexive about early rigidity, another male instructor, nonetheless, focuses on definitional pluralism within structured boundaries, reflecting a cognitive developmental model of instruction.

In contrast, the female instructors enact a *transformative learning* orientation. Two female instructors emphasize cognitive dissonance as being a catalyst for epistemic expansion—using reflective activities, iterative discussion, and dialogical questioning to provoke deeper engagement with methodological pluralism. The narratives of the other two female instructors show teaching as ethical facilitation: creating spaces where students confront disciplinary hierarchies and reconcile their prior identities as quantitative or qualitative thinkers. Their courses are less about transmission and more about transformation—pedagogy as identity work.

3. Reflexivity and voice: cognitive mediation vs. ethical mediationAcross gender lines, all instructors demonstrate reflexivity, but its manifestation differs in tone and focus. The male instructors’ reflexivity often centers on *cognitive mediation*: improving the clarity, sequence, and coherence of instruction to reduce student confusion. One male instructor’s careful calibration of quantitative and qualitative complexity exemplifies this managerial reflexivity. However, the female instructors’ reflexivity extends into *ethical mediation*: attending to the emotional, institutional, and relational dimensions of learning. One female instructor’s sensitivity to students’ epistemic anxieties and another female instructor’s awareness of temporal dislocation in weekend teaching illustrate this affective reflexivity—a deliberate balancing of care and challenge.

4. Integrative stance: model builders vs. meaning weavers

Ultimately, both groups strive toward integration but through distinct pathways. The male instructors emerge as **Model Builders**—constructing frameworks that legitimize mixed methods research as a structured discipline. The female instructors emerge as **Meaning Weavers**—constructing pedagogical relationships that humanize integration as lived experience. The former operationalize integration as design coherence; the latter as dialogical enactment. Together, they constitute a dialectical whole: *methodological architecture meets relational artistry*.

Interpretive summary

This comparative thematic synthesis reveals not a gender divide but a **gender complementarity** within the pedagogy of mixed methods research. The men contribute structural coherence—clarifying frameworks, models, and definitional consistency—whereas the women infuse the process with relational depth, reflexive awareness, and transformative engagement. Their combined contributions embody the very ethos of mixed methods research: *the integration of rigor and resonance, logic and empathy, system and story.*

In the broader narrative of mixed methods research pedagogy, these two orientations—*building* and *weaving*—operate as mutually generative forces. Together, they construct a pluralistic teaching paradigm that balances the technical precision of methodological instruction with the interpretive richness of human experience.

**Table 8:** Cross-community meta-narrative profile syntheses (14 interpretive types).

**Type of meta-narrative synthesis**	**Interpretive aim / Function**	**Core integrative logic**	**Resulting meta-narrative form**	**Example of application**
1. Meta-narrative of comparative identity evolution	To interpret how differing communities evolve in their identity positions relative to one another over time.	Interprets patterns of transformation, convergence, or divergence in identity narratives.	A cross-temporal storyline tracing how each community’s professional, pedagogical, or epistemic identity adapts in dialogue with others.	Comparing male and female instructors’ evolving orientations toward methodological pluralism.
2. Inter-community dialogical synthesis	To integrate multiple community narratives into a shared dialogical space of meaning.	Constructs meta-narratives from intergroup conversations, tensions, and reconciliations.	A dialogic storyline illustrating how communities negotiate shared understanding and mutual recognition.	Synthesizing differing disciplinary communities’ perspectives on mixed methods research pedagogy.
3. Transcultural meta-narrative of integration	To interpret meaning across communities with distinct cultural, epistemological, or institutional foundations.	Integrates symbolic, linguistic, and contextual meanings into a transcultural frame.	A unifying storyline showing how culturally distinct pedagogical practices coalesce into an overarching epistemic pluralism.	Comparing instructors from U. S. and non-U. S. institutions to reveal integrative global pedagogical narratives.
4. Meta-narrative of power reconciliation	To interpret how communities negotiate hierarchy, dominance, or marginalization.	Interprets relational asymmetries and their transformation into equity or inclusion.	A reflexive storyline that portrays the movement from power imbalance toward dialogical reciprocity.	Interpreting differences in perceived methodological authority between senior and junior instructors.
5. Narrative of methodological pluralism	To synthesize how communities embody different yet complementary epistemic stances.	Reinterprets divergences as pluralistic synergies rather than oppositions.	A cohesive meta-narrative showing pluralism as strength through methodological complementarity.	Synthesizing positivist-leaning and constructivist-leaning teaching philosophies.
6. Comparative ethics meta-narrative	To interpret how ethical commitments manifest and diverge across communities.	Integrates moral reasoning and value orientations across cases.	A moral storyline articulating shared and divergent ethical logics in research pedagogy.	Comparing ethical emphases between institutional cultures with differing IRB expectations.
7. Meta-narrative of contextual adaptation	To interpret how varying situational constraints shape communal adaptation and innovation.	Integrates ecological and contextual shifts across cases into interpretive coherence.	A storyline of adaptive evolution showing how communities respond to institutional, temporal, or resource constraints.	Comparing how instructors in large vs. small programs adapt course design to context.
8. Narrative of relational ecology	To interpret interdependence among communities and their ecosystems.	Synthesizes intergroup relationships into an ecological web of mutual influence.	A systemic storyline illustrating how communities co-evolve within a shared pedagogical ecology.	Mapping relational exchanges between departments co-teaching mixed methods research courses.
9. Comparative affective resonance narrative	To interpret emotional and motivational parallels across communities.	Integrates affective tone, empathy, and commitment into intergroup meaning.	A human-centered storyline emphasizing emotional resonance and pedagogical empathy.	Synthesizing instructors’ affective experiences of teaching across institutional settings.
10. Narrative of epistemic convergence and divergence	To interpret how epistemological assumptions align or conflict across groups.	Juxtaposes paradigmatic commitments to reveal deeper integrative possibilities.	A philosophical storyline portraying epistemic synthesis amid difference.	Comparing communities emphasizing pragmatism vs. constructivism in teaching mixed methods research.
11. Cross-community sensemaking synthesis	To interpret how communities collectively construct meaning from shared challenges or innovations.	Integrates narrative sensemaking processes into higher-order coherence.	A metanarrative depicting collective reflection and meaning reconstruction across contexts.	Integrating cross-institutional narratives of how faculty define “mixed methods rigor.”
12. Meta-narrative of resilience and renewal	To interpret how communities recover and evolve through shared adversity or change.	Synthesizes temporal trajectories of challenge, adaptation, and renewal.	A resilience narrative showing transformation and regeneration across communities.	Examining pedagogical renewal following institutional restructuring or crises.
13. Inter-community reflexive integration	To interpret meta-level reflexivity—how communities interpret each other’s interpretations.	Integrates recursive reflection and meta-dialogue across groups.	A layered storyline of mutual reflexivity and learning between interpretive communities.	Analyzing how differing faculties critique and learn from each other’s mixed methods research frameworks.
14. Meta-narrative of transformative pluralism	To interpret overarching integration across communities toward a collective paradigm shift.	Synthesizes multiple narrative layers into a unifying transformative framework.	A culminating storyline of synthesis—showing how plural communities together advance an inclusive epistemological paradigm.	Interpreting how diverse teaching communities collectively reframe mixed methods research pedagogy as transformative praxis.

Cross-Community Meta-Narrative Syntheses (Level 2) focus on interpretive integration—how comparative patterns across communities are transformed into shared or dialogical meaning systems. This tier extends beyond identifying differences to interpreting their collective significance—revealing how diverse communities co-construct, negotiate, and evolve integrative metanarratives that embody the pluralistic, reflexive ethos of Critical Dialectical Pluralism.

Table 8 presents the *Cross-Community Meta-Narrative Profile Syntheses*—representing 14 interpretive types. These interpretive analyses extend the comparative logic of Table 7 from identifying patterns of difference to constructing shared meaning systems across communities. They move beyond analytic contrast to interpretive integration, transforming cross-group patterns into higher-order metanarratives that reveal how diverse communities collectively make sense of, negotiate, and evolve their pedagogical philosophies. Examples include the *Meta-Narrative of Comparative Identity Evolution*, which, in the context of Onwuegbuzie et al.’s (2011) study, traces how differing communities of instructors adapt their professional and epistemic identities in dialogue with one another; the *Transcultural Meta-Narrative of Integration*, which interprets cross-contextual pluralism as a source of methodological enrichment; and the *Meta-Narrative of Transformative Pluralism*, which synthesizes the collective evolution of diverse teaching communities into a shared paradigm of inclusivity and integration.

Box 8 presents the *Meta-Narrative of Relational–Epistemic Alignment*, chosen as the culminating interpretive synthesis because it transcends group differences to narrate how all eight instructors co-constructed a shared epistemic ethos. This synthesis interprets the interplay between relational pedagogy and methodological rigor as a dialectic rather than as a divide: the women instructors’ emphasis on contextual connection and the men instructors’ focus on structural precision mutually informed an integrated philosophy of teaching mixed methods research. Through dialogic negotiation, these educators achieved what is termed in the box as ‘relational–epistemic alignment’—a collective stance that values both human connection and analytic coherence. In this sense, Box 8 embodies the interpretive culmination of qualitizing, transforming comparative contrast into narrative coherence that bridges differences through shared meaning.

BOX 8 **Cross-community meta-narrative profile synthesis: meta-narrative of relational–epistemic alignment: narrative write-up.**

The teaching stories of the eight mixed methods research instructors, when read dialogically across gendered lines, unfold not as parallel narratives but as converging arcs in a shared pedagogical evolution. What initially appears as contrast—structure versus flexibility, framework versus fluidity, cognitive clarity versus relational empathy—reveals itself, on interpretive synthesis, as a system of *reciprocal calibration*. The men and women instructors are not oppositional types but complementary interlocutors in a collective project: aligning the cognitive architecture of mixed methods research instruction with its relational, ethical, and human dimensions.

At one pole of this alignment, the male instructors articulate the **epistemic infrastructure** of mixed methods research pedagogy. Their teaching centers on making integration *visible and teachable*—through models, frameworks, and explicit sequencing of methodological reasoning. One male instructor’s insistence on definitional precision and another male instructors movement from prescriptive to plural definitions demonstrate an enduring belief in *clarity as an ethical responsibility*: students must be equipped to navigate paradigmatic complexity with intellectual discipline. In their classrooms, knowledge takes structural form—ordered, cumulative, and traceable—anchored in the conviction that rigor provides the scaffolding for creative synthesis.

At the complementary pole, the female instructors foreground the **relational infrastructure** of integration. Their pedagogy is not scaffolded by typologies but by dialogue, responsiveness, and reflexivity. For them, integration is not only methodological, but *intersubjective*: a process of cultivating awareness, trust, and self-reflection within the learning community. One female instructor’s focus on safe interpretive spaces and another female instructor’s commitment to adapting instruction to students’ epistemic comfort zones express an ethic of care that is methodological as much as pedagogical. The other two female instructors extend this relational stance to the temporal dimension—recognizing that meaningful integration unfolds through iterative encounters, not singular lessons.

Viewed together, these two pedagogical orientations enact what might be termed a **dialectic of mutual completion**. The *structural intentionality of the male instructors* creates the conditions under which pluralism can be ***understood***; the *relational intentionality of the female instructors* creates the conditions under which pluralism can be ***lived***. Both are indispensable to the pedagogy of mixed methods research, and both converge on the same epistemic horizon: integration as the disciplined cohabitation of difference. This alignment unfolds through three interpretive movements.

First, **reflexive recognition**—both groups independently arrive at the realization that teaching mixed methods research is as much about *becoming* integrative as *teaching* integration. Male instructors achieve this through cognitive reflection on the limits of proceduralism; female instructors through relational reflection on the affective and contextual dimensions of learning.

Second, **dialogic convergence**—their narratives, when read together, reveal a shared commitment to teaching as dialogue: between paradigms, between instructor and student, and between self and method.

Third, **epistemic synthesis**—a collective identity crystallizes, wherein methodological rigor and relational reflexivity cease to be separate virtues and become two dimensions of the same integrative stance. In this synthesis, *rigor becomes relationally grounded*, and *empathy becomes methodologically rigorous*.

The resulting *meta-narrative*—the narrative above the narratives—is one of **relational–epistemic alignment**. It tells how plural teaching identities, shaped by different emphases and experiences, harmonize into a communal pedagogy that mirrors the very logic of mixed methods research: *the pursuit of coherence through dialogic integration*. Instructors, through their diversity, model the philosophical stance that they seek to impart—one that values tension as generative and plurality as strength.

*Interpretive summary*

This meta-narrative reframes gendered differences not as divisions, but as *axes of epistemic complementarity*. The male instructors’ structured epistemology and the female instructors’ relational epistemology together yield a **meta-pedagogy of integration**—a teaching praxis in which analytical rigor and interpretive compassion coalesce. Their convergence exemplifies the highest expression of *CDP 2.0’s dialectical pluralism*: integration achieved not by erasing difference but by sustaining it in constructive dialogue.

Through this relational–epistemic alignment, the community of instructors collectively embodies the philosophical essence of mixed methods inquiry: that understanding emerges at the *intersection of perspectives*, and that coherence is not imposed but *negotiated through relationship.*

Bold text denotes themes, the final bolded entry denotes the meta-theme that integrates the preceding themes into an overarching interpretive synthesis.

As with Tables 5, 6, and 7, the interpretive types presented in Table 8 are not exhaustive but intentionally comprehensive—providing a flexible, evolving meta-framework that invites continual refinement as new forms of narrative inquiry emerge—reflecting CDP metaprinciples that value openness, reflexivity, and methodological pluralism. This interpretive tier completes the two-level community analytic structure by extending meaning integration across groups, transforming comparative insights into collective metanarratives that illuminate how plural communities co-construct, contest, and evolve shared pedagogical and epistemological understandings. This design reflects the generative spirit of qualitizing revisited, emphasizing adaptability rather than closure. By leaving conceptual space for methodological innovation, the meta-framework remains responsive to new epistemological paradigms, disciplinary contexts, and technological developments that continue to reshape the landscape of narrative research. Collectively, these evolving interpretive frameworks ensure that narrative profiling remains a dynamic vehicle for integration—continually renewing the dialogue among method, meaning, and context.

## Arts-based extensions of narrative profiling

In alignment with the pluralistic and reflexive ethos of the 43-profile Narrative Profiling Taxonomy and CDP 2.0’s commitment to integration through representation, both individual and group-level narrative profiles already provide richly layered accounts—philosophical, ethical, relational, contextual, and aesthetic. Yet, these profiles need not remain solely textual or analytic. They can evolve from analytic transformation into aesthetic representation. Because of their multidimensionality, they can be powerfully re-represented through multiple arts-based modalities, each highlighting a different aspect of meaning—affective, sensory, temporal, or relational. For example, the previously documented *context-sensitive profile of Participant 2*, originally articulated as a written narrative, lends itself to multiple arts-based translations, each re-qualitizing meaning through a distinct expressive medium. In what follows are 14 distinct arts-based and performative re-representations, organized by medium and interpretive purpose.

I Literary and poetic forms

1 Poetic representation. Transform the full *context-sensitive profile* into a lyrical, rhyming poem that captures the cadence of teaching as reflective artistry.
  *Interpretive focus*: Expressive integration—translating the narrative into emotional and rhythmic meaning.
  *Purpose*: To humanize and to aestheticize pedagogy through verse, bridging analysis and affect.
  *Possible use*: Publication inset, performance reading, or digital companion piece.
2 Found Poetry Sequence. Extract key lines and phrases from Participant 2’s transcript and arrange them as found poetry in movements such as “Constraint,” “Connection,” and “Continuity.”
  *Interpretive focus*: Language as data—distilling philosophical stance through repetition and cadence.
  *Example*:
*Teaching is rhythm, not schedule.*
*Ethics is melody, not margin.*
3 Epistolary Narrative. Reimagine the profile as a sequence of reflective letters—for example, *Letters to My Students* or *Letters to My Future Self.*
  *Interpretive focus*: Temporality, intimacy, and mentorship across time.
  *Tone*: Warm, dialogic, and contemplative.

II Visual and material forms

4 Illustrated Contextual Portrait. A hand-rendered illustration depicting Participant 2 surrounded by symbolic motifs (e.g., hourglass, scales, yin–yang, or world map).
  *Interpretive focus*: Pedagogy visualized as dynamic equilibrium—integrating ethics, care, and institutional context.
  *Purpose*: To visualize narrative integration in a single symbolic frame.
5 Visual Ethnographic Collage: “Pedagogical Resonance.” A mixed-media collage depicting the four pedagogical dimensions: Ethical Integration, Relational Praxis, Contextual Adaptation, and Reflexive Continuity.
  *Interpretive focus*: Integration as visual layering—meaning emerging from juxtaposition.
  Use: Could serve as a table to show how narrative profiling transcends textual representation.
6 Mandala of Reflexivity (Symbolic Representation). A circular mandala where quadrants represent ethical, relational, contextual, and reflexive teaching domains.
  *Interpretive focus*: Teaching as balance and cyclic renewal.
  *Output*: Digital artwork or animated infographic for visual synthesis.
7 Textile-Based Representation (Pedagogy as Weaving). A woven or embroidered wall piece using interlaced threads to symbolize integration through tension and connection.
  *Interpretive focus*: The metaphor of weaving—structure, flexibility, and unity in difference.
  *Caption*: “Teaching as Tension and Texture.”
8 Photovoice Installation. Gallery-style installation combining images of learning environments with handwritten excerpts of Participant 2’s reflections.
  *Interpretive focus*: Visual storytelling as reflexive pedagogy.
  *Exhibit context*: University atrium or mixed-methods research conference.
9 Illustrated Reflective Journal. A hybrid text-and-image journal incorporating Participant 2’s reflections, sketches, and marginalia.
  *Interpretive focus*: Teaching as a living text—knowledge as iterative inscription.

III Performative and multimedia forms

10 Dramatic Monologue (Performance Art). A solo performance voiced in the first person, blending internal reflection and pedagogical dialogue.
  *Interpretive focus*: Embodied reflexivity—performance as critical self-inquiry.
  *Use*: Conference dramatization or pedagogical theatre.
11 Autoethnodramatic Dialogue. A two-character dialogue between Participant 2 and an imagined counterpart (e.g., *The Structuralist*).
  *Interpretive focus*: CDP as enacted conversation.
  *Purpose*: Demonstrates CDP’s dialogic epistemology through dramatic form.
12 Short Film / Visual Poem. A cinematic rendering of the reflective practitioner’s narrative.
  *Interpretive focus*: Temporal and emotional rhythm in teaching; reflexivity as cinematic flow.
  Form: 8- to 10-min film integrating voice-over, classroom imagery, and poetic narration.
13 Musical Composition / Soundscape: “Pedagogical Resonance.” A chamber or digital sound piece translating teaching rhythms into sonic movement.
  *Interpretive focus*: Translating pedagogical tempo into auditory experience—structure as sound.
  *Structure*: *Constraint (Adagio) → Dialogue (Allegro) → Integration (Andante).*
14 Digital Storytelling / Interactive Web Profile. An interactive digital exhibit that allows users to explore layered narrative content: transcript excerpts, visuals, journal fragments, and video clips.
  *Interpretive focus*: Hypertextual integration—a living, nonlinear expression of revisited qualitative insights.
  *Medium*: Web-based interface for immersive research dissemination.

### Meta-commentary

Each arts-based form enacts a distinct epistemic and aesthetic function, as follows:

- Poetic and musical forms evoke rhythm, empathy, and affective knowing.
- Visual and material forms embody integration through spatial and symbolic design.
- Performative and cinematic forms foreground embodiment, temporality, and relational praxis.
- Digital and interactive forms invite pluralistic engagement and non-linear exploration.

Together, these modes enact the reflexive, dialectical, and pluralistic ethos of CDP 2.0, demonstrating that narrative profiles are not merely analytic representations, but *aesthetic acts of integration*. They render qualitizing as living inquiry—a fusion of method, meaning, and artistry wherein *data become dialogue* and *interpretation becomes creation*.

### Poetic re-representation of narrative profiles

As noted in the previous section, to extend the interpretive potential of qualitizing, both individual and group-level narrative profiles can be represented through poetic form—a mode of expression that captures the emotional, rhythmic, and existential dimensions of meaning often muted in traditional prose. As demonstrated in works on poetic representation in mixed methods research (e.g., Archibald and Onwuegbuzie, 2020; Onwuegbuzie, 2024a, 2024b; Onwuegbuzie et al., 2024; Williams et al., 2018a, 2018b), poetry provides a condensed yet profound vehicle for rendering participants’ lived experiences, voices, and intersubjective connections. Within the context of qualitizing, each narrative profile can be reconceived as a form of research poetry, wherein rhythm, imagery, and repetition embody the structural and affective logic of meaning-making revealed through integration. Individual narrative profiles, when reimagined as poetic monologues or reflexive verses, can encapsulate participants’ positionalities, tensions, and epistemic shifts with an immediacy and resonance that prose seldom achieves. Group-level narrative profiles, in turn, can be crafted as polyvocal poems, interweaving multiple voices, perspectives, and contexts to evoke collective meaning—a choral synthesis that parallels the relational–epistemic alignment revealed through cross-community analyses. In this way, poetry serves as both analytic representation and methodological metaphor, transforming integration into artful inquiry.

This poetic turn in narrative profiling aligns seamlessly with the principles of CDP 2.0 and the broader vision of qualitizing as meaning synthesis. Poetry’s ability to distill complexity through rhythm, metaphor, and juxtaposition mirrors the dialectical processes by which qualitative and quantitative dimensions are unified into coherent understanding. Furthermore, poetic representation enhances accessibility, emotional engagement, and interpretive depth, bridging the intellectual and aesthetic domains of mixed methods inquiry. As argued by Onwuegbuzie (2024a, 2024b), verse can democratize scholarly communication by foregrounding the affective and ethical dimensions of knowledge production. Similarly, narrative profiles expressed as poetry invite readers not only to understand data, but also to feel its significance—to encounter integration as lived experience. Thus, poetic narrative profiling transforms qualitizing into a multisensory, dialogic act that humanizes evidence, celebrates pluralism, and affirms that meaning in research is as much *felt* as it is *found*.

Importantly, one does not need to have written poetry since childhood to engage in this process. As Lahman et al. (2011) observed, researchers can aspire to create what they termed “good enough research poetry” (p. 894), wherein

Good enough poetry gives novice research poets the space to share their work with peers and critics in the hopes of improving and getting closer to the essence of participants’ understandings—something all social science researchers hope for—poet or not. (Lahman and Richard, 2014, p. 894)

An example of such re-representation is presented in Box 9, which reimagines Participant 2’s narrative as a rhymed poetic form that fuses ethical, relational, and pedagogical reflexivity into lyrical expression. This poetic representation demonstrates how data integration can be embodied, affective, and human-centered. It exemplifies how meaning can be conveyed through aesthetic rhythm, imagery, and emotion while retaining analytical precision. This representation—and the other arts-based extensions—illustrates that narrative profiles are not only analytic outputs, but also aesthetic acts of meaning-making, reaffirming qualitizing as a living synthesis of interpretation, creativity, and human understanding.

BOX 9 **Poetic representation of the context-sensitive profile.**

The reflective practitioner in mixed methods research pedagogy

She teaches within compressed time’s art,

Three weekends to weave both head and heart;

In dialogic space, her students find voice,

Between structure and freedom, they learn to make choice.

Her praxis becomes an art of response,

Each silence a dance, each pattern ensconced;

Boundaries become bridges, not bars,

And inquiry unfolds beneath shifting stars.

She mentors through presence, ethics, and care,

Embedding reflection in each question laid bare;

Her pedagogy breathes—adaptive, alive,

In context she roots, yet invites minds to dive.

Method meets meaning in each design,

Where rigor and empathy intertwine;

Ethics are woven, not taught apart,

The moral of method beats through her heart.

Constraints of curriculum spark her reform,

She questions the frame, reshapes the norm;

Through limits, she fashions relational space,

A pedagogy grounded in dialogic grace.

Each learner’s story refines her own,

In plural voices, her insight is grown;

The classroom becomes a site of becoming,

Where method and meaning keep softly drumming.

Thus, she embodies integration’s art,

A context-sensitive teacher, whole yet apart;

Through reflection she teaches, through teaching reflects,

And through each encounter, new knowing connects.

Her practice—responsive, human, precise—

Marries structure and freedom, rigor and spice;

In the rhythm of learning, she finds her creed,

To teach is to listen, to guide, and to heed.

## Integrative utility of community-level narrative profiling

Together, the within-community and cross-community approaches provide a scalable analytic scaffold that bridges micro-level reflexivity and macro-level meaning systems. Within-community analyses highlight how individuals co-construct collective pedagogical identities, while cross-community analyses—including the interpretive syntheses represented in Table 8—reveal how distinct groups negotiate differences, alignment, and transformation. Applied to the mixed methods research pedagogy study, this dual-level framework could transform typological findings, such as orientation, application, and structure, into dynamic interpretive systems that show how communities of instructors collectively embody, negotiate, and evolve mixed methods research philosophy. By explicitly linking the conceptual framework (Tables 5, 6, 7, 8) to applied illustrations (Boxs 5, 6, 7, 8), the article models the full cycle of narrative integration—from analytic patterning to interpretive synthesis—thereby demonstrating the practical enactment of qualitizing revisited. In essence, these community-level narrative analyses elevate qualitizing from a process of integration within cases to one of inter-contextual synthesis—a methodological evolution that remains faithful to the dialectical, reflexive, and pluralistic ethos of CDP 2.0-based mixed methods research.

### Value of using individual and group-level narrative profiles

Including *individual* narrative profiles, such as the context-sensitive profile for Participant 2, would have added substantial value to Onwuegbuzie et al.’s (2011) original study in several key ways. In tandem, the addition of group-level narrative analyses—illustrated through the within-community and cross-community syntheses in Box 5, 6, 7 and 8—further demonstrates how interpretive depth and methodological integration can be achieved across analytic tiers, linking individual stories to collective pedagogical meaning systems.

First, individual and group-level narrative profiles would have humanized and deepened the qualitative findings already present in the study. Although the 2011 article provided rich, systematically coded metathemes, these were presented abstractly through matrices, tables, and correspondence analyses. Including individual narrative profiles would have translated those metathemes into lived experiences that embodied each typology, while group-level profiles would have illuminated shared thematic structures and relational dynamics across participants—contextualizing personal narratives within communal pedagogical patterns. For instance, the “context-sensitive profile” gives readers insight into the situated reflexivity of an instructor negotiating constraints (e.g., a 3-weekend course format), demonstrating the interplay between context and pedagogy. Such narrative contextualization transforms the typology from an analytic model into an experiential understanding—illustrating how theoretical categories manifest in practice.

Second, these profiles would have enhanced the study’s interpretive and pedagogical transferability. The original study was designed around a fully mixed concurrent dominant status design, integrating qualitative and quantitative research strands to build a “three-dimensional model for categorizing and organizing pedagogical approaches” (p. 180). Narrative profiles would have complemented that model by providing *thick description* (Geertz, 1973; Ryle, 1949, 1971) that helps readers see how instructors’ beliefs, institutional settings, and instructional choices coalesce within those dimensions. By reading how an instructor’s teaching evolves in response to disciplinary diversity, compressed timelines, or ethical commitments, future educators more easily could apply lessons to their own contexts. At the group level, cross-community syntheses extend this transferability by showing how shared experiences and divergent perspectives together shape fieldwide pedagogical evolution—thereby offering a model for comparative reflexivity across contexts. This bridges the gap between empirical categorization and pedagogical practice, providing actionable insights for instructors of mixed methods research courses.

Third, such profiles would have amplified the mixed methods research integrity of the study itself. Onwuegbuzie et al. (2011) emphasized critical dialectical pluralism as their guiding philosophical stance, aiming to integrate multiple epistemological perspectives. Narrative profiles would have enacted that philosophy by integrating analytic and narrative modes—quantitative typology and qualitative life story—into a single interpretive product. Each narrative could function as a meta-inference, synthesizing evidence from interviews, reflexive journals, syllabi, and contextual factors. Thus, the inclusion of profiles would not merely add texture but serve as a methodological extension of the study’s own mixed methods inquiry stance. Group-level narrative syntheses reinforce this dialectical pluralism by enacting integration across cases, demonstrating how diverse voices form higher-order meaning systems that embody the very logic of dialectical inquiry.

Fourth, including these profiles would have made the findings more accessible and engaging to a broader scholarly and practitioner audience. Narrative profiles would have allowed readers to connect emotionally and intuitively with participants’ experiences. For instance, readers could see how a *reflective practitioner* internalizes methodological pluralism, negotiates ethical dilemmas, or scaffolds diverse learners—making the findings more memorable and pedagogically instructive. When extended to the community level, narrative syntheses invite readers to recognize themselves within shared professional stories, enhancing empathy and collective identification with the evolving pedagogy of mixed methods research.

Finally, the inclusion of individual and group-level narrative profiles would have contributed to theoretical refinement within the field of mixed methods research pedagogy. Each profile could serve as an exemplar of a pedagogical archetype within the three-dimensional framework (e.g., a context-sensitive instructor exemplifying adaptive, integrative, and ethically embedded practice). This would allow readers to trace how abstract typological constructs are embodied in real instructors’ epistemologies and teaching behaviors. Over time, such profiles could inform typological expansion, giving rise to richer taxonomies that bridge micro-level (individual) and macro-level (institutional) pedagogical contexts. In particular, the group-level analyses shown in Boxs 5, 6, 7 and 8 illustrate how interpretive integration can move beyond aggregation to synthesis, transforming local insights into systemic narrative understanding.

Moreover, by extending narrative profiling beyond individual cases to community-level analyses—through the within-community and cross-community syntheses represented in Tables 5, 6, 7 and 8—researchers could have derived even greater interpretive value. These higher-order analyses would have revealed not only individual pedagogical identities, but also the collective narratives, relational dynamics, and meta-patterns that define mixed methods research pedagogy as a shared, evolving practice. Integrating such multi-tiered narrative syntheses would have transformed the study from a typological description into a systemic narrative of community sensemaking—linking individual reflexivity with communal and cross-contextual meaning structures.

In essence, individual narrative profiles like the context-sensitive profile, as well as group-level narrative profiles, would have transformed the 2011 study from a primarily analytic mapping of pedagogical approaches into a lived cartography of mixed methods research teaching. They would have animated the study’s three-dimensional framework with human voice and contextual nuance—bringing into focus the dialogic, evolving, and situational nature of teaching mixed methods research. By explicitly connecting micro-level profiles with community-level syntheses (Box 5, 6, 7, 8), the study would demonstrate qualitizing as a recursive and systemic representational process—moving fluidly among personal story, shared identity, and collective meaning. Extending these profiles into community-level syntheses amplifies this transformation, demonstrating how qualitizing revisited operates not only within cases, but also across communities of inquiry. Therefore, the revisiting of the Onwuegbuzie et al. (2011) study demonstrates that narrative profiling provides a next-generation extension of qualitizing—one that not only converts qualitative data into quantifiable dimensions, but also re-qualitizes quantitative and typological outcomes into storied meaning. As such, the integration of the 43-profile meta-framework situates qualitizing as a cyclical and reflexive process, advancing beyond analytic integration toward narrative integration, wherein the human story becomes both data and meta-inference.

### Cross-referencing to empirical data sets

The conceptual sophistication of the 43-profile taxonomy finds its fullest expression when applied to empirical data. Although this taxonomy provides a comprehensive framework for qualitizing, its epistemic strength lies in its ability to be operationalized across diverse research designs and data structures. Cross-referencing the narrative profile types with empirical datasets enables researchers to move from theoretical mapping to analytic enactment, demonstrating how each profile functions as a distinct but interrelated pathway from data to meaning. Through such empirical correspondence, narrative profiling is no longer merely a representational innovation—it becomes a dynamic method for generating, organizing, and interpreting knowledge within actual research contexts.

Empirical cross-referencing involves a deliberate alignment between the *logic of the narrative profile* and the *structure of the dataset*. In quantitative contexts, this may entail identifying the analytic forms—such as clusters, trajectories, or latent dimensions—that correspond to specific narrative profiles and constructing story-based representations that articulate these statistical patterns in interpretive terms. In qualitative or mixed datasets, the process operates reciprocally: Emergent narratives, cases, or thematic clusters can be examined to determine which narrative profile type best encapsulates their structural and interpretive properties. In both directions, the goal is not to force data into predefined categories but to reveal the narrative potential inherent within the data forms themselves. Cross-referencing thus functions as an interpretive act of calibration—testing the fit between data patterns and narrative forms to ensure both conceptual integrity and representational fidelity.

In practice, cross-referencing can be achieved through three interrelated analytic procedures. First, *profilic mapping* involves systematically identifying where within a dataset specific analytic operations align with one or more narrative profile types (e.g., identifying a *trajectory* pattern in longitudinal survey data or an *interactional* profile within conversational transcripts). Third, *profilic legitimation* involves iterative triangulation between the narrative representation and the original data, assessing whether the narrative profile accurately reflects empirical tendencies and interpretive depth. Together, these procedures instantiate the full cycle of qualitizing as a method of empirical reasoning: discovery, integration, and verification through narrative form.

Cross-referencing also enhances *comparability* and *reproducibility* in mixed methods research. By linking each narrative profile type to concrete empirical operations—such as regression models, thematic networks, or case typologies—researchers can make transparent how qualitative and quantitative evidence converge within a unified interpretive schema. This alignment enables meta-analytic reasoning across studies, facilitating comparative synthesis of qualitizing practices. For instance, one study’s use of a *resilience profile* in health psychology and another’s use of a *trajectory profile* in educational research can be cross-referenced analytically, revealing shared narrative logics of adaptation and transformation despite disciplinary or methodological differences. In this way, the 43-profile taxonomy functions as a *transdisciplinary meta-language*—a common interpretive grammar through which empirical diversity can be systematically integrated and compared.

At a deeper level, cross-referencing empirical data sets positions the researcher as both *analyst* and *narrator*. The process emphasizes interpretive judgment, requiring the researcher to decide which profile or combination of profiles most accurately represents the data’s underlying structure and experiential meaning. To support researchers in engaging in this reflexive decision-making process, it is useful to articulate a set of guiding criteria that can inform the selection and construction of narrative profiles. First, researchers should consider the *analytic purpose* of the study—whether the goal is to describe central tendencies, to identify patterns, to examine relationships, to contextualize experiences, or to generate transformative insights—because different purposes align with different profile types. Second, attention should be given to the *structure and characteristics of the data*, including whether the dataset is primarily quantitative, qualitative, or mixed, as well as whether it reflects within-case depth or cross-case variation. Third, researchers should reflect on the *level of integration sought*, distinguishing between individual-level representation and group-level synthesis, with the latter typically requiring profiles that capture convergence, divergence, or relational patterns across cases. Fourth, the *epistemic orientation and theoretical framework* guiding the study should inform profile selection, ensuring alignment between narrative form and underlying assumptions about knowledge, meaning, and representation. Finally, reflexive consideration of *ethical and representational implications* is essential, including how narrative choices may amplify or obscure participant voices, contextual nuance, or power dynamics. Together, these criteria position reflexive decision-making not as an intuitive or *ad hoc* process, but as a structured and transparent component of narrative profiling that enhances both analytic rigor and interpretive coherence. This reflexive decision-making process ensures that qualitative depth and quantitative precision are preserved within the act of integration. Moreover, it reinforces the epistemological principle that data do not carry inherent meaning but acquire it through interpretive narration. The act of cross-referencing thus becomes a site of epistemic transparency—where the interpretive moves of the researcher are visible, traceable, and accountable within the analytic record.

Crucially, empirical cross-referencing also serves a *legitimation-enhancing function*. By testing narrative profiles against actual data structures, researchers can assess whether the chosen representational form exaggerates, diminishes, or accurately mirrors empirical relationships. Profiles that fail to align with the empirical contours of the data invite revision or hybridization, ensuring that the taxonomy remains flexible and empirically grounded. In this way, cross-referencing is both confirmatory and generative: It legitimizes existing typologies while also allowing for the emergence of new, hybrid narrative profiles responsive to specific empirical conditions. Over time, this process contributes to the *evolutionary refinement of the taxonomy itself*, keeping the framework dynamic, data-responsive, and methodologically progressive.

Finally, cross-referencing empirical datasets underscores the *bidirectional flow between theory and data* that defines qualitizing as a mode of mixed methods research reasoning. The taxonomy offers a conceptual map of narrative integration; empirical data provide the terrain through which that map is continually tested, revised, and enriched. Through their intersection, the representational and the empirical become mutually constitutive—each shaping the other in an ongoing dialogue between meaning and evidence. This reciprocal relationship transforms narrative profiling from a static classificatory device into an evolving methodological ecology: one that adapts to the complexities of data, honors the pluralism of human experience, and grounds interpretive synthesis in empirical reality.

In summary, cross-referencing empirical datasets operationalizes the 43-profile taxonomy as a *living analytic system*—one that unites conceptual structure with empirical substance. Yet, as the following section explores, the interpretive heart of this framework remains qualitative in orientation: It is through the narrative recontextualization of data that qualitizing achieves its deepest analytic and humanistic significance.

## Beyond the superficial reporting of themes and numbers

Having demonstrated the interpretive and integrative potential of narrative profiling, it is now important to clarify how this approach transcends the superficial reporting practices that often limit both qualitative and quantitative analyses. Too often, qualitative research reports stop at the level of listing themes, presenting them as self-evident discoveries rather than as interpretive constructions. This practice—what Bazeley (2009) termed *superficial reporting*—reduces complex social phenomena to mere categorical labels or frequency counts of codes. In such presentations, themes appear disconnected from context, voice, and meaning, as if the act of identification were itself equivalent to understanding. By focusing on the “what” of findings rather than the “how” or “why,” these accounts risk flattening lived experience into abstract typologies that obscure participants’ intentions, values, and worldviews.

Narrative profiling offers an alternative, integrative response to this methodological limitation. Rather than merely enumerating themes or summarizing responses, it situates data within interpretive storylines that convey depth, nuance, and interconnection. In so doing, it treats qualitative findings not as endpoints but as interpretive pathways—vehicles for meaning-making and sense construction. Through narrative transformation, isolated codes and categories become coherent accounts of agency, motivation, and context, thereby addressing what Bazeley (2009) described as the need to move *beyond description to explanation*. Narrative profiling thus reclaims the interpretive power of qualitizing by framing participants’ perspectives as dynamic narratives that evolve across contexts rather than as static thematic statements.

Similarly, quantitative reporting practices often privilege precision and replicability at the expense of interpretive depth. Tables of means, standard deviations, or regression coefficients are presented as though they speak for themselves, stripped of the human and contextual meanings that underlie the numbers. Such presentations risk treating data as detached abstractions, reinforcing the illusion of objectivity without revealing the lived realities that these numbers represent. Narrative profiling directly challenges this reductionism by recontextualizing quantitative results within interpretive accounts that restore voice, texture, and consequence to statistical trends. When quantitative data are qualitized into narrative profiles, statistical findings are transformed into meaning-laden portraits that illustrate *how* and *why* numerical differences matter in lived experience.

In both cases—whether qualitative or quantitative—narrative profiling transcends superficiality by transforming data into storied meaning systems that integrate pattern and person, number and nuance. Therefore, it bridges postpositivist and interpretivist paradigms, grounding representation in the call of proponents of CDP for reflexivity, inclusion, and contextual understanding. Ultimately, the purpose of narrative profiling is not to replace thematic or statistical analysis, but to elevate both—to move beyond the mere reporting of results toward interpretive synthesis, ethical transparency, and human-centered understanding. Through this reframing, qualitizing becomes not simply a technical transformation, but a moral and epistemological act of rehumanizing data.

## The qualitative orientation of the narrative profiling framework

Although the 43-profile taxonomy has been explicitly designed to serve the integrative purposes of mixed methods inquiry, its interpretive foundation remains distinctly qualitative in orientation. This is not a limitation but a defining strength: It reflects the recognition that integration is ultimately a *meaning-making* act, and meaning—whether derived from numbers, words, images, or experiences—finds its fullest articulation through narrative understanding. The narrative profiling framework thus positions qualitative reasoning not as a *subsidiary mode of analysis*, but as the *interpretive core* through which all forms of data are humanized, contextualized, and rendered intelligible. In this sense, the framework reaffirms what Onwuegbuzie (in press) has emphasized—that qualitative inquiry is not defined by its data type but by its epistemological commitment to understanding the situated, interpretive, and relational dimensions of human experience.

The qualitative orientation of narrative profiling is evident in its emphasis on *context, reflexivity,* and *interpretive depth*. Each narrative profile type—whether derived from quantitative models or qualitative patterns—requires the researcher to engage interpretively with the data’s embedded meanings, relationships, and implications. Even profiles grounded in quantitative origins, such as cluster or predictive profiles, achieve analytical significance only when they are narratively contextualized: when patterns are not merely reported, but *explained* in relation to human processes, conditions, or transformations. Thus, the act of qualitizing is not merely about translating data from one form to another; it is about situating those data within the interpretive ecology of meaning. This interpretive move—what might be termed the *qualitative inflection* of analysis—is what transforms data into knowledge.

Moreover, the framework aligns with what Tracy (2020) terms *qualitative quality*—the capacity of research to evoke understanding through rich description, authenticity, and resonance. Each narrative profile type enacts this quality differently. For instance, experiential and resilience profiles emphasize lived experience and adaptive meaning-making, whereas ethical/moral and value-based profiles extend qualitative inquiry into the moral and axiological domains. Even analytic and structural profiles, which might appear methodologically neutral, acquire qualitative depth when their structures are narratively framed as human processes of categorization, connection, or sense-making. In this way, the taxonomy embeds qualitative sensibility throughout its architecture, ensuring that every act of integration remains grounded in interpretive intentionality.

At a broader epistemological level, the narrative profiling framework advances a *qualitative theory of integration*. It assumes that the synthesis of quantitative and qualitative data is not simply a technical operation but an interpretive one—a process of recontextualization in which data are reimagined through human meaning systems. This orientation resonates with the *CLEAR STEPS* metaphilosophies of CDP 2.0 (Onwuegbuzie and Abrams, 2025; Onwuegbuzie et al., in press)—particularly the theoretical, research, and cultural metaphilosophical dimensions—which collectively conceptualize integration as an interpretive and meaning-centered act. In parallel, it aligns with the *interpretive logic of integration* advanced by Onwuegbuzie (2024c), which asserts that mixed methods research attains its fullest potential when guided by qualitative reasoning as the unifying mode of inference. The 43-profile taxonomy provides this logic with a tangible structure: It operationalizes the idea that all integration, regardless of analytic starting point, culminates in narrative understanding.

Equally central to the framework’s qualitative orientation is the principle of *reflexive transparency*. Narrative profiling explicitly highlights the interpretive agency of the researcher, positioning subjectivity not as a source of bias but as an analytic resource. Each profile type requires the researcher to make visible their narrative positioning—how they interpret data, why they privilege certain connections or transformations, and what interpretive purposes these choices serve. This reflexive visibility transforms analysis into a dialogical act: an ongoing negotiation among the researcher, the data, and the emerging story. In this sense, the framework echoes the *reflexive turn* in qualitative methodology, aligning with scholars such as Alvesson and Sköldberg (2009), who argue that interpretation and self-awareness are inseparable dimensions of rigorous analysis.

The qualitative grounding of narrative profiling also ensures that integration remains *ethical* as well as epistemic (Onwuegbuzie, in press). By requiring interpretive contextualization, the framework resists reductive or dehumanizing uses of data. Profiles such as ethical, critical, and transformative explicitly embed moral reasoning into the analytic process, ensuring that narrative synthesis attends to issues of voice, power, and positionality. Thus, the qualitative orientation of the framework is inseparable from its humanistic ethos: It insists that integration is not merely about coherence between data forms but about coherence between knowledge and values. In this way, the narrative profiling framework extends the qualitative tradition’s commitment to empathy, justice, and reflexivity into the integrative spaces of mixed methods research.

From a methodological standpoint, the framework’s qualitative orientation also provides a *logic of adaptability*. Because qualitative reasoning privileges responsiveness to context, the 43-profile taxonomy remains open to emergent forms of analysis and hybridization. Researchers can modify, combine, or reinterpret profiles as dictated by their empirical realities, maintaining alignment with the qualitative principle of methodological flexibility. This adaptability enables the taxonomy to evolve alongside the field of mixed methods research, continually incorporating new representational strategies, data forms, and interpretive challenges without losing its qualitative anchor.

Finally, the qualitative orientation of narrative profiling reaffirms the philosophical foundation of qualitizing itself: That understanding is always narrative, and that narrative is the medium through which integration becomes possible. Whether the researcher is quantifying experiences or qualitizing numbers, *the interpretive act is inherently narrative*—connecting fragments of data into patterns of meaning that reveal something about human life and experience. Thus, although the 43-profile taxonomy formalizes integration at a methodological level, its deepest contribution is ontological: It reminds us that to integrate is to interpret, and to interpret is to tell a story that honors the complexity, contingency, and coherence of lived experience.

## Interdisciplinary relevance and applications of narrative profiling

### Psychology and allied disciplines

Psychology—with its dual commitment to empirical rigor and interpretive depth—provides an ideal foundation for narrative profiling as an advanced form of qualitizing (Onwuegbuzie and Leech, 2019, 2021). Narrative profiles unify quantitative and qualitative data within the *1 + 1 = 1* integration approach of Onwuegbuzie (2017) and Onwuegbuzie and Hitchcock (2019), transforming statistical indicators and narrative accounts into coherent portraits of human meaning. For instance, a *Resilience Profile* may merge standardized resilience scores with life-history narratives, producing a psychologically rich depiction of adaptation that is both measurable and interpretive.

In clinical and counseling settings, narrative profiles function as qualitized case formulations, integrating quantitative symptom data with qualitative insights on identity reconstruction (McAdams, 2013). Similarly, in developmental and health psychology, *trajectory profiles* and *causal profiles* can trace psychosocial change or recovery processes by linking objective measures with subjective meaning-making. Thus, narrative profiling embodies psychology’s integrative mission—merging measurement with lived experience.

### Applications across disciplines

Beyond psychology, narrative profiling functions as a discipline-transcending methodological form that humanizes data interpretation. In the social sciences, it can blend survey findings with ethnographic accounts (e.g., *marginalized profiles* or *cultural profiles*) to reveal structural and contextual dynamics. In the health sciences, *trajectory profiles* or *resilience profiles* transform clinical data into patient-centered recovery narratives (Greenhalgh, 2016). In education, *developmental profiles* and *contextual profiles* can integrate performance metrics with reflexive journals to illuminate learning processes, whereas in business and organizational studies, *decision-making profiles* can link leadership data with interpretive accounts of action.

Even across the humanities and science, technology, engineering, and mathematics (STEM) fields, narrative profiling provides an analytic bridge—combining numerical modeling with human experience through *ethical*, *value-based*, or *context-sensitive profiles*. Across all domains, the method rests on three unifying principles: *transformation* (data become stories), *integration* (numbers meet meanings), and *humanization* (evidence becomes experience). To enhance conceptual clarity, these three unifying principles can be further understood through the following interpretive formulations: transformation, wherein numbers become narratives through meaning-centered recontextualization; integration, wherein data meet stories through the synthesis of analytic and interpretive logics; and humanization, wherein evidence becomes meaning through the construction of contextually grounded, narrative representations. These formulations are not intended to replace the underlying conceptual framework but rather to provide a more accessible articulation of the processes through which qualitizing operates in practice.

### Integrative significance of narrative profiling

By reframing data as interpretive narratives, narrative profiling extends qualitizing into a broadly adaptable analytic practice. To clarify its role within the broader argument of this article, the integrative significance of narrative profiling lies not merely in its capacity to combine data forms but in its ability to reframe integration as a meaning-centered, representational process. That is, narrative profiling does not simply link quantitative and qualitative components; rather, it transforms them into unified interpretive structures that render complex patterns intelligible within a coherent narrative logic. In this sense, its contribution is both methodological and epistemological—providing a mechanism for integration while also reshaping how integration itself is conceptualized within mixed methods research. For psychology, it operationalizes the integration of measurement and meaning; for other disciplines, it offers a flexible framework for generating story-based meta-inferences that honor both precision and human context—fulfilling the integrative ideal of *1 + 1 = 1*.

## Conclusion

The original definition of *qualitizing*—as the process of converting quantitative data into qualitative data—has made a notable contribution to the field of mixed methods research. However, this definition represents a reductionist characterization of the concept. By framing qualitizing primarily as a procedural act of conversion, it risks overlooking its interpretive, epistemological, and integrative dimensions, thereby obscuring the reflexive and constructionist core of qualitizing. As articulated by Onwuegbuzie and Leech (2019), qualitizing is not merely the transposition of data forms but an act of meaning-making—an interpretive transformation through which numerical structures are recontextualized into narrative, thematic, or conceptual representations that humanize quantitative information. Furthermore, the original definition is not only reductionist, but also narrow in scope because it confines the process of qualitizing exclusively to quantitative data. In contrast, Onwuegbuzie and Leech’s (2019, 2021) five-component model conceptualizes qualitizing as a flexible and recursive process that can originate from quantitative, qualitative, or mixed data sources, thereby broadening its epistemic and methodological reach.

The present article, aptly entitled *On Qualitizing Revisited*, revisits and extends this conceptual lineage by transforming qualitizing from a foundational meta-framework into a multidimensional, interdisciplinary, and philosophically grounded system—anchored in CDP 2.0 and operationalized through a 43-profile narrative taxonomy. In so doing, the article redefines qualitizing as a meta-integrative, meaning-centered process that unites quantitative precision with qualitative depth across research traditions. This framework positions qualitizing not as a unidirectional act of data conversion, but as a multidimensional process of interpretive synthesis that bridges paradigms, deepens integration, and transforms data into understanding.

Although extensive, the 43 narrative profile types are not presented as a definitive or exhaustive enumeration. Rather, they constitute a theoretically grounded and empirically generative framework that captures the principal modes through which data—whether transformed (i.e., quantitized or qualitized) or retained in their original quantitative or qualitative forms—can be narratively integrated. These profiles delineate the current state of representational possibility within qualitizing while remaining open to future expansion as new epistemological, methodological, and technological developments emerge.

Collectively, this re-envisioned framework underscores three central propositions. First, qualitizing is an *act of transformation*—not merely of conversion, but of meaning reconstruction. Second, it is an *act of integration*, dissolving methodological and philosophical divides through the synthesis of numeric and narrative logics. Third, it is an *act of humanization*, restoring context, emotion, and reflexivity to the interpretation of data. Through these dimensions, qualitizing emerges as both a methodological engine and an epistemological stance—one that advances the unification of knowledge under the logic of *1 + 1 = 1* integration.

Ultimately, *On Qualitizing Revisited* situates narrative profiling as the most advanced expression of qualitizing, offering researchers a concrete yet flexible means to generate meta-inferences that transcend traditional analytic divides. By grounding qualitizing in CDP 2.0 and operationalizing it through the 43-profile taxonomy, this article invites a new generation of mixed methods researchers scholars to view integration not merely as the combination of data types, but as the co-construction of meaning. In this sense, qualitizing fulfills its highest purpose: transforming *data into meaning-centered understanding* and *research into reflective interpretation*—recontextualizing analytic outputs within integrative, value-aware frameworks that enhance their human and epistemic significance.

## Data Availability

The data analyzed in this study were taken from a previously published study for which I received Institutional Review Board (IRB) approval. Requests to access these datasets should be directed to AO (tonyonwuegbuzie@aol.com).
